# USP32-mediated stabilization of MAPK12 promotes lung adenocarcinoma progression by inhibiting autophagy-ferroptosis

**DOI:** 10.1038/s42003-026-10429-z

**Published:** 2026-07-19

**Authors:** Qiang Zhang, Hongxu Sheng, Dongnan Ping, Jixiang Gao

**Affiliations:** 1 Department of Thoracic Surgery, School of Medicine, Sir Run Run Shaw Hospital, Zhejiang University, Hangzhou, China; 2 Department of Neurology, Hangzhou TCM Hospital Affiliated to Zhejiang Chinese Medical University, Hangzhou, China

**Keywords:** Apoptosis, Molecular medicine, Non-small-cell lung cancer

## Abstract

Lung adenocarcinoma (LUAD), the most common non-small cell lung cancer, often resists ferroptosis and autophagy—two tumor-suppressive, therapy-sensitive regulated cell death pathways. MAPK12 (a stress-responsive p38 MAPK kinase) boosts LUAD cell survival under oxidative stress, while USP32 (a LUAD-upregulated deubiquitinase) correlates with poor prognosis. However, the USP32–MAPK12 axis’s regulatory role in LUAD ferroptosis and autophagy remains uninvestigated. USP32/MAPK12 expression in LUAD tissues/cell lines was detected via Western blotting and immunohistochemistry. Functional assays (colony formation, Transwell migration, ferroptosis/mitophagy tests) were performed after gene overexpression/knockdown. Protein interactions and ubiquitination were analyzed by co-immunoprecipitation, with in vivo validation using xenograft models. USP32 overexpression in LUAD correlated with reduced overall survival; it stabilized MAPK12 by removing K48-linked ubiquitin chains to block proteasomal degradation. USP32/MAPK12 knockdown activated autophagy/ferroptosis (elevated LC3B/ACSL4/Fe²⁺/MDA, reduced GPX4/p62), inhibited LUAD cell proliferation/migration in vitro and tumor growth in vivo. Thus, targeting the USP32–MAPK12 axis may restore cell death sensitivity, representing a promising LUAD therapeutic strategy.

**Figure:**
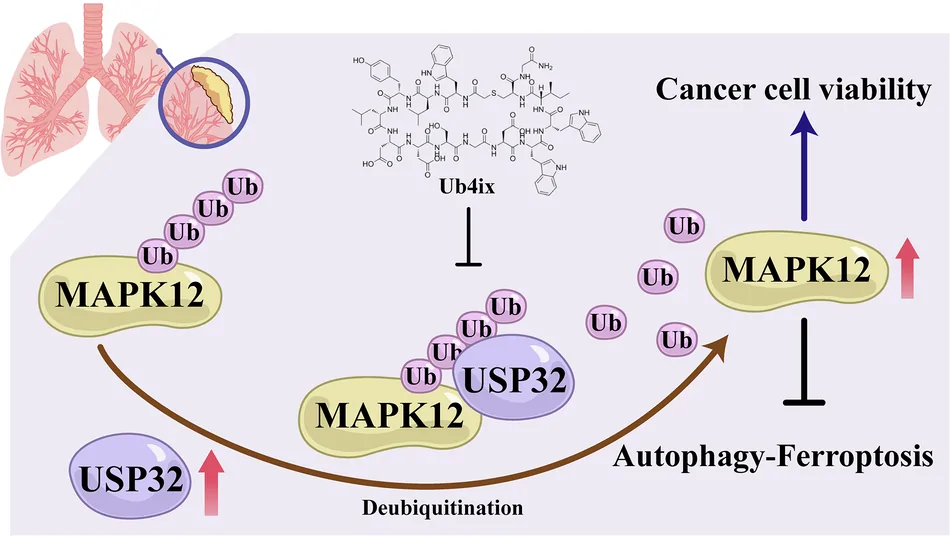

## Abstract (web summary)

Targeting the USP32-MAPK12 axis promotes lung adenocarcinoma progression by stabilizing MAPK12, inhibiting autophagy and ferroptosis. Inhibition of this axis restores cell death sensitivity, offering a potential therapeutic strategy for LUAD.

## Introduction

Lung adenocarcinoma (LUAD), the most common subtype of NSCLC, constitutes approximately 40% of all lung cancer cases^1^. LUAD arises from the glandular epithelial cells in the peripheral lung, lining the alveoli and bronchial structures that produce mucus and maintain airway moisture^2,3^. LUAD affects both smokers and non-smokers and is characterized by a high tendency to metastasize to distant organs such as the bones, liver, and brain^4^. Due to the peripheral location of LUAD tumors, early symptoms are typically mild or absent, resulting in many late-stage diagnoses. LUAD is characterized by high heterogeneity and driven by various genetic mutations, including those in *EGFR*, *KRAS*, and *ALK*^5^. Despite advancements in targeted therapies^6–10^ and immunotherapies^11–15^, resistance to cell death mechanisms such as apoptosis and ferroptosis^1,16,17^ remains a major challenge in LUAD treatment, underscoring the urgent need for further research into the molecular pathways that govern cancer cell survival and resistance.

USP32 (ubiquitin-specific protease 32) functions as a deubiquitinating enzyme that contributes to protein homeostasis by cleaving ubiquitin moieties from substrate proteins, thus shielding them from proteasomal degradation^18^. Previous studies have implicated USP32 in the progression of various cancers by stabilizing oncogenic proteins, promoting cell survival, proliferation, and migration^19–22^. However, its specific role in LUAD, particularly regarding its involvement in regulating cell death pathways such as ferroptosis, remains poorly understood. MAPK12 (Mitogen-Activated Protein Kinase 12), a member of the p38 MAPK family, it participates in regulating cellular processes such as stress adaptation, differentiation, and survival^23–25^. Increasing lines of evidence indicate that MAPK12 contributes to tumor development by facilitating cellular proliferation and metastatic behavior^26,27^. The stabilization of MAPK12 via post-translational modifications, including deubiquitination, may represent a key mechanism through which cancer cells evade stress-induced cell death. Thus, the interaction between USP32 and MAPK12 in LUAD could represent an axis driving tumor survival and therapy resistance.

Deubiquitination is a crucial regulatory process in cancer biology, particularly in the modulation of protein stability and function^28–30^. Aberrant deubiquitination can lead to the accumulation of oncogenic proteins, enabling cancer cells to bypass apoptosis and other forms of programmed cell death. In recent years, autophagy and ferroptosis have emerged as important forms of regulated cell death with significant implications for cancer therapy^31,32^. Autophagy, which recycles damaged cellular components, can either promote cell survival or contribute to cell death, depending on the context^33^. In contrast, ferroptosis represents a distinct mode of cell death driven by iron accumulation and marked by excessive lipid peroxidation^34^. The relationship between autophagy and ferroptosis is complex, with evidence suggesting potential crosstalk between these pathways in regulating cell fate^35–38^.

This study explored how the USP32–MAPK12 signaling axis contributes to ferroptosis resistance in LUAD. Our findings demonstrate that USP32, through deubiquitination, stabilizes MAPK12 in LUAD cells, leading to increased resistance to ferroptosis. Inhibition of the USP32-MAPK12 axis in vitro significantly activated autophagy and ferroptosis, thereby suppressing tumor cell proliferation and migration. Notably, we observed a reciprocal activation relationship between autophagy and ferroptosis in USP32-MAPK12-deficient cells, highlighting the interplay between these pathways. In vivo, targeting the USP32-MAPK12 axis effectively inhibited tumor growth, offering a potential therapeutic strategy for LUAD. This study uncovers a mechanism of ferroptosis resistance in LUAD and provides insights into how autophagy and ferroptosis can be leveraged for therapeutic intervention.

## Results

### Elevated MAPK12 expression is associated with LUAD progression

**Fig. 1:**
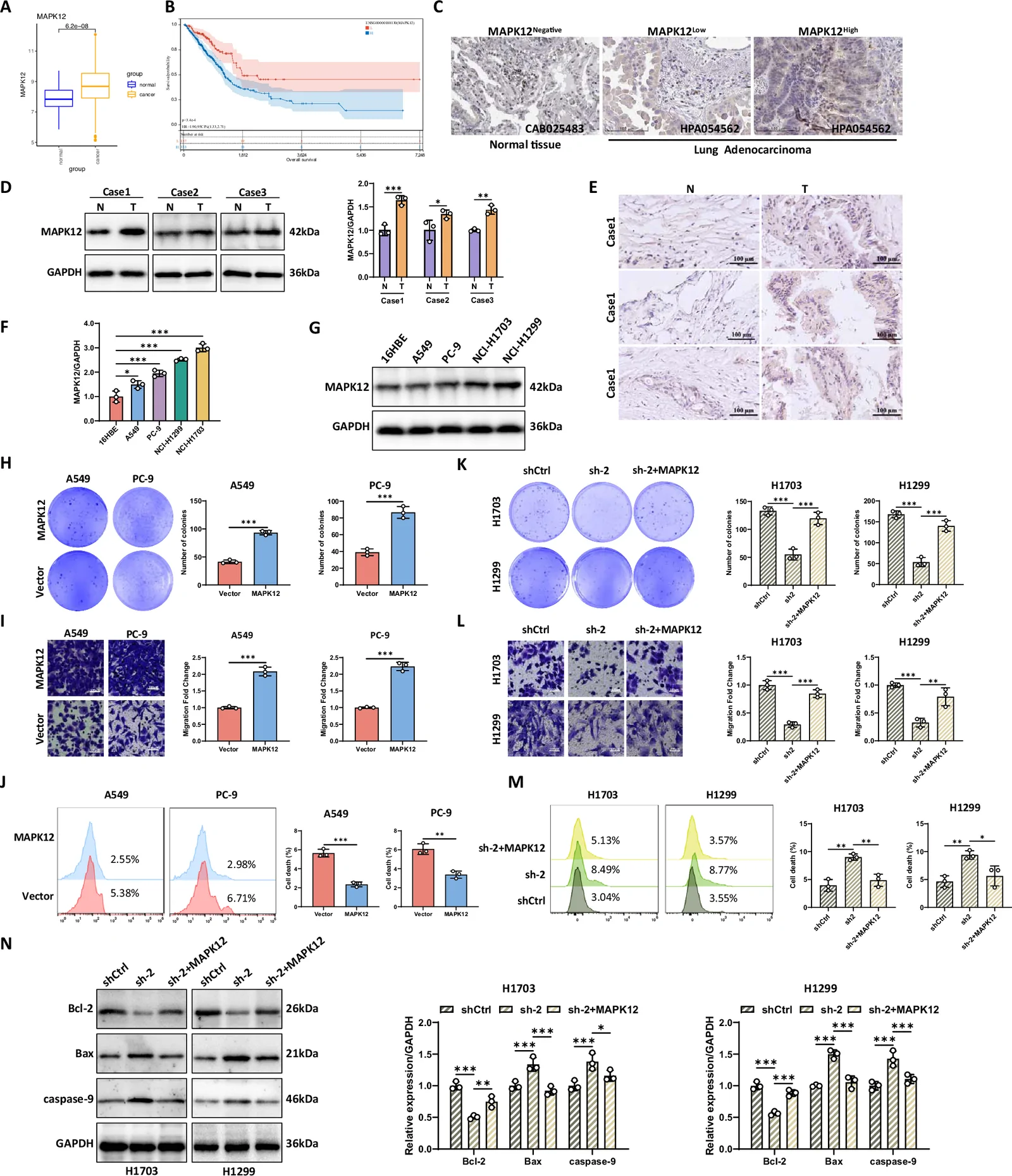
MAPK12 promotes LUAD progression and enhances proliferation and migration of LUAD cells. **A** Box plot showing significantly different MAPK12 expression levels between normal individuals and LUAD patients, based on data from the TCGA database. **B** Kaplan–Meier survival curves illustrate that lung cancer patients with high MAPK12 expression have poorer survival rates compared to those with low expression levels, using the GEPIA 2 database. **C** Representative IHC images display MAPK12 expression in healthy lung tissue versus LUAD tissue, sourced from The Human Protein Atlas. **D** WB analysis and corresponding quantification reveal increased MAPK12 protein levels in tumor tissues (T) compared to adjacent non-tumor tissues (N) from LUAD patients, *n* = 3. **E** IHC staining reveals increased MAPK12 expression in tumor tissues (T) relative to adjacent non-cancerous tissues (N) from LUAD patients. **F** Quantitative PCR analysis indicates higher MAPK12 mRNA levels in LUAD cell lines (A549, PC-9, NCI-H1299, and NCI-H1703) compared to normal bronchial epithelial cells (16HBE), *n* = 3. **G** WB analysis confirms elevated MAPK12 protein levels in LUAD cell lines (A549, PC-9, NCI-H1299, and NCI-H1703) relative to 16HBE cells. **H** Colony formation assays show increased proliferation of A549 and PC-9 cells following MAPK12 overexpression, *n* = 3. **I** Transwell assays demonstrate enhanced migration capacity in these cells due to MAPK12 overexpression, *n* = 3. **J** Flow cytometry with PI staining indicates decreased cell death in A549 and PC-9 cells overexpressing MAPK12. **K** Colony formation assays show that MAPK12 knockdown (sh-2) significantly reduces the proliferative capacity of NCI-H1299 and NCI-H1703 cells, while MAPK12 re-expression (rescue) restores colony-forming ability, *n* = 3. **L** Transwell migration assays demonstrate that MAPK12 knockdown impairs the migratory capacity of NCI-H1299 and NCI-H1703 cells, whereas MAPK12 re-expression reverses this effect, *n* = 3. **M** Flow cytometry analysis with PI staining reveals increased cell death in NCI-H1299 and NCI-H1703 cells upon MAPK12 knockdown, which is mitigated by MAPK12 rescue, *n* = 3. **N** WB analysis of apoptosis-related proteins in NCI-H1299 and NCI-H1703 cells shows that MAPK12 knockdown decreases Bcl-2 and increases Bax and caspase-9 expression, whereas these changes are reversed upon MAPK12 re-expression, *n* = 3. ^*^P < 0.05; ^**^P < 0.01; ^***^P < 0.001; ns, not significant.

*MAPK12* has been identified as a therapeutic target in various cancers^39–42^. Analysis of publicly available datasets revealed that *MAPK12* is significantly overexpressed in lung adenocarcinoma (LUAD) patients (Fig. 1A). Further patient stratification indicated that high *MAPK12* expression is associated with poorer prognosis (Fig. 1B). In addition, data from public imaging databases visually demonstrate the localization of MAPK12 in LUAD tissue samples (Fig. 1C). These observations prompted us to further evaluate MAPK12 expression in our own clinical specimens. Western blotting (WB) and immunohistochemistry (IHC) analyses demonstrated that, despite interpatient variability, MAPK12 expression was consistently elevated in tumor tissues relative to matched adjacent non-tumorous tissues (Fig. 1D, E).

To further explore MAPK12 expression in LUAD models, we examined a panel of lung adenocarcinoma cell lines—A549, PC-9, NCI-H1299, and NCI-H1703—using normal human bronchial epithelial cells (16HBE) as a reference control. WB and qRT-PCR analyses revealed that MAPK12 expression was relatively low in A549 and PC-9 cells, while markedly elevated in NCI-H1299 and NCI-H1703 cells (Fig. 1F, G).

To investigate the functional role of *MAPK12* in LUAD, we first overexpressed *MAPK12* in A549 and PC-9 cells, which exhibit relatively low endogenous MAPK12 expression (Supplementary Fig. 1A). Subsequent colony formation assays, Transwell migration assays, and Flow cytometry with propidium iodide (PI) staining showed that MAPK12 overexpression significantly enhanced the proliferative capacity, migratory ability, and survival of both A549 and PC-9 cells (Fig. 1H–J).

To further validate the function of MAPK12, we designed two *MAPK12*-specific shRNAs. qPCR and WB analyses confirmed that *shMAPK12-2* (sh-2) achieved more effective knockdown compared to sh-1 (Supplementary Fig. 1B, C). Importantly, knockdown specificity was confirmed by WB of MAPK11 and MAPK14, two homologs of MAPK12, whose expression remained unchanged, indicating no detectable off-target effects (Supplementary Fig. 1D). Therefore, sh-2 was used in all subsequent knockdown experiments.

We then performed rescue experiments by re-expressing MAPK12 in NCI-H1299 and NCI-H1703 cells following sh-2-mediated knockdown. Colony formation experiments revealed that silencing *MAPK12* markedly inhibited cellular proliferation, whereas reintroduction of MAPK12 rescued the proliferative ability (Fig. 1K). Similarly, Transwell assays showed that *MAPK12* knockdown impaired cell migration, which was also rescued upon MAPK12 re-expression (Fig. 1L). Flow cytometry with PI staining revealed a marked increase in cell death following *MAPK12* knockdown, whereas this effect was largely reversed in the rescue group (Fig. 1M).

WB analysis of apoptosis-associated proteins confirmed that *MAPK12* silencing in NCI-H1299 and NCI-H1703 cells induced apoptotic activation, as evidenced by decreased levels of Bcl-2 and increased expression of the Bax and caspase-9. These changes were reversed upon re-expression of *MAPK12* (Fig. 1N), suggesting that MAPK12 depletion in LUAD may initiate apoptosis, a phenotype that is consistent with observations reported in nasopharyngeal carcinoma cells^43^.

### MAPK12 inhibits mitophagy in LUAD cells

**Fig. 2:**
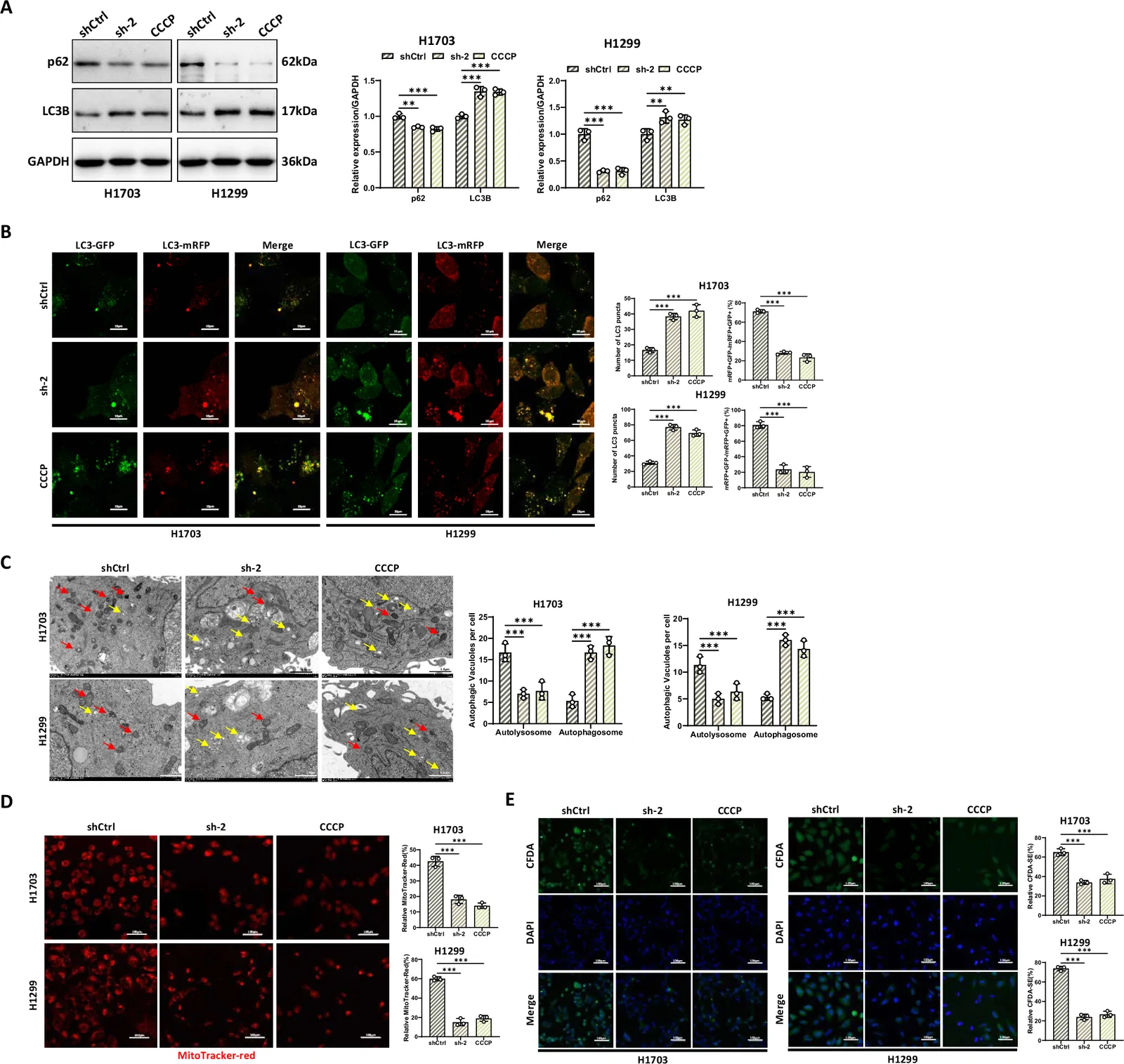
MAPK12 knockdown induces mitophagy and cell death in LUAD cells. **A** WB analysis shows altered expression levels of autophagy-related proteins p62 and LC3B in NCI-H1703 and NCI-H1299 cells after MAPK12 knockdown using sh-2, *n* = 3. **B** mRFP-GFP-LC3 dual fluorescence imaging demonstrates increased numbers of autophagosomes and autolysosomes, as well as a higher ratio of autolysosomes to autophagosomes, in NCI-H1703 and NCI-H1299 cells following MAPK12 knockdown, *n* = 3. **C** TEM images reveal increased autophagosomes (yellow arrows) and reduced autolysosomes (red arrows) in NCI-H1703 and NCI-H1299 cells post-MAPK12 knockdown, *n* = 3. **D** MitoTracker staining visualizes mitochondrial morphology in NCI-H1703 and NCI-H1299 cells before and after MAPK12 knockdown, *n* = 3. **E** CFDA and DAPI staining indicate decreased cell viability in NCI-H1703 and NCI-H1299 cells following MAPK12 knockdown, *n* = 3. ^**^P < 0.01; ^***^P < 0.001.

Notably, silencing *MAPK12* resulted in a pronounced decrease in the autophagy adaptor protein and negative regulator p62, along with substantial accumulation of LC3B, a marker associated with autophagosome membranes (Fig. 2A). This pattern closely resembled that observed in CCCP-treated cells, suggesting that MAPK12 deficiency may activate mitophagy in LUAD cells. *MAPK12* knockdown markedly increased the abundance of autophagy-related subcellular structures, as evidenced by rapid accumulation of autophagosomes (Fig. 2B). Transmission electron microscopy (TEM) provided additional confirmation, revealing a higher abundance of double-membrane autophagosomes in cells with *MAPK12* silencing (Fig. 2C). MitoTracker staining revealed a marked reduction in healthy mitochondria in LUAD cells following *MAPK12* knockdown (Fig. 2D), suggesting that the upregulation of autophagy may be driven by a need to remove damaged mitochondria.

To further evaluate cell viability, we performed CFDA staining, which selectively labels viable cells with green fluorescence. In agreement with the PI-based flow cytometry findings (Fig. 1L), CFDA staining revealed a marked reduction in viable cell numbers following *MAPK12* silencing combined with CCCP treatment (Fig. 2E). Conversely, *MAPK12* overexpression in A549 and PC-9 cells reduced autophagy levels (Supplementary Fig. 1E, F), increased the number of healthy mitochondria (Supplementary Fig. 1G), and enhanced cell survival (Supplementary Fig. 1H).

### MAPK12 inhibits ferroptosis in LUAD cells

**Fig. 3:**
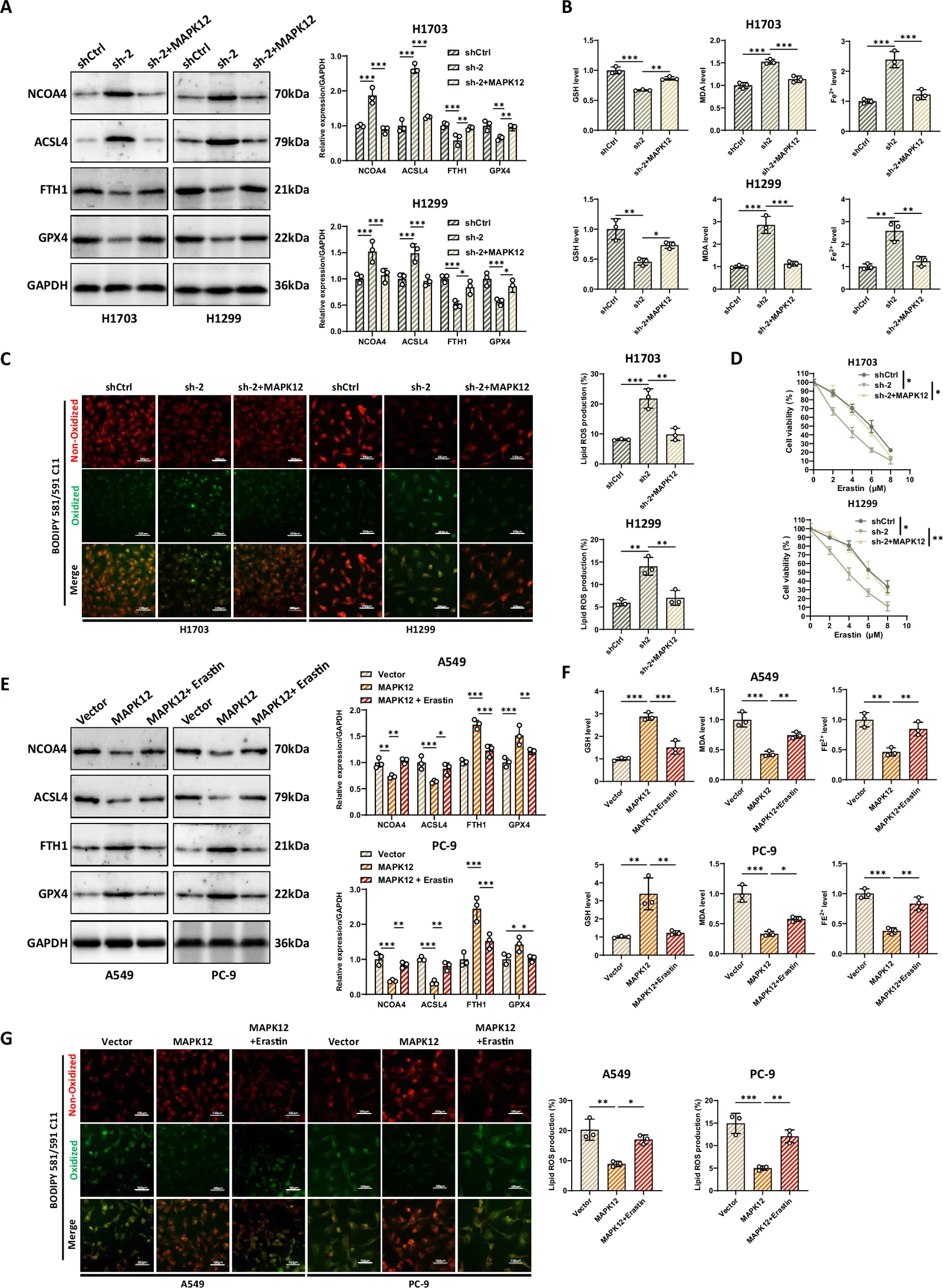
Regulation of MAPK12 Mediates Ferroptosis in LUAD Cells. **A** WB analysis reveals that MAPK12 knockdown affects the expression of ferroptosis-related proteins in LUAD cells, *n* = 3. **B** Biochemical assays show that MAPK12 knockdown alters levels of ferroptosis markers, including GSH, MDA, and Fe^2+^, in LUAD cells, *n* = 3. **C** BODIPY C11 staining indicates increased ROS levels in LUAD cell lines after MAPK12 knockdown. Red fluorescence represents the non-oxidized probe, while green fluorescence indicates oxidation, reflecting lipid peroxidation levels, *n* = 3. **D** CCK-8 assays demonstrate decreased cell viability in MAPK12-knockdown LUAD cell lines following two hours of treatment with various concentrations of the ferroptosis inducer Erastin, *n* = 3. **E** WB analysis shows that MAPK12 overexpression influences the expression of ferroptosis-related proteins in LUAD cells, *n* = 3. **F** Biochemical assays reveal changes in ferroptosis markers (GSH, MDA, and Fe^2+^) in LUAD cells due to MAPK12 overexpression, *n* = 3. **G** BODIPY C11 staining reflects decreased ROS levels in LUAD cell lines after MAPK12 overexpression, *n* = 3. ^*^P < 0.05; ^***^P < 0.001.

WB analysis revealed that *MAPK12* knockdown resulted in upregulation of nuclear receptor coactivator 4 (NCOA4), a key mediator of ferritinophagy, indicating enhanced ferritin degradation. Consistently, the expression of ferritin heavy chain 1 (FTH1), a major component of the ferritin complex responsible for iron storage, was significantly reduced. Furthermore, silencing *MAPK12* led to a pronounced upregulation of acyl-CoA synthetase long-chain family member 4 (ACSL4), an essential enzyme involved in generating polyunsaturated fatty acids susceptible to lipid peroxidation, while the expression of glutathione peroxidase 4 (GPX4), a key lipid peroxide-eliminating enzyme, was significantly reduced (Fig. 3A). These molecular changes collectively point toward a ferroptotic phenotype induced by MAPK12 depletion.

In addition to changes in ferroptosis-related protein expression, we further assessed key biochemical markers of ferroptosis. *MAPK12* knockdown led to a significant increase in intracellular Fe^2+^ levels, accompanied by a reduction in GSH and a marked elevation of lipid peroxidation products (Fig. 3B). To visualize lipid ROS, we employed the C11-BODIPY fluorescent probe, which shifts from red to green fluorescence upon oxidation. LUAD cells with *MAPK12* knockdown exhibited a substantial enhanced lipid peroxidation (Fig. 3C).

To evaluate whether MAPK12 modulates cellular sensitivity to ferroptosis induction, we treated LUAD cells with increasing concentrations of Erastin, a canonical ferroptosis inducer, followed by CCK-8 viability assays after 2 h. MAPK12-deficient cells exhibited a markedly increased susceptibility to Erastin-induced cell death compared with control and MAPK12-rescued counterparts (Fig. 3D). These findings suggest that MAPK12 depletion sensitizes LUAD cells to ferroptotic stress.

WB analysis revealed that, compared to control and Erastin-treated groups, *MAPK12* overexpression significantly suppressed the expression of NCOA4 and ACSL4, two key promoters of ferroptosis, while elevating the levels of FTH1 and GPX4 (Fig. 3E). These findings indicate a reduction in lipid peroxidation and an enhancement in iron homeostasis and antioxidant capacity following *MAPK12* overexpression. Supporting these findings, biochemical analyses revealed that *MAPK12* overexpression led to elevated intracellular GSH levels, a decrease in MDA—an indicator of lipid peroxidation—and a reduction in free Fe^2+^ concentration Biochemical assays supported these observations: *MAPK12* overexpression increased intracellular GSH accumulation, reduced the levels of MDA, a marker of lipid peroxidation, and lowered the concentration of free Fe^2+^ (Fig. 3F). Additionally, C11-BODIPY staining showed a substantial reduction in lipid-ROS levels in *MAPK12*-overexpressing cells, and this reduction antagonized the pro-ferroptotic effects of Erastin (Fig. 3G).

### MAPK12 mediates crosstalk between ferroptosis and autophagy

**Fig. 4:**
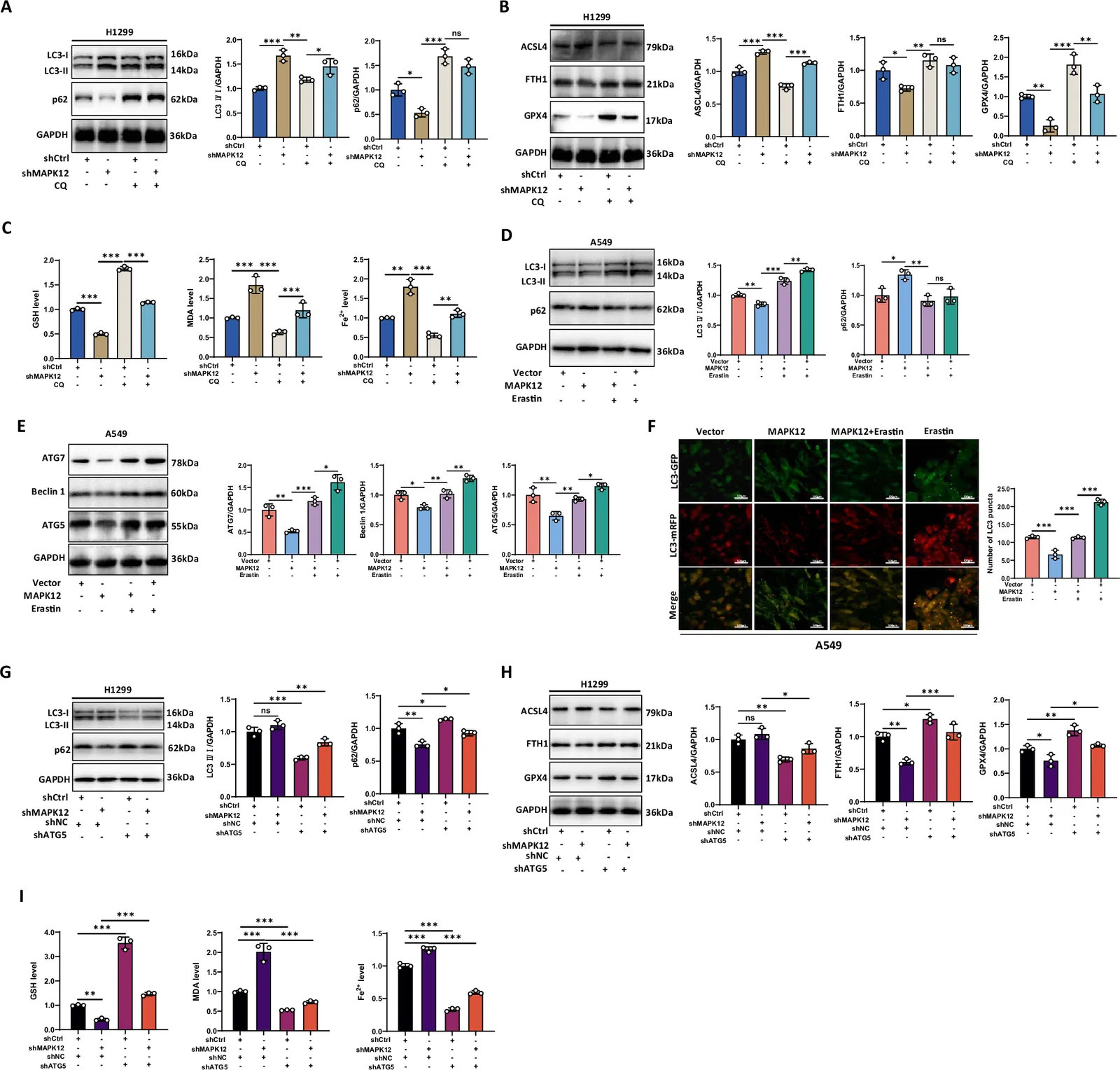
MAPK12 mediates crosstalk between autophagy and ferroptosis. **A**–**C***NCI-H1299 cells with MAPK12 knockdown and CQ treatment:*** A** WB analysis showing the accumulation of autophagy-related proteins LC3-I/II and p62, indicating autophagosome maturation, *n* = 3. **B** WB displaying expression levels of ferroptosis-related proteins ACSL4, FTH1, and GPX4, *n* = 3. **C** Biochemical assays measuring relative levels of ferroptosis markers GSH, MDA, and Fe^2+^ compared to control, *n* = 3. **D**–**F***A549 cells overexpressing OTUB2 with Erastin treatment:*** D** WB showing the expression of autophagy-related proteins LC3-I/II and p62, *n* = 3. **E** WB analysis of autophagy initiation and formation-related proteins ATG7, Beclin 1, and ATG5, *n* = 3. **F** mRFP-GFP-LC3 dual fluorescence imaging demonstrating autophagy levels and the ratio of autolysosomes to autophagosomes, *n* = 3. **G**–**I** *NCI-H1299 cells with MAPK12 and/or ATG5 knockdown*:** G** WB showing the expression of autophagy-related proteins LC3-I/II and p62, *n* = 3. **H** WB displaying expression levels of ferroptosis-related proteins ACSL4, FTH1, and GPX4, *n* = 3. **I** Biochemical assays measuring relative levels of ferroptosis markers GSH, MDA, and Fe^2+^ compared to control, *n* = 3. ^**^P < 0.01; ^***^P < 0.001.

We employed chloroquine (CQ) to determine the functional role of autophagy in MAPK12-mediated ferroptosis. WB analysis revealed that CQ treatment led to significant accumulation of p62 in NCI-H1299 cells, and this accumulation was not affected by *MAPK12* knockdown (Fig. 4A). This suggests that CQ effectively inhibits autophagic degradation in these cells, independent of MAPK12 status. The concurrent accumulation of LC3 further confirmed that the inhibition occurred at the degradation stage of autophagy, rather than at the level of autophagosome formation.

Interestingly, CQ treatment also resulted in elevated GPX4 protein levels, indicating that blockade of autophagy impairs GPX4 degradation. However, this CQ-induced accumulation of GPX4 was significantly reduced by *MAPK12* knockdown (Fig. 4B), suggesting that *MAPK12* knockdown-induced autophagy facilitates the degradation of antioxidant proteins such as GPX4. Inhibition of autophagy by CQ attenuated ferroptosis, as evidenced by reduced levels of Fe^2+^ and lipid peroxidation; however, this cytoprotective effect was abolished upon *MAPK12* silencing (Fig. 4C). Collectively, these results imply that MAPK12 predominantly influences the formation of autophagosomes rather than their degradation. As sequestration of proteins and organelles within autophagosomes functionally inactivates them, MAPK12-driven autophagy may promote ferroptosis by targeting intracellular antioxidant systems such as GPX4 for degradation.

We next investigated whether ferroptosis induction affects autophagy activation. Exposure of A549 cells to Erastin resulted in reduced p62 levels and a pronounced upregulation of autophagy-related proteins, including LC3, ATG5, ATG7, and Beclin 1, suggesting increased autophagic flux (Fig. 4D, E). However, WB and mRFP-GFP-LC3B dual-fluorescence imaging revealed that *MAPK12* overexpression attenuated Erastin-induced autophagy at the autophagosome formation stage (Fig. 4E, F), further supporting the notion that MAPK12 negatively regulates autophagy initiation.

ATG5 silencing reduced LC3 expression and increased p62 accumulation, confirming the inhibition of autophagy (Fig. 4G). Notably, silencing ATG5 led to a reduction in the pro-ferroptotic protein ACSL4 and an elevation in the anti-ferroptotic markers FTH1 and GPX4, indicating an inhibitory effect on ferroptosis (Fig. 4H).

Interestingly, analysis of ferroptosis-related biochemical markers revealed differential regulatory strength between MAPK12 and ATG5. The decrease in intracellular GSH levels caused by *MAPK12* knockdown was notably weaker than the increase in GSH levels observed upon *ATG5* knockdown. Similarly, the elevation in free Fe^2+^ levels induced by *MAPK12* silencing was less pronounced than the reduction observed following *ATG5* silencing. Moreover, in cells with simultaneous *MAPK12* and *ATG5* knockdown, the rescue of MDA levels by MAPK12 deficiency failed to reach the extent of MDA elevation seen in *MAPK12* knockdown alone (Fig. 4I).

### USP32 deubiquitinates and stabilizes MAPK12 in LUAD cells

**Fig. 5:**
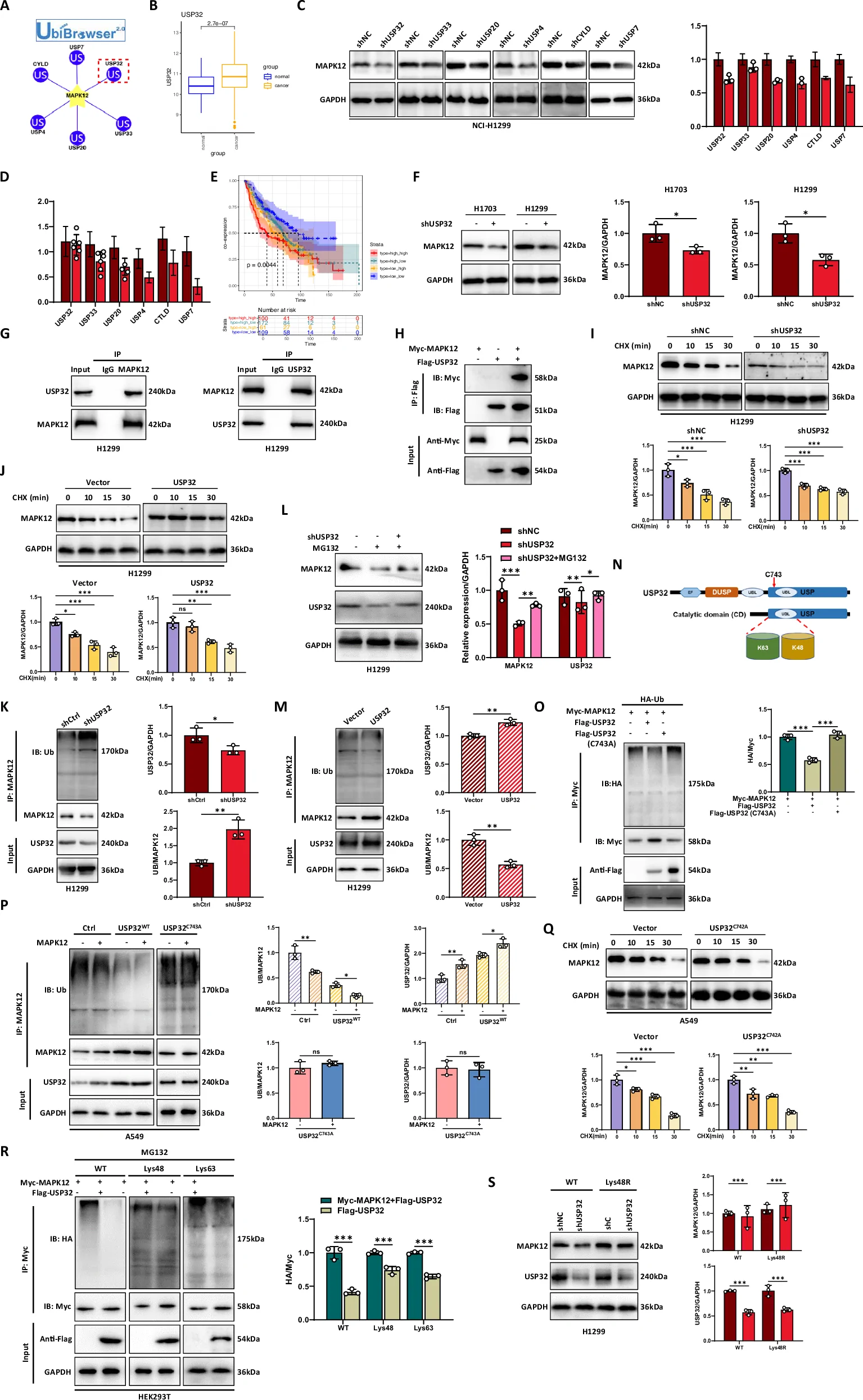
USP32 deubiquitinates and stabilizes MAPK12 in LUAD cells. **A** Correlation analysis identifies potential DUBs related to MAPK12, including USP32, USP33, USP20, USP7, USP4, and CYLD, using the UbiBrowser 2.0 database. **B** Box plot showing USP32 expression levels in normal individuals and LUAD patients based on TCGA dataset. **C** WB analysis of MAPK12 protein levels in NCI-H1299 cells following siRNA-mediated knockdown of USP32, USP33, USP20, USP7, USP4, and CYLD, *n* = 3. **D** Quantitative PCR analysis indicates that MAPK12 transcription levels remain unchanged following knockdown of these DUBs, suggesting post-translational regulation, *n* = 6. **E** Kaplan–Meier survival analysis indicates that co-high expression of USP32 and MAPK12 is associated with poor prognosis. The groups are defined as follows: “high_high” represents high expression of both MAPK12 and USP32, “high_low” represents high expression of MAPK12 and low expression of USP32, “low_high” represents low expression of MAPK12 and high expression of USP32, and “low_low” represents low expression of both MAPK12 and USP32. The number of patients at risk is shown below the plot, corresponding to each time point. **F** In NCI-H1299 and NCI-H1703 cells with high USP32 expression, USP32 knockdown leads to decreased MAPK12 protein levels, *n* = 3. **G** Co-IP assays demonstrate that USP32 physically interacts with MAPK12 in NCI-H1299 cells. **H** In HEK293T cells co-transfected with Myc-MAPK12 and Flag-USP32, IP confirms the interaction between USP32 and MAPK12. **I** WB analysis reveals that MAPK12 degradation is accelerated in USP32-knockdown NCI-H1299 cells treated with CHX over time, *n* = 3. **J** WB analysis reveals that MAPK12 degradation is slowed in USP32-overexpressing NCI-H1299 cells treated with CHX over time, *n* = 3. **K** IP assays show increased ubiquitination of MAPK12 in USP32-knockdown NCI-H1299 cells, *n* = 3. **L** Treatment with the proteasome inhibitor MG132 restores MAPK12 levels in USP32-knockdown NCI-H1299 cells, indicating proteasomal degradation, *n* = 3. **M** In USP32-overexpressing NCI-H1299 cells, IP assays reveal reduced ubiquitination of MAPK12, *n* = 3. **N** A schematic diagram illustrates the structure of USP32, highlighting the catalytic domain. **O** In HEK293T cells, the catalytically inactive mutant USP32 (C743A) fails to reduce MAPK12 ubiquitination compared to wild-type USP32, *n* = 3. **P** WB analysis shows that overexpression of catalytically inactive USP32 (C743A) does not stabilize MAPK12 in A549 cells after 72 h, *n* = 3. **Q** MAPK12 degradation remains rapid in A549 cells expressing USP32 C743A mutant treated with CHX, *n* = 3. **R** Ubiquitination assays demonstrate that USP32 specifically removes Lys48-linked, but not Lys63-linked, ubiquitin chains from MAPK12, thereby regulating its protein stability, *n* = 3. **S** WB analysis of NCI-H1299 cells expressing wild-type (WT) or Lys48R mutant ubiquitin shows that USP32 knockdown reduces MAPK12 protein levels in the presence of WT ubiquitin, but not in the presence of Lys48R ubiquitin, *n* = 3. ^*^P < 0.05; ^**^P < 0.01; ^***^P < 0.001.

Bioinformatics analysis identified six candidate deubiquitinases (DUBs) that may regulate MAPK12 stability via the ubiquitin–proteasome pathway: USP32, USP33, USP20, USP7, USP4, and CYLD (Fig. 5A).

Among these, *USP32* (Fig. 5B), *USP33* (Supplementary Fig. 2A), *USP20* (Supplementary Fig. 2B), *USP7* (Supplementary Fig. 2C), and *USP4* (Supplementary Fig. 2D) were significantly upregulated in LUAD patients, whereas *CYLD* (Supplementary Fig. 2E) showed no significant difference in expression between normal and tumor tissues. To evaluate the functional relevance of these DUBs, we performed shRNA-mediated knockdown of each candidate gene in NCI-H1299 cells and subsequently assessed MAPK12 protein levels. Notably, silencing of *USP32*, *USP20*, *USP7*, and *USP4* resulted in a marked reduction of MAPK12 protein abundance (Fig. 5C). Among them, only *USP32* knockdown did not alter MAPK12 mRNA levels, as shown by qPCR analysis (Fig. 5D), suggesting that USP32 regulates MAPK12 expression primarily at the post-translational level.

Furthermore, correlation analysis revealed a positive transcriptional correlation between *USP32* and *MAPK12* expression in LUAD (Supplementary Fig. 2F), indicating the potential for their co-upregulation in patients, which may synergistically enhance MAPK12 protein stability. Consistently, Kaplan–Meier survival analysis demonstrated that LUAD patients with co-high expression of *USP32* and *MAPK12* exhibited the poorest overall survival (Fig. 5E).

We further validated these findings in NCI-H1703 cells. Consistent with the observations in NCI-H1299 cells, *USP32* knockdown significantly decreased MAPK12 protein levels (Fig. 5F). Co-immunoprecipitation (Co-IP) experiments revealed that endogenous MAPK12 co-precipitated with USP32 and vice versa, indicating a specific interaction between the two proteins (Fig. 5G). To exclude the possibility of non-specific antibody binding, we co-expressed Myc-tagged *MAPK12* and Flag-tagged *USP32* in HEK293T cells. Reciprocal Co-IP assays using anti-Flag and anti-Myc antibodies followed by WB confirmed that exogenous USP32 and MAPK12 directly interact with each other (Fig. 5H).

To evaluate the stability of MAPK12 protein, we treated NCI-H1299 cells with the protein synthesis inhibitor cycloheximide (CHX) to block MAPK12 synthesis and monitored the degradation of existing MAPK12 over time. However, due to markedly reduced baseline levels of MAPK12 in USP32-depleted cells, it was difficult to assess the degradation kinetics or determine the protective effect of USP32 (Fig. 5I). To further probe the mechanism, we treated USP32 knockdown cells with the proteasome inhibitor MG132. This intervention partially restored MAPK12 protein levels, suggesting that the reduction in MAPK12 observed upon USP32 knockdown is mediated via the ubiquitin–proteasome degradation pathway (Fig. 5J).

We next performed in vitro ubiquitination assays in NCI-H1299 cells under conditions of USP32 knockdown or overexpression. Knockdown of *USP32* led to increased polyubiquitination of MAPK12, whereas overexpression of *USP32* markedly reduced MAPK12 ubiquitination, correlating with elevated MAPK12 protein levels (Fig. 5K–M). These findings demonstrate that USP32 stabilizes MAPK12 by removing ubiquitin chains and thereby preventing proteasomal degradation.

USP32 is a multi-domain deubiquitinase comprising a calcium-binding EF-hand domain, a DUSP domain, a catalytic USP domain, two ubiquitin-like (UBL) domains, and a C-terminal CAAX motif involved in prenylation. The USP catalytic domain cleaves ubiquitin chains through the catalytic cysteine residue at position 743 (C743) (Fig. 5N). To investigate the requirement of USP32’s enzymatic function in modulating MAPK12, we generated a catalytically inactive mutant, USP32^C743A^, tagged with Flag, and expressed it in HEK293T cells. Co-IP assays showed that, in contrast to wild-type USP32, the USP32^C743A^ mutant failed to reduce MAPK12 ubiquitination and did not alter the amount of Myc-MAPK12 co-precipitated (Fig. 5O), indicating that loss of catalytic activity abolishes the deubiquitinating function of USP32 without affecting protein–protein binding.

We confirmed these findings in A549 cells, where expression of USP32^C743A^ also had no impact on MAPK12 ubiquitination levels (Fig. 5P). Furthermore, CHX chase assays in A549 cells demonstrated that the expression of USP32^C743A^ did not alter the degradation rate of MAPK12 compared to the control group, further confirming that the catalytic cysteine-743 is indispensable for USP32-mediated stabilization of MAPK12 (Fig. 5Q).

To elucidate the specific ubiquitin linkage removed by USP32 during MAPK12 deubiquitination, HEK293T cells were transfected with a panel of HA-tagged recombinant ubiquitin variants, including wild-type ubiquitin (HA-Ub-WT), K48-only (HA-Ub-K48-only), and K63-only (HA-Ub-K63-only) constructs. In the presence of USP32, MAPK12 ubiquitination was markedly reduced in cells expressing WT or K48-only ubiquitin, whereas K63-linked ubiquitination was unaffected (Fig. 5R). These results indicate that USP32 selectively targets Lys48-linked polyubiquitin chains.

To further validate this specificity, we introduced a Lys48-resistant ubiquitin mutant (Lys48R) into NCI-H1299 cells. Under conditions of *USP32* knockdown, MAPK12 protein levels were reduced in cells expressing wild-type ubiquitin, but this effect was abolished when ubiquitin Lys48R was expressed (Fig. 5S).

### USP32-MAPK12 axis promotes LUAD Progression by inhibiting autophagy and ferroptosis

**Fig. 6:**
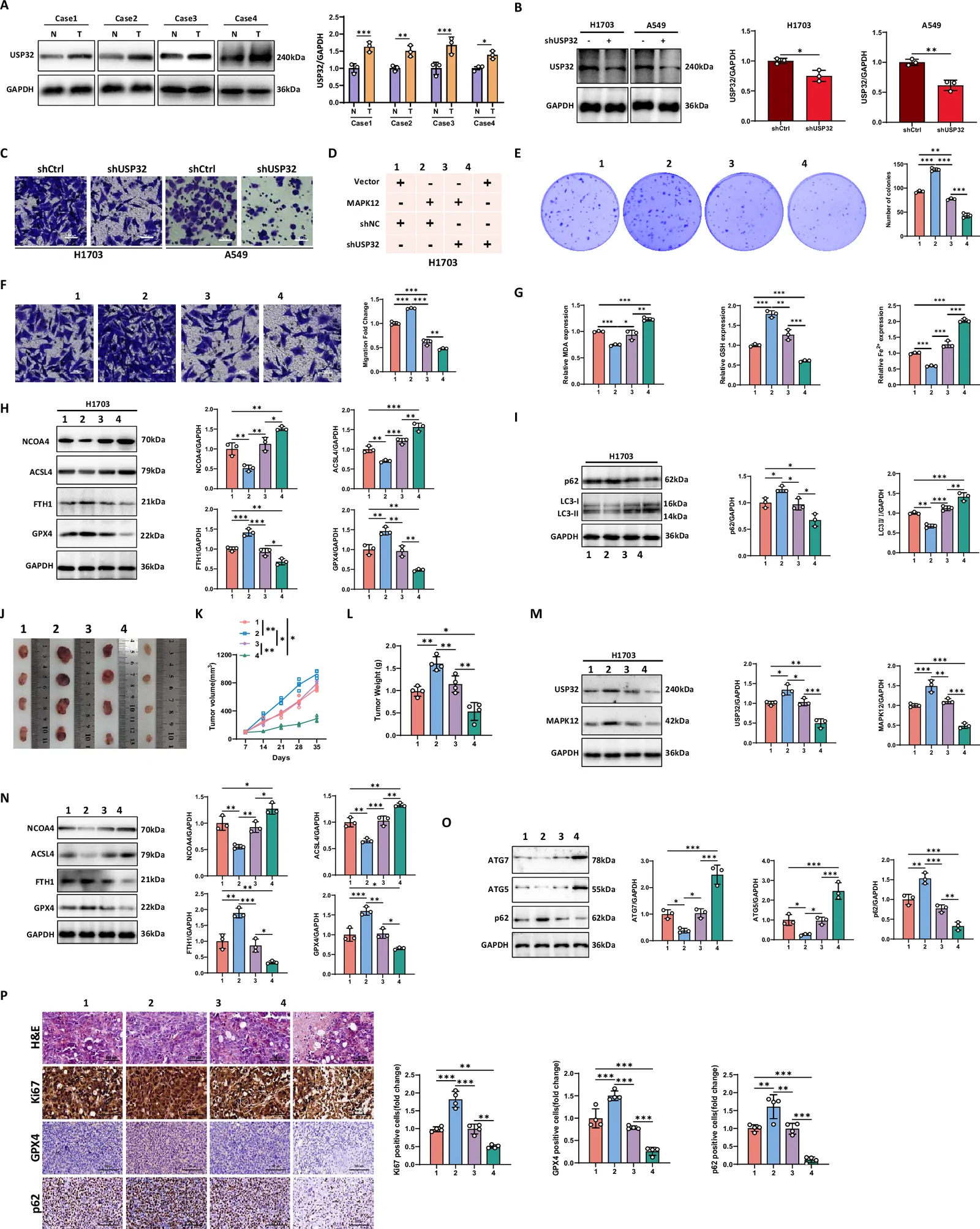
Upregulation of MAPK12 inhibits ferroptosis and enhances tumor cell viability in vitro and in vivo*.* **A** WB analysis shows higher USP32 expression in tumor tissues (T) compared to adjacent non-cancerous tissues (N) from LUAD patients, *n* = 3. **B** Knockdown of USP32 in NCI-H1703 and A549 cells reduces USP32 protein levels, *n* = 3. **C** Transwell assays demonstrate that USP32 knockdown diminishes the migration ability of NCI-H1703 and A549 cells. **D** NCI-H1703 cells were treated with MAPK12 overexpression and/or USP32 knockdown. **E** Colony formation assays show that MAPK12 overexpression restores colony-forming ability in NCI-H1703 cells following USP32 knockdown, *n* = 3. **F** Transwell migration assays show that MAPK12 overexpression restores the migratory capacity of NCI-H1703 cells reduced by USP32 knockdown, *n* = 3. **G** Biochemical assays indicate that MAPK12 overexpression negates the increase in ferroptosis markers (MDA, GSH, and Fe^2+^) induced by USP32 knockdown, *n* = 3. **H** WB analysis shows that MAPK12 overexpression restores ferroptosis-related proteins NCOA4, ACSL4, FTH1, and GPX4 affected by USP32 knockdown, *n* = 3. **I** Autophagy-related proteins p62 and LC3 I/II levels are also modulated by MAPK12 overexpression in the context of USP32 knockdown, *n* = 3. **J** Representative images of tumors formed by NCI-H1703 cells in nude mice after 35 days under different treatments highlight the in vivo relevance. **K** Tumor growth curves show that MAPK12 overexpression accelerates tumor growth despite USP32 knockdown, *n* = 4. **L** Average tumor weights indicate that MAPK12 overexpression mitigates the tumor-suppressive effects of USP32 knockdown, *n* = 4. **M** WB analysis shows the expression levels of USP32 and MAPK12 in tumor tissues from different treatment groups, *n* = 3. **N** WB analysis of tumor tissues shows the expression levels of ferroptosis-related proteins, including NCOA4, ACSL4, FTH1, and GPX4, *n* = 3. **O** WB analysis of autophagy-related proteins (ATG7, ATG5, and p62) in tumor tissues reflects similar trends as observed in vitro, *n* = 3. **P** Histological staining of tumor sections using H&E, along with immunohistochemistry for Ki-67, GPX4, and p62, shows treatment-dependent changes in tumor cell morphology and proliferation, *n* = 4. ^*^P < 0.05; ^**^P < 0.01; ^***^P < 0.001.

Examination of clinical samples showed that USP32 expression was markedly elevated in tumor tissues relative to matched adjacent non-tumor tissues (Fig. 6A). Given our prior findings that USP32 stabilizes MAPK12 through deubiquitination, we further explored the functional role of the USP32–MAPK12 axis in LUAD. To this end, we designed and validated shRNA constructs targeting *USP32* (*shUSP32*) for gene silencing (Fig. 6B) and a full-length *USP32* overexpression construct (Supplementary Fig. 3A). Transwell migration experiments revealed that silencing USP32 markedly impaired the migratory ability of NCI-H1703 and A549 lung adenocarcinoma cells (Fig. 6C).

To directly investigate the role of the USP32–MAPK12 axis, we modulated its activity by co-transfecting cells with *shUSP32* and/or a *MAPK12* overexpression plasmid (Fig. 6D). Colony formation analysis indicated that activation of the USP32–MAPK12 axis promoted cell proliferation, while knockdown of either USP32 or MAPK12 significantly reduced clonogenic potential (Fig. 6E). Similarly, overexpression of MAPK12 promoted LUAD cell migration, while *USP32* knockdown had the opposite effect, indicating that both proteins contribute to the metastatic potential of LUAD cells (Fig. 6F).

To further investigate the mechanistic role of the USP32–MAPK12 axis, we assessed ferroptosis-related biochemical markers and protein expression. The levels of MDA, Fe^2+^, and ACSL4—markers positively associated with ferroptosis—were decreased following *MAPK12* overexpression but elevated upon *USP32* inhibition (Fig. 6G, H). In contrast, ferroptosis-resistance indicators such as GSH, FTH1, and GPX4 increased with *MAPK12* upregulation and decreased with *USP32* knockdown (Fig. 6H). Notably, the expression trend of the ferritinophagy mediator NCOA4 paralleled that of ACSL4, further supporting the axis’s role in regulating iron metabolism and ferroptotic signaling. WB analysis of autophagy-related proteins revealed that upregulation of the USP32–MAPK12 axis led to p62 accumulation and decreased LC3 levels, suggesting autophagy inhibition. Conversely, suppression of this axis promoted p62 degradation and increased LC3 expression, indicating autophagy activation (Fig. 6I).

To evaluate the in vivo function of the USP32–MAPK12 axis, we generated stable LUAD cell lines with lentiviral-mediated *MAPK12* overexpression and/or *USP32* knockdown. These cells were subcutaneously implanted into immunodeficient mice to establish xenograft models (Fig. 6J). Tumor volume was monitored weekly using caliper measurements (Fig. 6K), and tumor weights were recorded at endpoint following animal euthanasia (Fig. 6L). WB of tumor lysates confirmed the in vivo consistency of *MAPK12* upregulation and *USP32* knockdown effects observed in vitro (Fig. 6M).

Further analysis of ferroptosis- and autophagy-related proteins demonstrated that the USP32–MAPK12 axis also modulated these pathways in vivo (Fig. 6N, O). Histological examination using H&E and immunohistochemical staining revealed that higher MAPK12 expression correlated with increased tumor cellular density, elevated GPX4 and p62 accumulation, and higher levels of the proliferation marker Ki67 (Fig. 6P). In contrast, knockdown of *MAPK12* or inhibition of *USP32* resulted in suppressed tumor growth, reduced tumor weight, and the presence of vacuolated regions within tumors indicative of cell death, alongside a decrease in GPX4 and p62 expressions.

The expression patterns of ferroptosis-related proteins in tumor xenografts were consistent with those observed in vitro, supporting the regulatory role of the USP32–MAPK12 axis in promoting ferroptosis resistance in LUAD. Immunohistochemical staining for Ki67, a nuclear protein indicative of proliferative activity, revealed that *MAPK12* upregulation significantly increased the proliferative index of tumor cells. This observation aligns with clinical evidence showing that elevated MAPK12 expression in LUAD patients correlates with reduced overall survival. Conversely, *USP32* suppression resulted in increased cell accumulation in the G_0_ phase of the cell cycle, suggesting that the USP32–MAPK12 axis contributes to cell cycle progression and tumor growth by promoting exit from quiescence.

Similarly, we co-transfected cells with *shMAPK12* and/or *USP32* to knock down *MAPK12* and/or overexpress *USP32* (Supplementary Fig. 3B). Levels of ferroptosis-associated markers—MDA, GSH, and Fe^2+^—were evaluated. *MAPK12* silencing resulted in reduced GSH and elevated MDA and Fe^2+^ levels, whereas *USP32* overexpression produced the opposite effect, enhancing GSH while decreasing MDA and Fe^2+^ accumulation. These findings confirmed that the USP32-MAPK12 axis is negatively correlated with ferroptosis (Supplementary Fig. 3C). Colony formation and Transwell assays further demonstrated that the USP32-MAPK12 axis is positively correlated with cancer cell proliferation and migration (Supplementary Fig. 3D).

### Combined deubiquitination inhibition and MAPK12 knockdown suppress LUAD progression

**Fig. 7:**
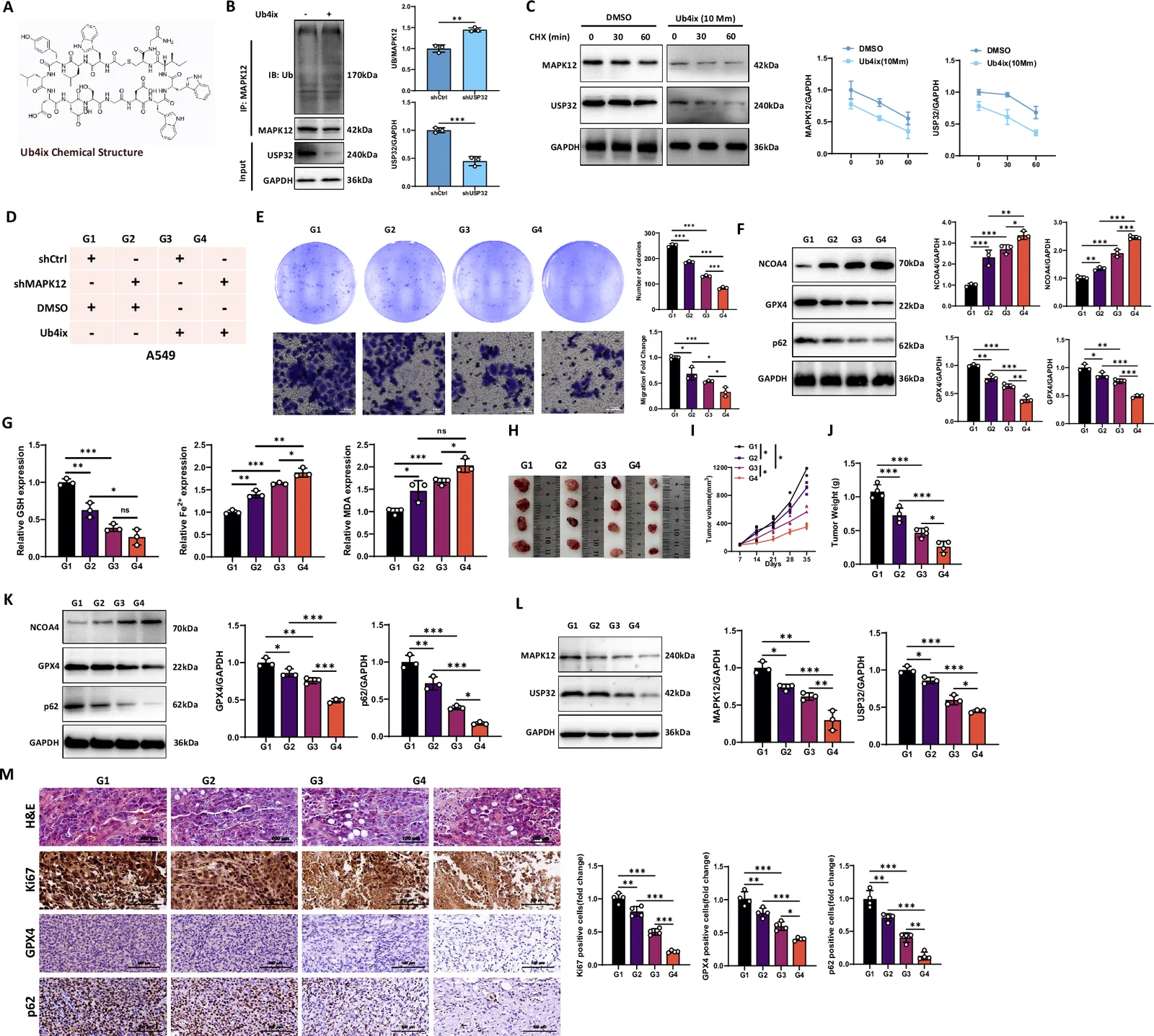
Combined inhibition of USP32 and MAPK12 suppresses LUAD progression. **A** The structure of Ub4ix, a DUB inhibitor used to inhibit USP32 function. **B** In vitro ubiquitination assays demonstrate that Ub4ix effectively prevents deubiquitination of ubiquitin chains, *n* = 3. **C** WB analysis shows that Ub4ix treatment accelerates MAPK12 degradation in A549 cells treated with CHX, *n* = 3. **D** MAPK12 knockdown combined with Ub4ix treatment in A549 cells. **E** Colony formation and Transwell assays indicate that combined treatment markedly decreases proliferation and migration of A549 cells, *n* = 3. **F** WB analysis reveals decreased levels of NCOA4, GPX4, and p62 proteins under combined treatment, *n* = 3. **G** Biochemical assays show enhanced ferroptosis, evidenced by altered levels of GSH, Fe^2+^, and MDA, *n* = 3. **H** Representative images of tumors formed by A549 cells in nude mice after 35 days demonstrate the efficacy of combined treatment in vivo, *n* = 4. **I** Tumor growth curves indicate that combined inhibition significantly slows tumor progression, *n* = 4. **J** Average tumor weights confirm the suppressive effect of the combined treatment, *n* = 4. **K** WB analysis of tumor tissues shows reduced expression of NCOA4, GPX4, and p62, *n* = 3. **L** WB analysis of tumor tissues shows reduced expression of MAPK12 and USP32, *n* = 3. **M** Representative histological examination of tumor tissues using H&E staining, along with immunohistochemical staining for Ki67, GPX4, and p62, *n* = 4. ^*^P < 0.05; ^**^P < 0.01; ^***^P < 0.001.

Ub4ix is a DUB inhibitor that prevents the DUB-mediated cleavage of K48-linked polyubiquitin chains^44,45^ (Fig. 7A). To evaluate the impact of Ub4ix on MAPK12, we first performed in vitro ubiquitination assays. Ub4ix treatment significantly increased the ubiquitination levels of MAPK12 (Fig. 7B), indicating its effective inhibition of deubiquitination processes. To evaluate how Ub4ix influences MAPK12 protein stability, cells were co-treated with Ub4ix and cycloheximide (CHX) to inhibit de novo protein synthesis and track the degradation kinetics of MAPK12 over time. The results showed that Ub4ix markedly accelerated MAPK12 degradation through the ubiquitin–proteasome system (Fig. 7C).

Interestingly, although the primary pharmacological mechanism of Ub4ix is known to involve blocking USP32’s recognition of K48-linked ubiquitin dimers, our data revealed an additional effect: Ub4ix exposure also resulted in decreased levels of endogenous USP32 protein (Fig. 7B, C).

After confirming that Ub4ix regulates both MAPK12 and USP32, we further examined its functional effects in A549 cells by conducting individual and combined treatments with shMAPK12 and Ub4ix (Fig. 7D). Colony formation and Transwell migration assays revealed that Ub4ix alone more effectively inhibited LUAD cell proliferation and migration than MAPK12 knockdown alone. Importantly, co-treatment with Ub4ix and *MAPK12* knockdown synergistically suppressed tumor cell proliferation and migratory capacity (Fig. 7E).

At the molecular level, both *MAPK12* knockdown and Ub4ix treatment independently increased the expression of NCOA4, a key regulator of ferritinophagy, while reducing the levels of the antioxidant enzyme GPX4 and the autophagy substrate p62 (Fig. 7F). These changes were accompanied by GSH depletion, elevated Fe^2+^ release, and increased accumulation of the lipid peroxidation marker MDA, indicative of activated ferroptotic signaling (Fig. 7G).

To further validate the in vitro findings in vivo, we established stable *MAPK12* knockdown A549 cell lines via lentiviral transduction and subcutaneously implanted them into immunodeficient mice (Fig. 7H). Beginning on day 7 after tumor implantation, one cohort of mice was administered daily intraperitoneal injections of Ub4ix. Combined treatment with *MAPK12* knockdown and Ub4ix markedly suppressed tumor progression, yielding the lowest tumor volumes and weights across all experimental groups (Fig. 7I, J).

WB analysis of tumor lysates demonstrated elevated NCOA4 expression along with reduced levels of GPX4 and p62 following combination treatment, mirroring in vitro findings and suggesting concurrent activation of ferroptosis and autophagy in vivo (Fig. 7K). In parallel, a marked decrease in MAPK12 and USP32 expression was also observed (Fig. 7L).

H&E staining of tumor sections from untreated xenografts revealed a dense and homogeneous tissue structure (Fig. 7M). In contrast, *MAPK12* knockdown induced small vacuolar structures, while Ub4ix treatment markedly increased vacuolization within the tumor mass—morphological changes associated with cell death and ferroptotic activity. Furthermore, immunohistochemical staining for the proliferation marker Ki-67 confirmed that both *MAPK12* knockdown and Ub4ix treatment individually suppressed tumor cell proliferation.

## Discussion

This study revealed that the USP32–MAPK12 signaling axis plays an essential role in driving LUAD progression by concurrently inhibiting autophagy and ferroptosis, two fundamental forms of regulated cell death. Our findings indicate that USP32 stabilizes MAPK12 through deubiquitination, which in turn enhances LUAD cell survival by inhibiting both autophagy and ferroptosis, allowing tumor cells to evade programmed cell death. Targeting this axis provides a promising therapeutic strategy for LUAD treatment, as inhibition of USP32 or MAPK12 significantly reduced tumor growth and enhanced ferroptosis-related cell death.

The role of ubiquitination and deubiquitination in cancer progression is well-established, with numerous studies highlighting the importance of this post-translational modification in regulating protein stability and function^30,46^. Aberrant ubiquitination often leads to the degradation of tumor suppressors, while deubiquitination stabilizes oncogenic proteins, thus supporting cancer cell survival and proliferation. In our study, we confirmed that USP32 selectively removes K48-linked ubiquitin chains from MAPK12, preventing its proteasomal degradation and thus sustaining its oncogenic activity in LUAD cells. This observation is consistent with prior studies highlighting the role of DUBs in regulating critical oncogenic signaling pathways across multiple cancer types^47–49^.

Ferroptosis has emerged as a potential target for cancer therapy^50,51^. In our research, MAPK12 was identified as a critical inhibitor of ferroptosis in LUAD cells, as its downregulation triggered lipid peroxidation and increased intracellular Fe^2+^ levels, hallmarks of ferroptotic cell death. Inhibition of the USP32-MAPK12 axis restored the sensitivity of LUAD cells to ferroptosis, suggesting that MAPK12’s protective role against ferroptosis is pivotal for tumor cell survival. Notably, we observed that suppression of ferroptosis was accompanied by a decrease in autophagy, another survival mechanism frequently upregulated in cancer cells under stress conditions.

Autophagy, which recycles damaged cellular components, has a complex relationship with ferroptosis. While autophagy can promote cell survival by degrading damaged organelles, excessive autophagy can lead to autophagic cell death^33^. Our study revealed that MAPK12 inhibits autophagy by preventing the degradation of p62 and the activation of LC3B, both of which are critical markers of autophagic flux. When the USP32-MAPK12 axis was inhibited, autophagy was significantly upregulated, resulting in enhanced clearance of damaged mitochondria and promoting ferroptosis. This suggests that autophagy and ferroptosis are closely linked, with MAPK12 serving as a central regulator of this interplay in LUAD cells. While our study utilized Ub4ix as a tool to inhibit the USP32-mediated deubiquitination of MAPK12, it is important to note that Ub4ix is not entirely specific to USP32. As previously reported, Ub4ix, a cyclic peptide designed to block the cleavage of K48-linked polyubiquitin chains, also affects other DUBs. This specificity issue is a limitation of our study and should be considered in future research. Future studies employing catalytic mutants of USP32 or more selective inhibitors, such as those that target DUBs via specific binding assays (e.g., bio-layer interferometry or DUB activity profiling), will be crucial to further dissect the substrate specificity of USP32 and confirm the precise roles of other K48-linked DUBs in LUAD progression.

Building upon our findings that MAPK12 overexpression suppresses both autophagy and ferroptosis in LUAD cells, we propose several convergent mechanisms underlying this dual inhibitory effect. MAPK12, as a member of the p38 MAPK family, may modulate these pathways through phosphorylation-dependent regulation of stress-response and metabolic effectors. Specifically, MAPK12 may inhibit autophagy initiation by promoting mTOR activity or directly phosphorylating ULK1 at inhibitory sites (e.g., Ser757), thereby disrupting the ULK1-AMPK interaction essential for autophagosome formation^40,52^. Our data support this model, as MAPK12 knockdown increased LC3-II accumulation and reduced p62 levels, indicating enhanced autophagic flux. Furthermore, overexpression of MAPK12 led to accumulation of p62 and suppression of LC3 lipidation, consistent with autophagy inhibition.

In ferroptosis regulation, MAPK12 appears to modulate several core effectors. Its overexpression led to increased GPX4 and FTH1 expression, while downregulating ACSL4 and NCOA4—key drivers of lipid peroxidation and ferritinophagy, respectively. These effects likely converge on the NRF2 pathway, as MAPK family members have been shown to stabilize NRF2, enhancing antioxidant gene expression^53,54^. Additionally, MAPK12 may suppress mitochondrial iron release and lipid ROS formation, as evidenced by decreased Fe^2+^ and MDA levels in MAPK12-overexpressing cells^55^. These data collectively indicate that MAPK12 orchestrates a multifaceted protective program against ferroptotic stress.

Importantly, these mechanistic inferences align with our experimental results but remain consistent with prior observations on p38 MAPK family members such as p38α. While direct phosphorylation targets of MAPK12 require further phosphoproteomic validation, the observed suppression of autophagy and ferroptosis markers supports a model wherein MAPK12 functions as a central rheostat coordinating cell survival under stress. These findings position MAPK12 not only as a downstream effector of USP32-mediated oncogenic signaling but also as a critical node linking redox, metabolic, and autophagic networks in LUAD progression.

Our findings corroborate earlier research that identified MAPK12 as a pro-survival kinase in various cancers^25–27,56^. However, unlike previous studies that focused on its role in stress response and proliferation, our work highlights its involvement in ferroptosis and autophagy suppression, revealing a mechanism of tumor resistance. These findings align with previous reports on the p38 MAPK family, which suggest that different isoforms may have opposing roles in cell survival and death, depending on the cellular context^57–60^. Our study adds to this body of knowledge by establishing MAPK12 as a critical player in ferroptosis resistance in LUAD, mediated by its stabilization through USP32.

One of the notable contradictions in the literature is the dual role of autophagy in cancer, where it can either promote tumor survival or induce cell death. Our data support the notion that autophagy serves a pro-survival function in LUAD cells when regulated by MAPK12, as its inhibition through MAPK12 downregulation leads to enhanced ferroptosis. This points to a context-dependent role for autophagy, where its modulation could be exploited for therapeutic purposes. Additional research is required to elucidate the precise molecular crosstalk between autophagy and ferroptosis, as well as to determine how these pathways can be selectively modulated across distinct cancer types.

Future research should focus on identifying specific inhibitors of USP32 and MAPK12 that can be developed into effective therapeutic agents for LUAD. Additionally, exploring the role of the USP32-MAPK12 axis in other cancers could provide broader insights into its potential as a universal target for malignancies that exhibit resistance to ferroptosis. Understanding how this axis interacts with other cell death pathways, such as apoptosis, could also reveal strategies for overcoming tumor resistance to conventional therapies.

In conclusion, our study highlights the USP32-MAPK12 axis as a therapeutic target in LUAD. By stabilizing MAPK12, USP32 promotes tumor survival by suppressing both autophagy and ferroptosis. Inhibition of this axis restores ferroptosis and autophagy, leading to significant tumor suppression.

## Methods

### Clinical sample collection

Clinical specimens were obtained from patients (Han Chinese ethnicity in China) at Sir Run Run Shaw Hospital with informed consent. All ethical regulations relevant to human research participants were followed. The clinical samples were obtained from the hospital’s pathological archive and were retrospectively included in the study for patients with surgically resected lung adenocarcinoma. All procedures involving sample collection, handling, analysis, and disposal strictly followed protocols approved by the Ethics Committee Approval Letter of Sir Run Run Shaw Hospital, Zhejiang University School of Medicine (*Approval No. 2025-2550-01*), ensuring full adherence to ethical and legal standards. All patient data have been anonymized, and no individually identifiable information is presented in this study. Gender information was not collected, as the study focused on tissue-level molecular analysis. The use of these anonymized archival samples was approved by the institutional ethics committee with a waiver for individual consent for this specific research use. Individual-level sex data are provided in a disaggregated manner in the Supplementary Data file, with patient identifiers removed to protect confidentiality.

### Cell culture and reagents

LUAD cell lines A549, PC-9, NCI-H1299, NCI-H1703, and the normal human bronchial epithelial cell line 16HBE were obtained from the National Collection of Authenticated Cell Cultures (Shanghai, China). Cells were maintained in RPMI-1640 medium (Gibco) containing 10% fetal bovine serum (FBS), 100 units/mL penicillin, and 100 µg/mL streptomycin, and incubated at 37 °C in a humidified incubator with 5% CO₂. For functional assays, cells were treated with ferroptosis inducers (Erastin, Sigma), autophagy inhibitors (chloroquine, Sigma), and proteasome inhibitors (MG132, Sigma) as indicated in specific experiments.

### Western blotting (WB)

Cellular proteins were extracted using RIPA lysis buffer containing protease and phosphatase inhibitor cocktails (Roche). Protein levels were quantified with the BCA Protein Assay Kit (Thermo Fisher). Equivalent protein quantities were subjected to SDS-PAGE, followed by transfer onto PVDF membranes. After blocking with 5% non-fat milk, membranes were incubated overnight with primary antibodies targeting USP32 (1:1000, #47247, Cell Signaling Technology), MAPK12 (1:1000, #2307, Cell Signaling Technology), p62 (1:1000, #88588, Cell Signaling Technology), LC3B (1:1000, #2775, Cell Signaling Technology), GPX4 (1:1000, #59735, Cell Signaling Technology), ACSL4 (1:1000, #38493, Cell Signaling Technology), FTH1 (1:1000, #3998, Cell Signaling Technology), and GAPDH (1:1000, #2118, Cell Signaling Technology). After incubation with HRP-conjugated secondary antibodies, protein bands were detected using enhanced chemiluminescence (ECL, Thermo Fisher). The intensity of each band was measured with ImageJ software.

### Quantitative real-time PCR (qPCR) analysis

qPCR was used to quantify *MAPK12* mRNA expression. Total RNA was isolated with TRIzol reagent (Invitrogen), and complementary DNA (cDNA) was generated using the RevertAid First Strand cDNA Synthesis Kit (Thermo Fisher). qPCR was conducted with SYBR Green PCR Master Mix (Applied Biosystems) on a LightCycler 480 system (Roche). Primer sequences were as follows:

*MAPK12* forward: 5′-GACCTGCAGCCCGTGGGCTC-3′ *MAPK12* reverse: 5′-TGGACTTGAAGATCTCAGAGCAC-3′ *GAPDH* served as the internal control gene, and relative mRNA expression was determined using the 2^−ΔΔCt^ calculation method. All samples were assessed in three independent replicates.

### Colony formation assays

Cells were plated in 6-well culture dishes at a density of 500 cells per well and incubated for 14 days to allow colony development. Formed colonies were fixed using 4% paraformaldehyde, stained with crystal violet, and subsequently counted under a light microscope.

### Transwell migration assay

Cells were loaded into the upper compartment of Transwell inserts (8-µm pore size, Corning) containing serum-free RPMI-1640 medium, while the lower wells were filled with RPMI-1640 supplemented with 10% fetal bovine serum to serve as a chemoattractant. After 24 h of incubation, cells that traversed to the underside of the membrane were fixed in methanol, stained with crystal violet, and enumerated under a microscope.

### MitoTracker staining

MitoTracker Red CMXRos (Invitrogen) was used to assess mitochondrial health and membrane potential. Cells were rinsed twice with PBS, then exposed to 100 nM MitoTracker Red diluted in serum-free medium at 37 °C for 30 min. After an additional PBS wash, fluorescence imaging was performed immediately under a fluorescence microscope. MitoTracker selectively accumulates in active mitochondria, with red fluorescence proportional to mitochondrial membrane potential.

### Transmission electron microscopy (TEM)

TEM was employed to examine autophagosome formation and mitochondrial ultrastructure. Cells were initially fixed with 2.5% glutaraldehyde at 4 °C overnight, rinsed with PBS, and subsequently post-fixed in 1% osmium tetroxide for 1 h. Specimens underwent dehydration through a graded ethanol series ranging from 50% to 100%, then were embedded in epoxy resin. Ultrathin sections (70–90 nm) were cut and mounted onto copper grids. The sections were stained with 2% uranyl acetate for 10 min, followed by 0.4% lead citrate for 5 min. Imaging was performed using a JEOL JEM-1200EX transmission electron microscope, and morphological features of autophagosomes and mitochondria were assessed.

### Plasmid construction and transfection

Full-length human *USP32* and *MAPK12* cDNAs were cloned into the pcDNA3.1 expression vector with Myc and Flag tags. Site-directed mutagenesis was performed to generate a catalytically inactive *USP32* mutant (*USP32*^*C743A*^) using the QuikChange Lightning Site-Directed Mutagenesis Kit (Agilent). Small hairpin RNAs (shRNAs) targeting *USP32* and *MAPK12* were cloned into the pLKO.1 vector for knockdown experiments. Transient transfections were performed with Lipofectamine 2000 (Invitrogen) according to the manufacturer’s protocol.

### Co-immunoprecipitation (Co-IP)

For Co-IP, cells were lysed in IP lysis buffer (Thermo Fisher) and incubated with anti-MAPK12 or anti-USP32 antibodies at 4 °C overnight. Antigen-antibody complexes were pulled down using protein A/G agarose beads (Thermo Fisher), washed with lysis buffer, and subjected to WB analysis. Input lysates were used to confirm target protein expression.

### Ubiquitination assay

Cells were transfected with HA-ubiquitin (Ub WT, Ub Lys48-only, or Ub Lys63-only constructs) and USP32 and MAPK12 expression vectors. After 48 h, cells were treated with MG132 (10 µM) for 6 h before harvesting. Cell lysates were immunoprecipitated with anti-MAPK12 antibodies, and ubiquitinated MAPK12 was detected by WB using an anti-HA antibody.

### Ferroptosis and autophagy assays

Cells were treated with Erastin (10 µM) for 24 h to analyze ferroptosis. Intracellular ferrous iron (Fe^2+^) levels, malondialdehyde (MDA), and reduced glutathione (GSH) were measured using commercial kits (Beyotime). Lipid peroxidation was assessed using the BODIPY C11 probe (Thermo Fisher). For autophagy assays, cells were transfected with mRFP-GFP-LC3 plasmids, and autophagic flux was monitored by the formation of LC3-positive puncta under a fluorescence microscope. TEM was used to visualize autophagosomes.

### Cell viability and CFDA staining

Cell viability was determined using the Cell Counting Kit-8 (CCK-8, Dojindo). CFDA staining was performed by incubating cells in serum-free medium with 5 µM CFDA at 37 °C for 30 min. Cells were washed and imaged under a fluorescence microscope. CFDA is hydrolyzed in live cells, emitting green fluorescence proportional to viability.

### Stable cell line construction and lentiviral packaging

Stable cell lines were established via lentiviral infection. HEK293T cells were co-transfected with pLKO.1-based shRNA vectors or overexpression plasmids along with packaging constructs (pMD2.G and psPAX2). After 48 h, viral-containing supernatants were harvested and filtered. LUAD target cells were then transduced with the viral particles in the presence of 8 µg/mL polybrene to enhance infection efficiency. After 48 h, cells were selected using 2 µg/mL puromycin to establish stable lines. Lentiviral transduction efficiency was confirmed by qPCR and WB analysis.

### Xenograft tumor model

Male BALB/c nude mice (6–8 weeks old, Cat. SM-014) were purchased from Shanghai Model Organisms Center, Inc. and maintained under specific pathogen-free conditions. All animal procedures were approved by the Institutional Animal Care and Ethics Committee Approval Letter of Sir Run Run Shaw Hospital, Zhejiang University School of Medicine (*Approval No. ZJCLA-IACUC-20010872*). We have complied with all relevant ethical regulations for animal use. NCI-H1299 cells (5 × 10⁶) suspended in 100 μL of PBS were subcutaneously injected into the right flank of each mouse. Two independent xenograft experiments were conducted, each comprising four experimental groups (*n* = 4 per group). In the first batch, mice were randomly assigned to the designated groups and subcutaneously injected with NCI-H1299 cells genetically modified to overexpress or knock down specific target genes, according to each experimental condition: a control group (vector only), a MAPK12 overexpression group, a group with simultaneous MAPK12 overexpression and USP32 knockdown, and a USP32 knockdown group. In the second batch, mice were similarly assigned to a control group, a MAPK12 knockdown (shMAPK12) group, a group receiving both shMAPK12 and Ub4ix treatment, and a Ub4ix treatment group.

Ub4ix was administered intraperitoneally at a dose of 20 mg/kg, formulated in vehicle solution (10% DMSO, 40% PEG300, 5% Tween-80, and 45% saline), every other day starting on day 7 post-inoculation, when tumor volumes reached approximately 100 mm³. Mice were monitored every 7 days, and tumor volume was calculated using the formula: *V* = *(length × width²)/2*. Mice were euthanized when tumor burden approached or reached the maximal permitted volume (1500 mm³), or at the predefined experimental endpoint (day 35), and tumors were collected for histological and molecular analysis. The maximum permitted tumor volume (1500 mm³) was approved by the Institutional Animal Care and Ethics Committee of Sir Run Run Shaw Hospital, Zhejiang University School of Medicine. All mice in this study were monitored regularly, and no tumor exceeded the approved maximal tumor burden throughout the experimental period.

The sample size of 4 mice per group was based on previous studies and the need to detect meaningful differences in tumor growth and biomarkers. This number provides adequate statistical power for detecting treatment effects while minimizing animal use. Animals were included in the experiment if they were healthy, with no signs of abnormal weight loss or disease prior to the experiment. Mice showing significant health issues during the experiment (e.g., severe weight loss, lethargy, or infection) were excluded from analysis. Data points with incomplete measurements or extreme outliers were excluded from the statistical analysis. Randomization was used to allocate experimental units to control and treatment groups. Mice were randomly assigned to each group using a random number generator, ensuring unbiased allocation to the control and experimental groups. To minimize potential confounders, the order of treatments and measurements was randomized, and animals were housed in different cages to avoid any environmental bias. All experimental units were treated and measured under the same conditions to ensure consistency. No specific confounders were left uncontrolled. Experimenters who performed the treatments were blinded to the group assignments. During the conduct of the experiment, the researchers administering treatments and measurements were not aware of the group allocations. Outcome assessment was performed by researchers blinded to the group allocations. For data analysis, the experimenters were not aware of the group allocations, ensuring unbiased analysis. The primary outcome measure was tumor growth, as assessed by tumor volume and weight in the xenograft models. This measure was used to determine the sample size, with the goal of detecting significant differences in tumor progression between the control and treatment groups.

### Histology and immunohistochemistry (IHC)

Tumor specimens were fixed in 4% paraformaldehyde, embedded in paraffin, and sectioned for hematoxylin and eosin (H&E) staining. For immunohistochemistry (IHC), tissue sections were incubated overnight at 4 °C with the following primary antibodies: anti-Ki67 (1:1600, #9449, Cell Signaling Technology), anti-GPX4 (1:1600, #59735, Cell Signaling Technology), and anti-p62 (1:800, #88588, Cell Signaling Technology). Detection was performed using an HRP-conjugated secondary antibody. The proliferative index was calculated as the percentage of Ki67-positive cells in five randomly selected microscopic fields.

### Kaplan–Meier survival analysis

To assess the prognostic value of USP32 and MAPK12 expression in LUAD, we performed Kaplan–Meier survival analysis using patient data from TCGA-LUAD dataset. The dataset is publicly available at the GDC (Genomic Data Commons) portal with the unique project identifier: TCGA-LUAD (Project ID: TCGA-LUAD). Patients were categorized into four groups based on the expression levels of USP32 and MAPK12. The groups were defined as follows: “high_high”: Patients with high expression of both MAPK12 and USP32.“high_low”: Patients with high expression of MAPK12 and low expression of USP32.“low_high”: Patients with low expression of MAPK12 and high expression of USP32.“low_low”: Patients with low expression of both MAPK12 and USP32.The cutoff for “high” and “low” expression was determined based on the median expression levels of MAPK12 and USP32 in the patient cohort. Overall survival data for each group were obtained and analyzed using the Kaplan–Meier method. The survival curves were compared using the log-rank test to determine statistical significance. The number of patients at risk at each time point is shown below the plot.

### Statistics and reproducibility

Statistical analyses were performed using GraphPad Prism 10 software. Data are presented as the mean ± standard deviation (SD). For comparisons between two groups, Student’s *t*-test was used. For multiple groups, one-way ANOVA followed by Tukey’s post hoc test was applied. A *P*-value < 0.05 was considered statistically significant. For reproducibility, all experiments were performed at least three times independently. Biological replicates (*n*) were defined as independent cell cultures or independent tumor samples/animals. Technical replicates were defined as repeated measurements of the same sample. The sample size (*n*) for each experiment is indicated in the corresponding figure legends. For experiments without statistical analysis (including Western blotting, immunohistochemistry, transmission electron microscopy, immunofluorescence imaging, and colony formation), representative results from at least three independent experiments are shown to ensure reproducibility. Normality was assessed using the Shapiro–Wilk test, and equal variances were evaluated using Levene’s test. If data were not normally distributed or variances were unequal, non-parametric tests or Welch’s *t*-test were used instead.

### Ethics approval and consent to participate

Clinical specimens were obtained from patients at Sir Run Run Shaw Hospital with informed consent. All procedures involving sample collection, handling, analysis, and disposal strictly followed protocols approved by Sir Run Run Shaw Hospital Ethics Committee, ensuring full adherence to ethical and legal standards.

### Reporting summary

Further information on research design is available in the Nature Portfolio Reporting Summary linked to this article.

## Supplementary information

Supplementary Information

Description of Additional Supplementary Materials

Supplementary Data

Reporting Summary

Transparent Peer Review file

## Data Availability

The plasmids generated in this study have been deposited to Addgene with the following accession numbers: pcDNA3.1-Myc-MAPK12 (plasmid #253109), pcDNA3.1-Flag-USP32 (plasmid #253110), pcDNA3.1-USP32^C743A (plasmid #253111), pLKO.1-shRNA-USP32 (plasmid #253112), and pLKO.1-shRNA-MAPK12 (plasmid #253108). Source data for the figures are provided with this paper in the file Supplementary Data. Uncropped blot images are provided in Supplementary information. All other data that support the findings of this study are available from the corresponding author upon reasonable request.
