# Supplementary material for: USP32-mediated stabilization of MAPK12 promotes lung adenocarcinoma progression by inhibiting autophagy-ferroptosis
Source: Commun Biol. 2026 Jul 19;9:1505. doi: 10.1038/s42003-026-10429-z (PMC13751611; doi:10.1038/s42003-026-10429-z)
Supplement: Supplementary file 1 [file 42003_2026_10429_MOESM1_ESM.pdf]

Supplementary Figure legends and Figures

Figure S1: MAPK12 Overexpression Enhances Proliferation and Migration in LUAD Cells

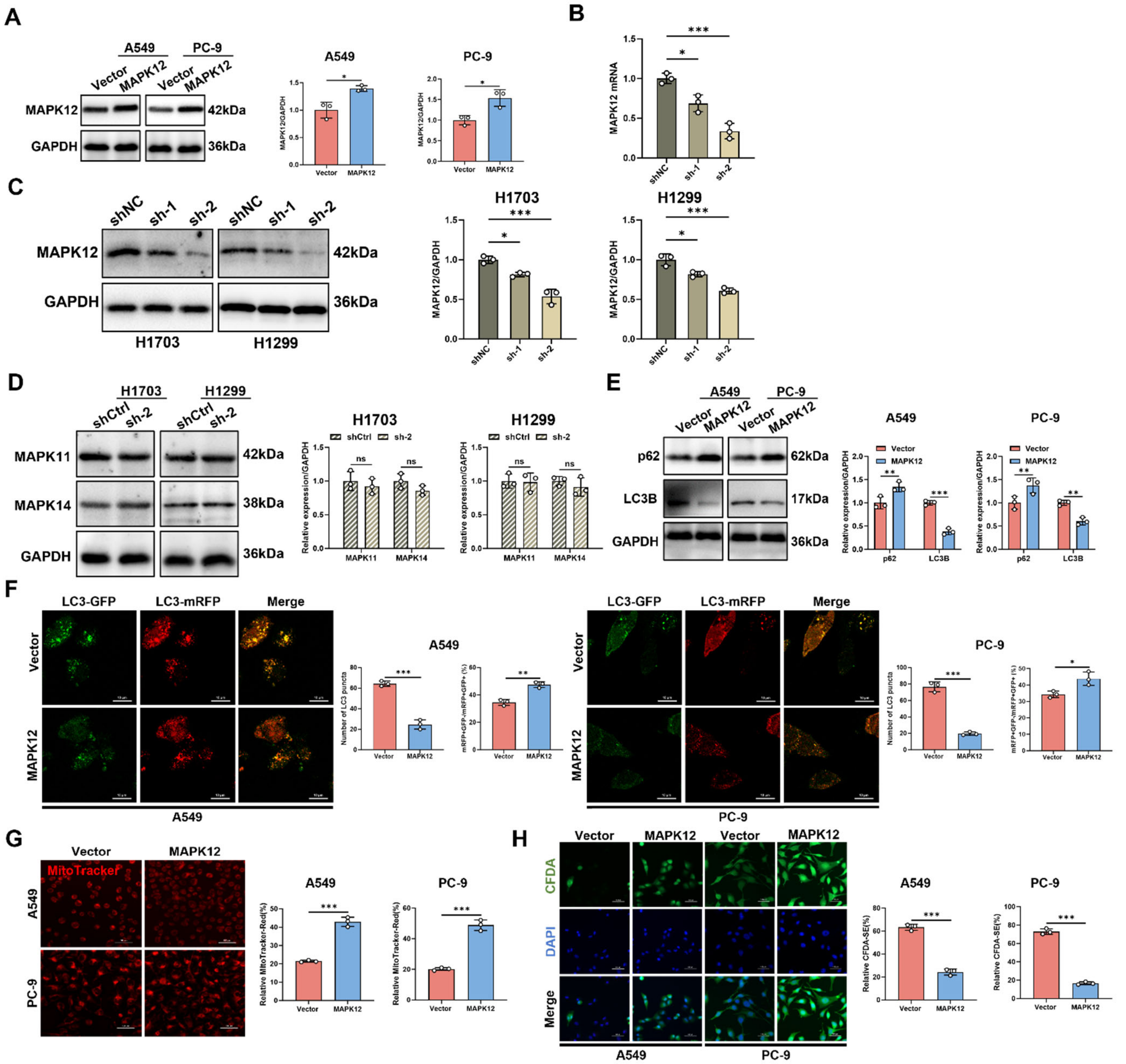

(A) WB analysis and quantification confirm successful overexpression of MAPK12 in A549 and PC-9 cells, n=3.

(B) Quantitative PCR results show the relative knockdown efficiency of MAPK12 mRNA using shMAPK12-1 (sh-1) and shMAPK12-2 (sh-2), n=3.

(C) WB analysis demonstrates reduced MAPK12 protein expression following transfection with sh-1 and sh-2, confirming effective knockdown at the protein level, n=3.

(D) WB analysis shows that MAPK12 knockdown via sh-2 does not affect the protein levels of MAPK11 or MAPK14, indicating target specificity and absence of off-target effects, n=3.

(E) WB analysis shows altered levels of autophagy-related proteins p62 and LC3B after MAPK12 overexpression, n=3.

(F) mRFP-GFP-LC3 dual fluorescence imaging reveals decreased autophagosome and autolysosome formation in A549 and PC-9 cells overexpressing MAPK12, n=3.

(G) MitoTracker staining shows changes in mitochondrial morphology associated with MAPK12 overexpression, n=3.

(H) CFDA and DAPI staining confirm increased cell viability in A549 and PC-9 cells due to MAPK12 overexpression, n=3.

**P* < 0.05; ***P* < 0.01; ****P* < 0.001.

Figure S2: Expression patterns of candidate DUBs and transcriptional correlation between USP32 and MAPK12 in LUAD

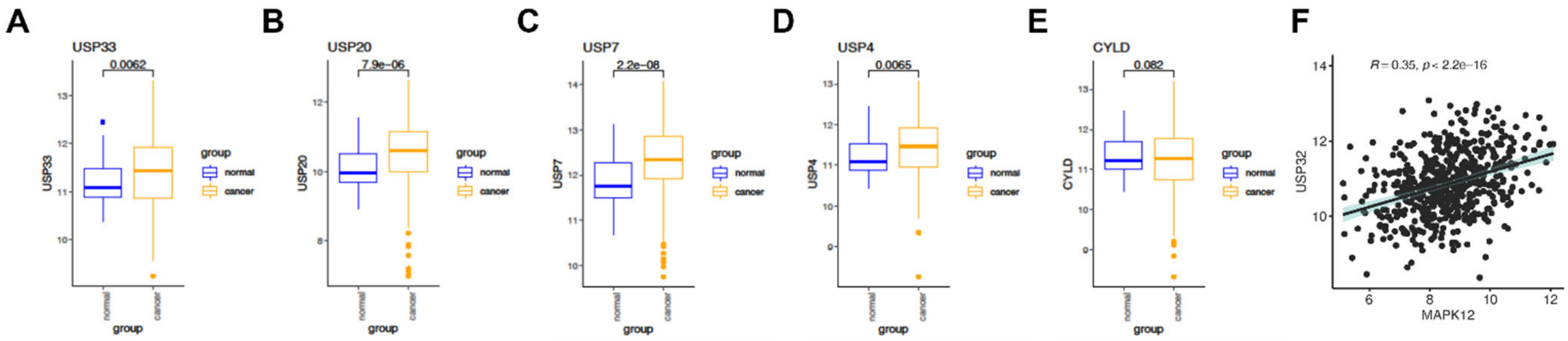

(A) Box plot showing USP33 expression levels in normal individuals and LUAD patients.

(B) Box plot showing USP20 expression levels in normal individuals and LUAD patients.

(C) Box plot showing USP7 expression levels in normal individuals and LUAD patients.

(D) Box plot showing USP4 expression levels in normal individuals and LUAD patients.

(E) Box plot showing CYLD expression levels in normal individuals and LUAD patients.

(F) Pearson correlation analysis reveals a significant positive correlation between USP32 and MAPK12 expression.

Figure S3: Overexpression of USP32 promotes proliferation and migration of LUAD cells.

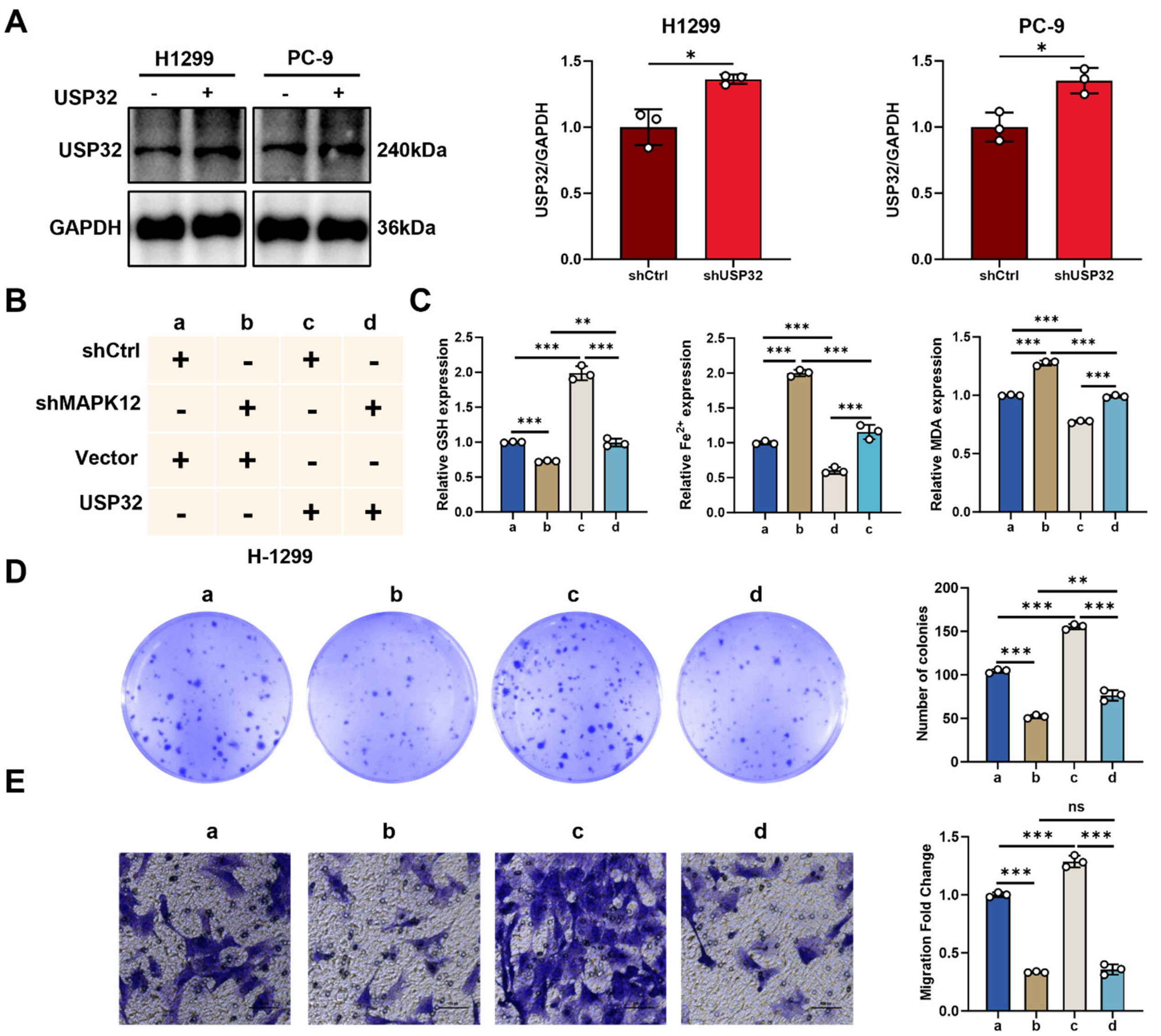

(A) Knockdown of USP32 in NCI-H1299 and A549 cells is validated by WB analysis.

(B) Concurrent MAPK12 knockdown and USP32 overexpression in NCI-H1299 and PC-9 cells are confirmed.

(C) Biochemical assays reveal that MAPK12 knockdown enhances ferroptosis markers (Fe^{2+}, GSH, and MDA) even when USP32 is overexpressed.

(D) Colony formation assays show that MAPK12 knockdown suppresses cell proliferation despite USP32 overexpression.

(E) Transwell assays indicate that MAPK12 knockdown reduces cell migration, counteracting the effects of USP32 overexpression.

$^{*}P < 0.05$; $^{**}P < 0.01$; $^{***}P < 0.001$.

All uncropped blot images

Figure 1 D Case1 GAPDH

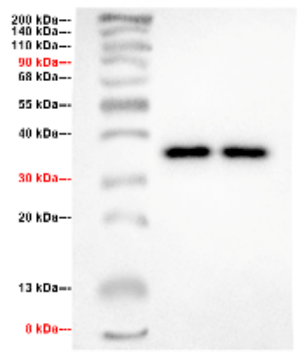

GAPDH（36kDa）.tif

Figure 1 D Case1 MAPK12

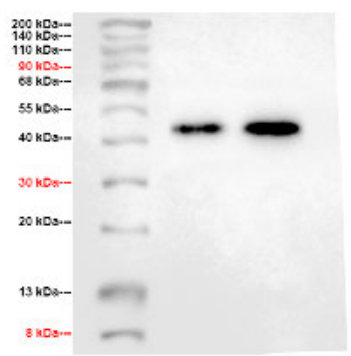

MAPK12（42kDa）.tif

Figure 1 D Case2 GAPDH

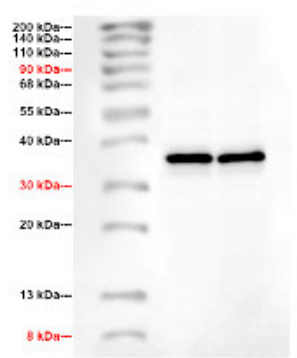

GAPDH（36kDa）.tif

Figure 1 D Case2 MAPK12

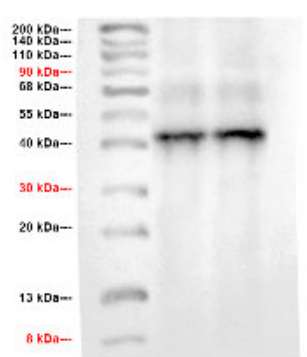

MAPK12（42kDa）.tif

Figure 1 D Case3 GAPDH

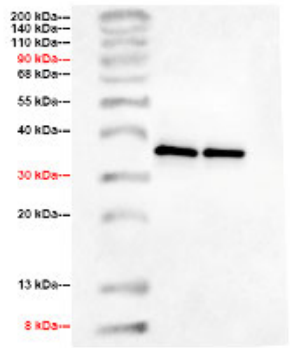

GAPDH（36kDa）.tif

Figure 1 D Case3 MAPK12

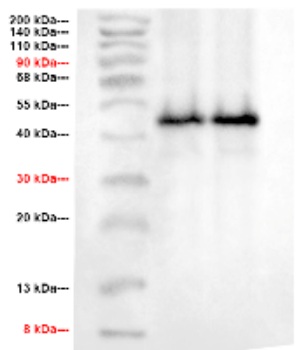

MAPK12（42kDa）.tif

Figure 1 G GAPDH

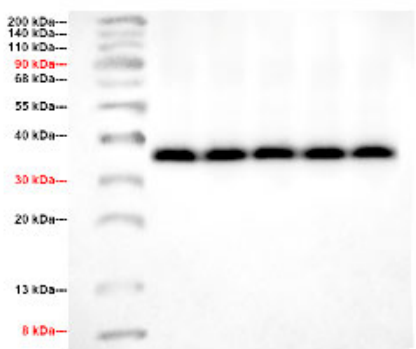

GAPDH（36kDa）.tif

Figure 1 G MAPK12

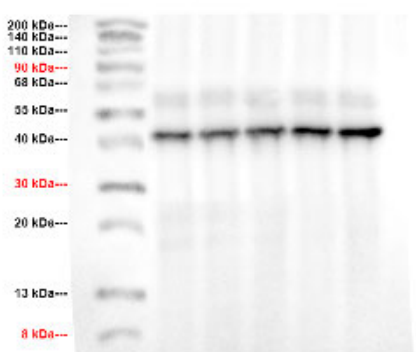

MAPK12（42kDa）.tif

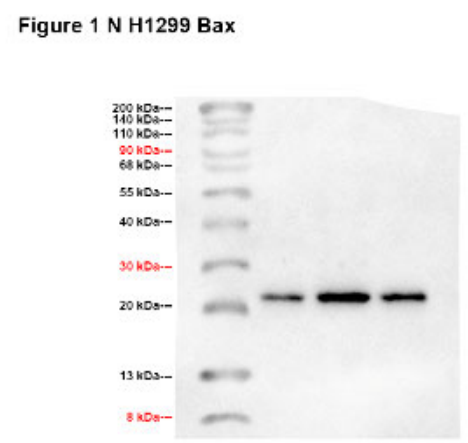

Bax（21kDa）.tif

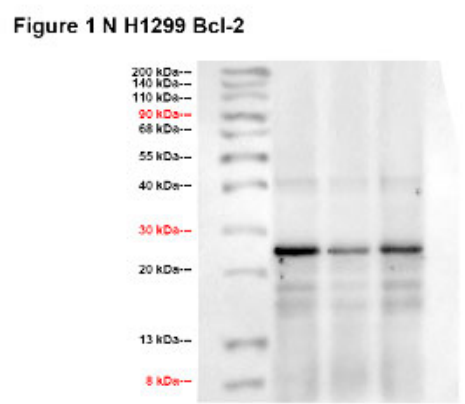

Bcl-2（26kDa）.tif

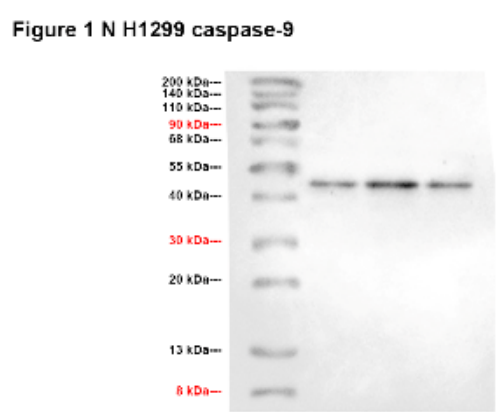

caspase-9（46kDa）.tif

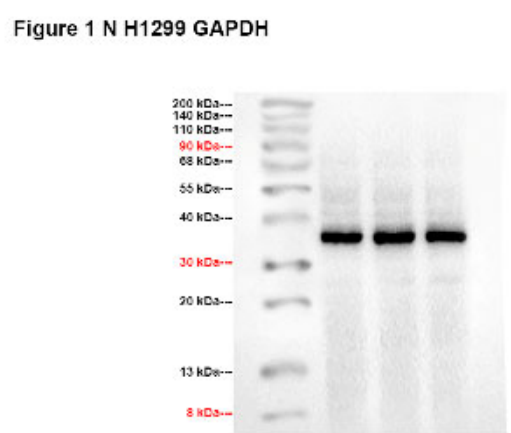

GAPDH（36kDa）.tif

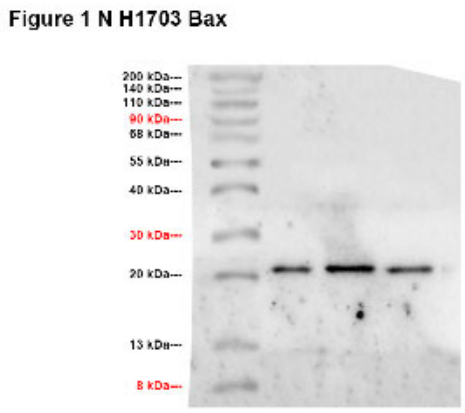

Bax（21kDa）.tif

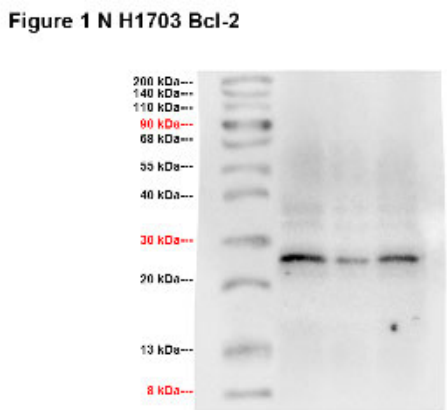

Bcl-2（26kDa）.tif

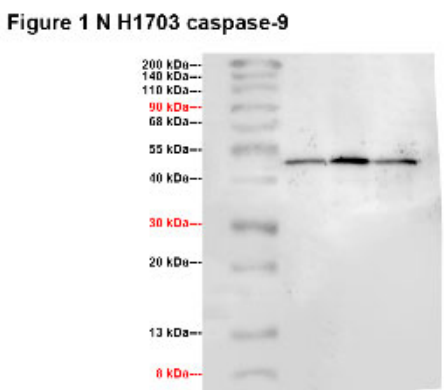

caspase-9（46kDa）.tif

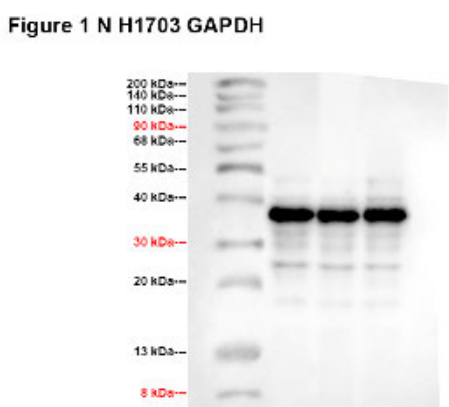

GAPDH（36kDa）.tif

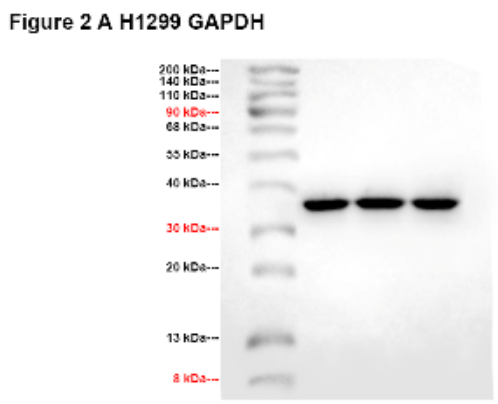

GAPDH（36kDa）.tif

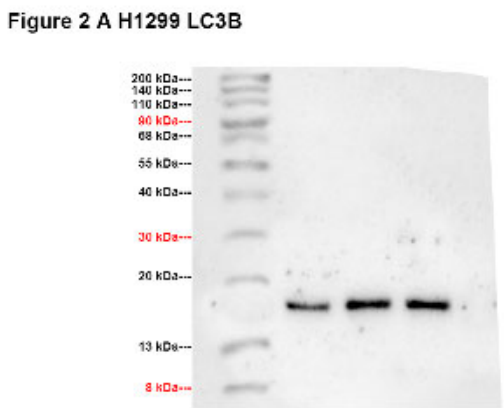

LC3B（17kDa）.tif

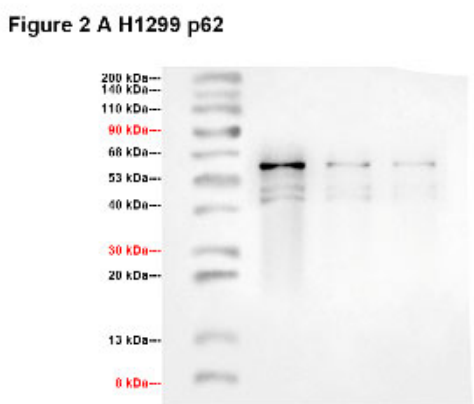

p62（62kDa）.tif

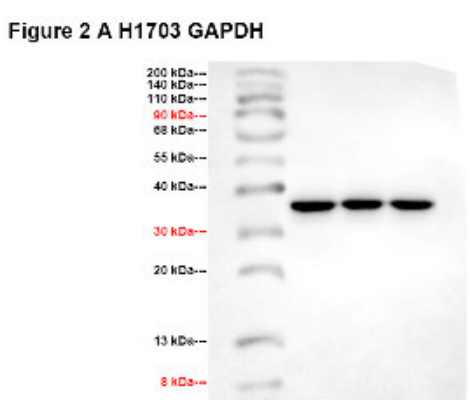

GAPDH（36kDa）.tif

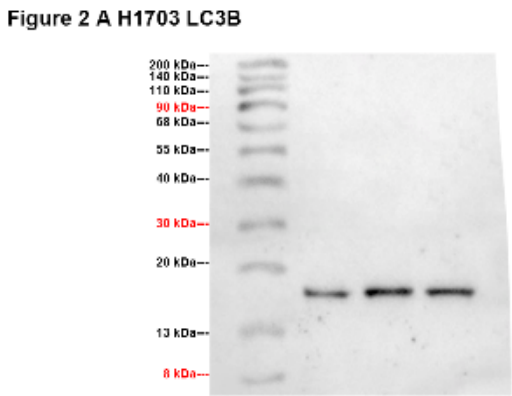

LC3B（17kDa）.tif

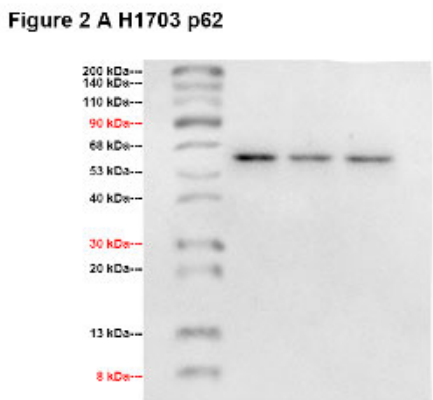

p62（62kDa）.tif

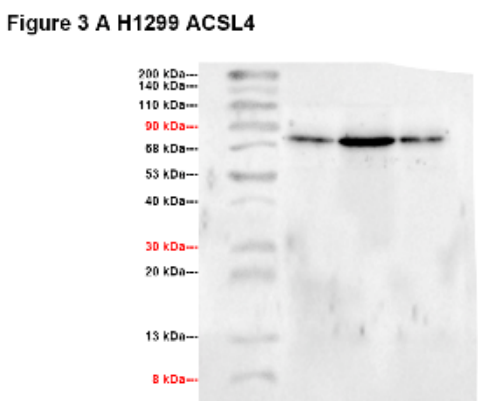

ACSL4（79kDa）.tif

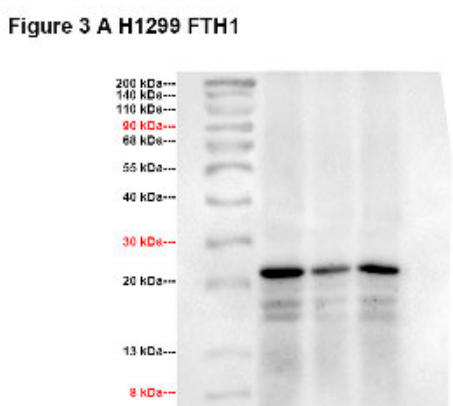

FTH1（21kDa）.tif

Figure 3 A H1299 GAPDH

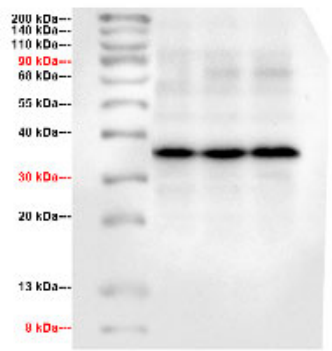

GAPDH（36kDa）.tif

Figure 3 A H1299 GPX4

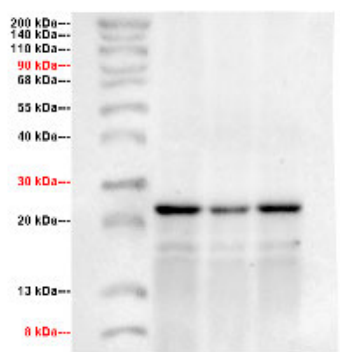

GPX4（22kDa）.tif

Figure 3 A H1299 NCOA4

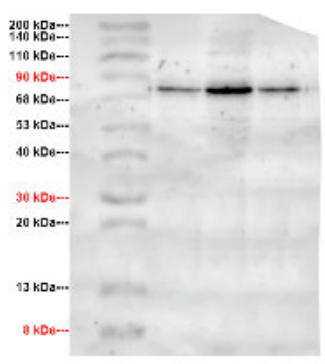

NCOA4（70kDa）.tif

Figure 3 A H1703 ACSL4

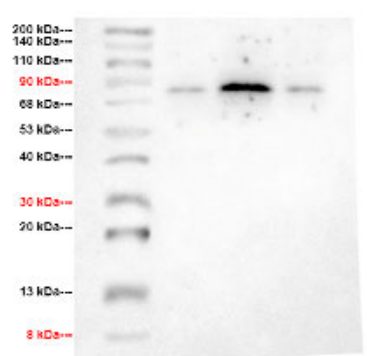

ACSL4（79kDa）.tif

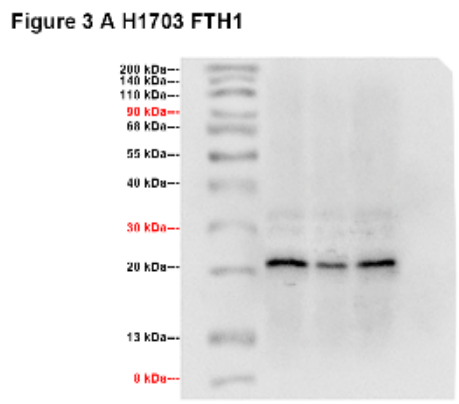

FTH1（21kDa）.tif

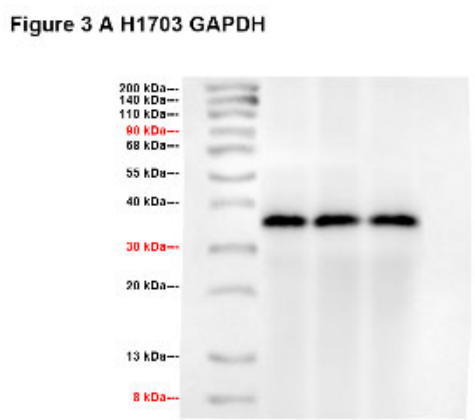

GAPDH（36kDa）.tif

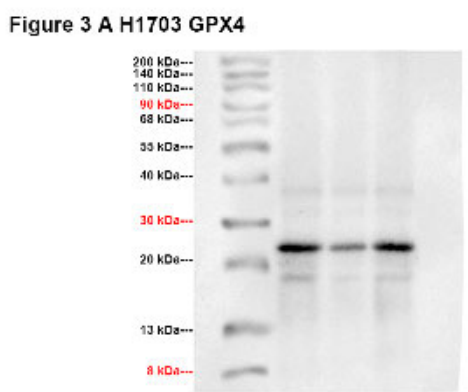

GPX4（22kDa）.tif

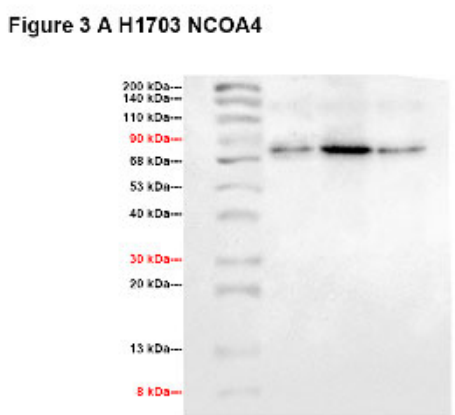

NCOA4（70kDa）.tif

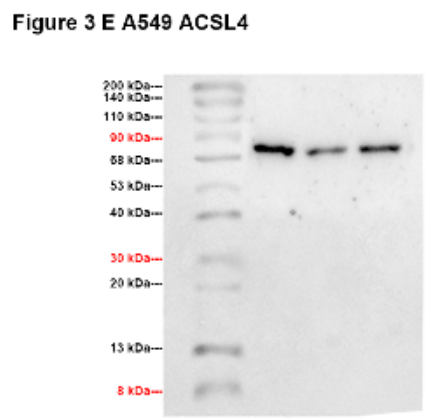

ACSL4（79kDa）.tif

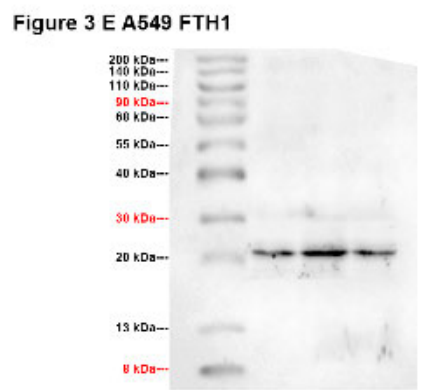

FTH1（21kDa）.tif

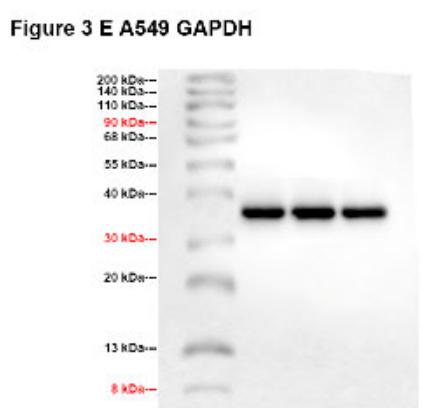

GAPDH（36kDa）.tif

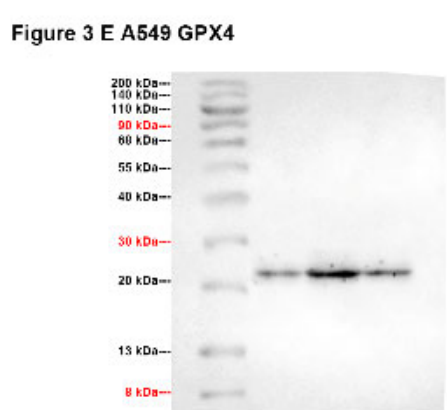

GPX4（22kDa）.tif

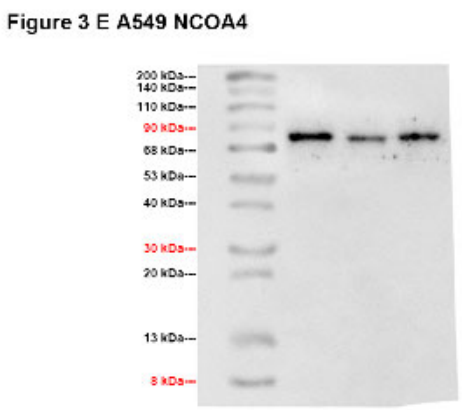

NCOA4（70kDa）.tif

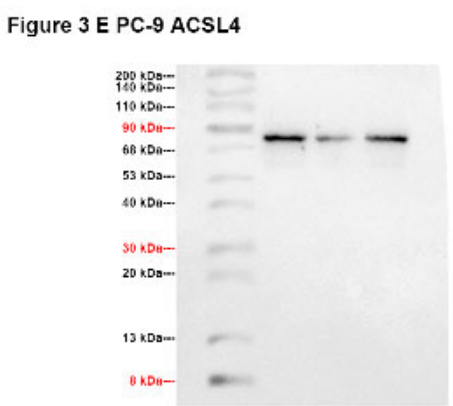

ACSL4（79kDa）.tif

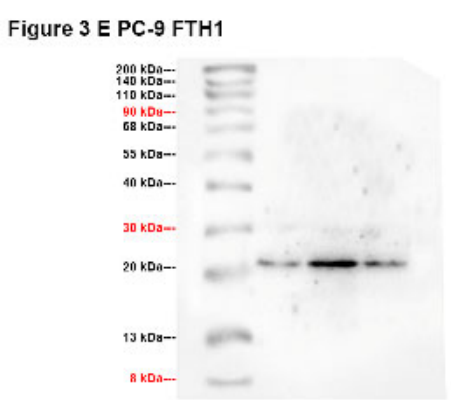

FTH1（21kDa）.tif

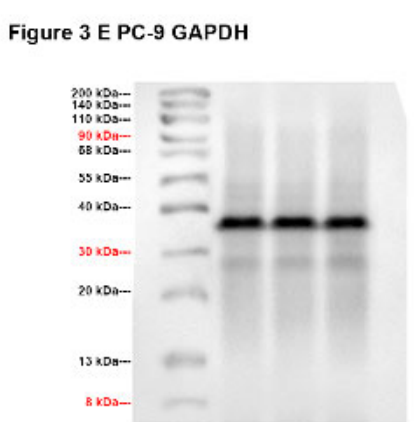

GAPDH（36kDa）.tif

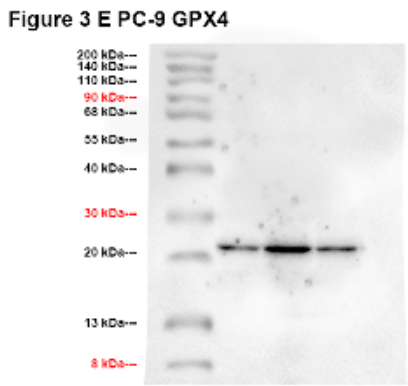

GPX4（22kDa）.tif

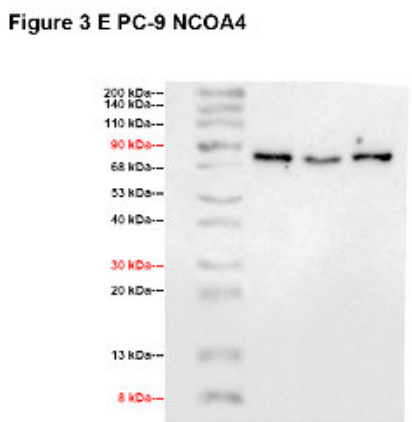

NCOA4（70kDa）.tif

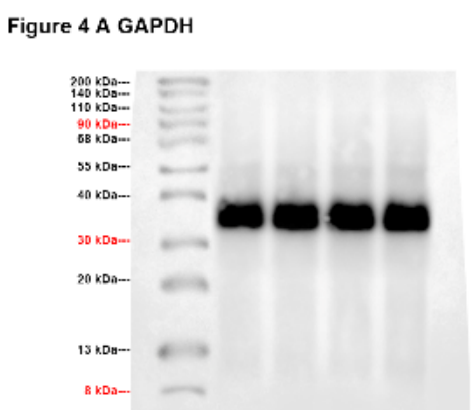

GAPDH（36kDa）.tif

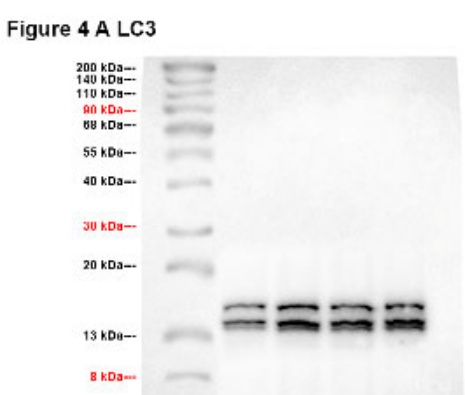

LC3（14,16kDa）.tif

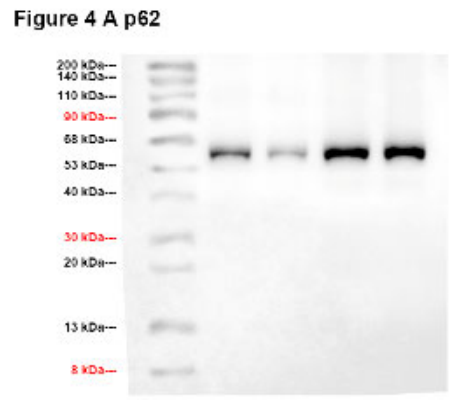

p62（62kDa）.tif

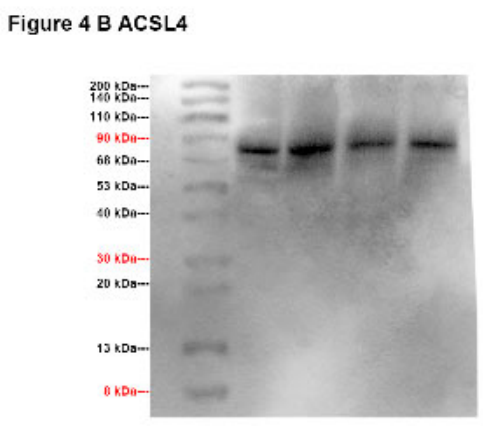

ACSL4（79kDa）.tif

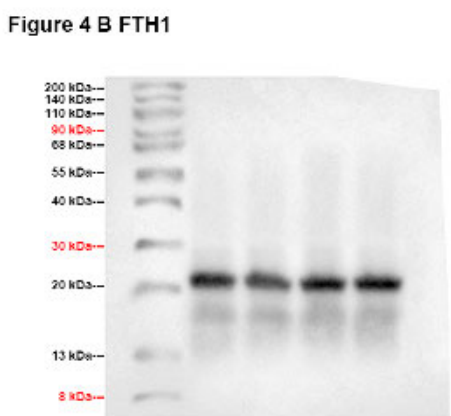

FTH1（21kDa）.tif

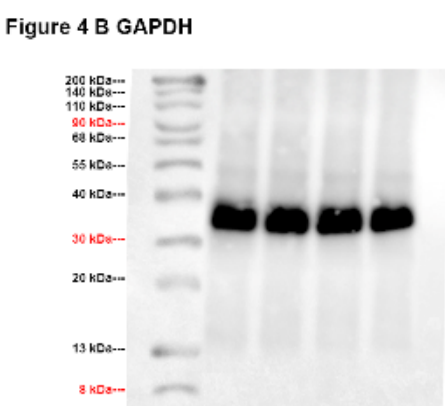

GAPDH（36kDa）.tif

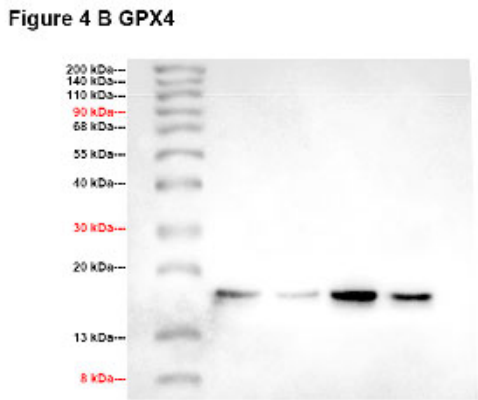

GPX4（17kDa）.tif

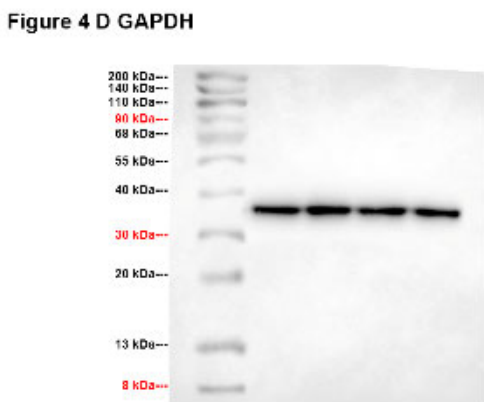

GAPDH（36kDa）.tif

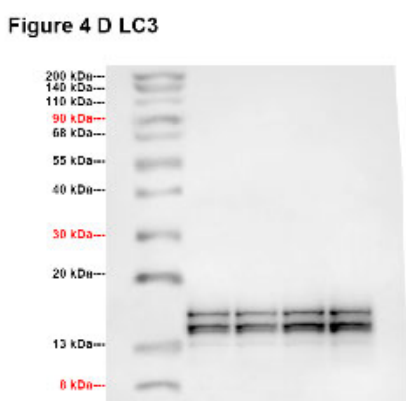

LC3（14,16kDa）.tif

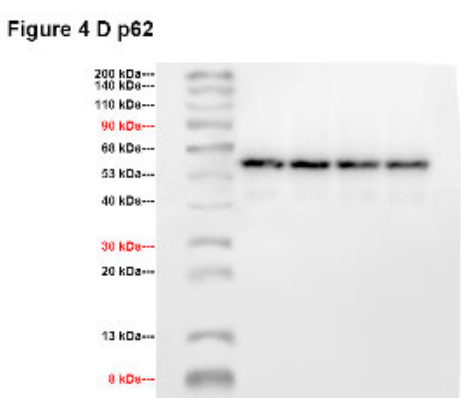

p62（62kDa）.tif

Figure 4 E ATG5

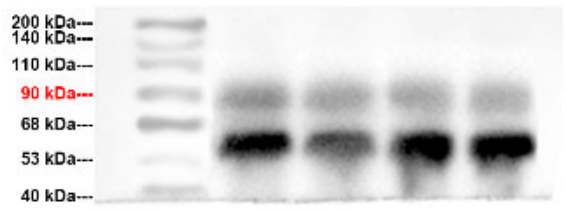

ATG5（55kDa）.tif

Figure 4 E ATG7

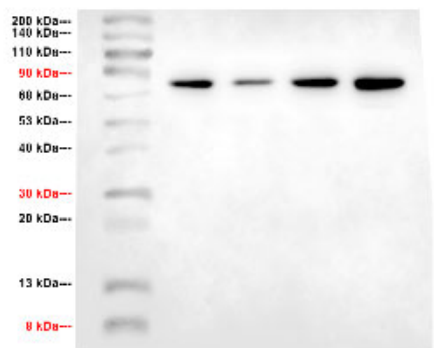

ATG7（78kDa）.tif

Figure 4 E Beclin 1

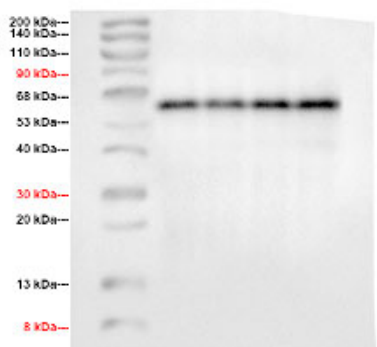

Beclin 1（60kDa）.tif

Figure 4 E GAPDH

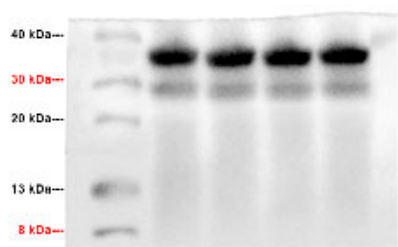

GAPDH（36kDa）.tif

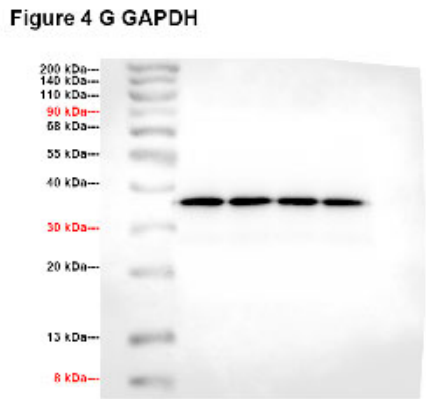

GAPDH（36kDa）.tif

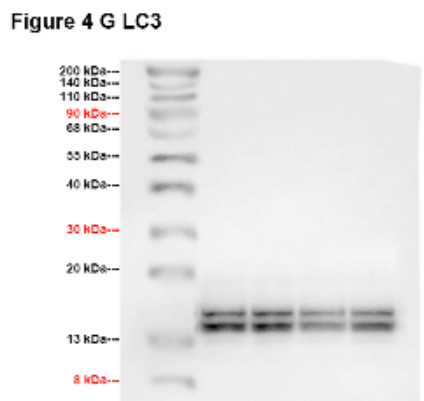

LC3（14,16kDa）.tif

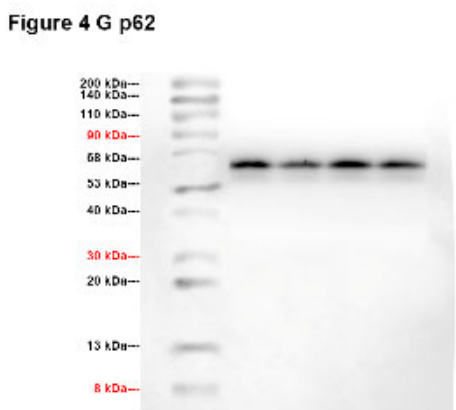

p62（62kDa）.tif

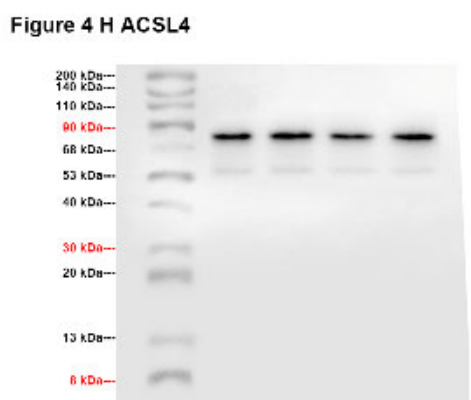

ACSL4（79kDa）.tif

Figure 4 H FTH1

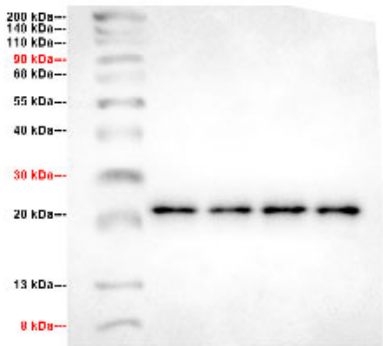

FTH1（21kDa）.tif

Figure 4 H GAPDH

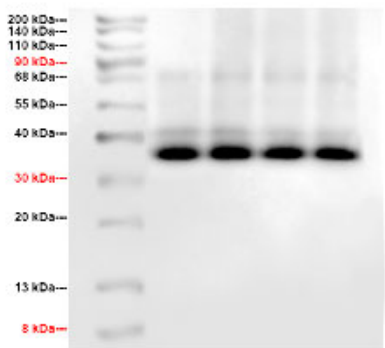

GAPDH（36kDa）.tif

Figure 4 H GPX4

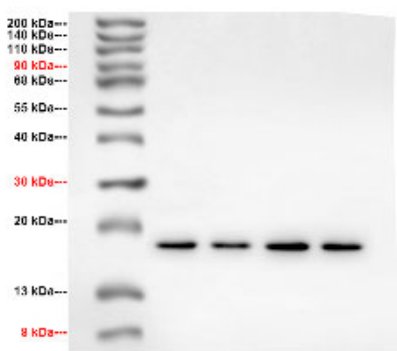

GPX4（17kDa）.tif

Figure 5 C shCYLD GAPDH

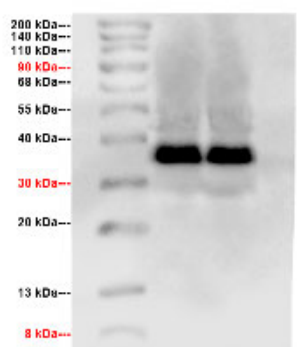

GAPDH（36kDa）.tif

Figure 5 C shCYLD MAPK12

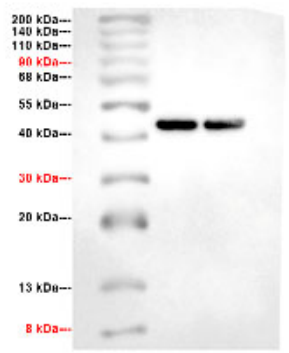

MAPK12（42kDa）.tif

Figure 5 C shUSP20 GAPDH

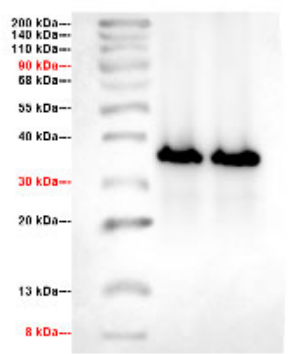

GAPDH（36kDa）.tif

Figure 5 C shUSP20 MAPK12

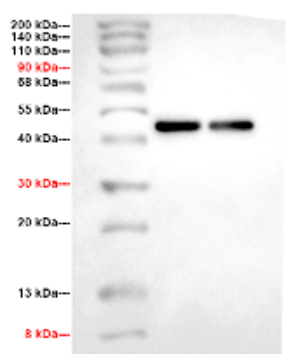

MAPK12（42kDa）.tif

Figure 5 C shUSP32 GAPDH

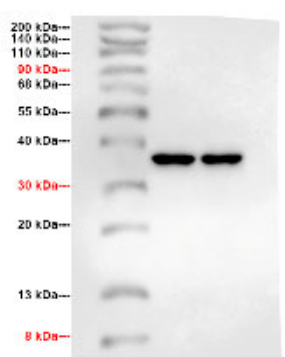

GAPDH（36kDa）.tif

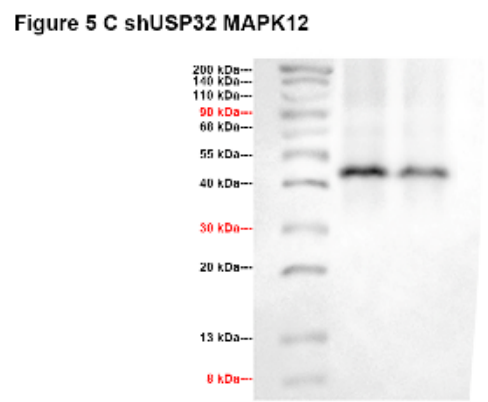

MAPK12（42kDa）.tif

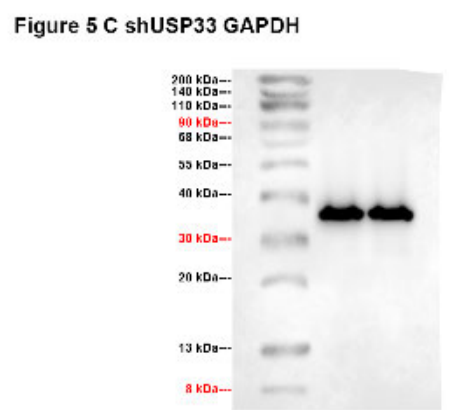

GAPDH（36kDa）.tif

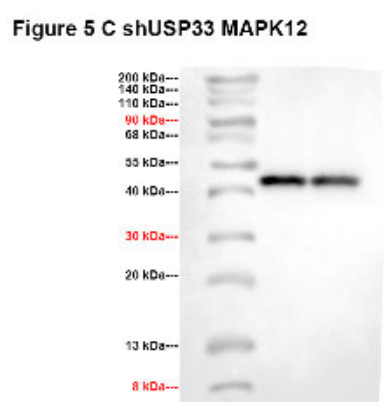

MAPK12（42kDa）.tif

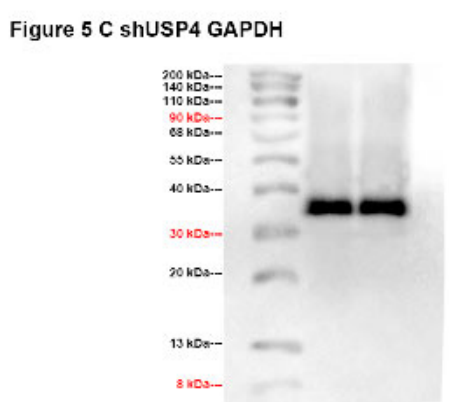

GAPDH（36kDa）.tif

Figure 5 C shUSP4 MAPK12

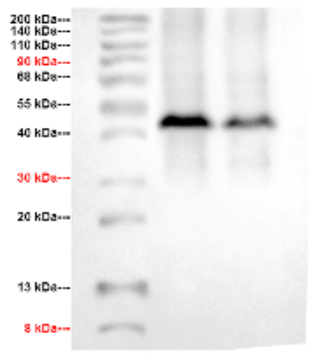

MAPK12（42kDa）.tif

Figure 5 C shUSP7 GAPDH

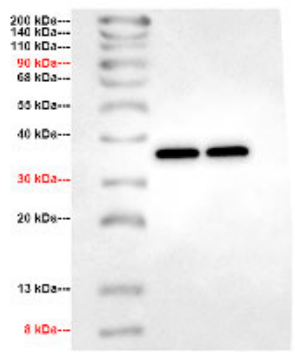

GAPDH（36kDa）.tif

Figure 5 C shUSP7 MAPK12

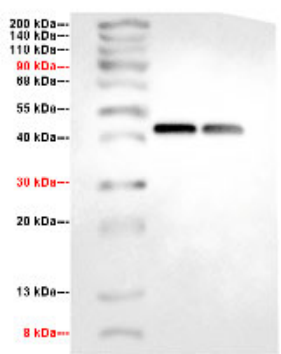

MAPK12（42kDa）.tif

Figure 5 F H1299 GAPDH

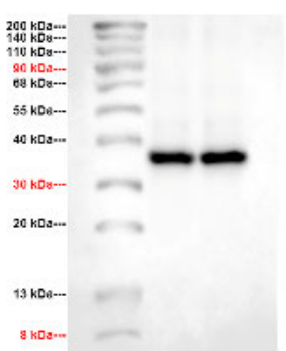

GAPDH（36kDa）.tif

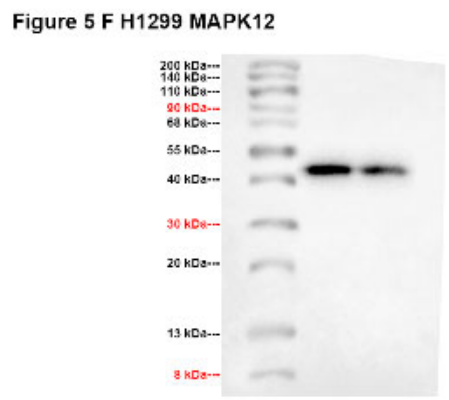

MAPK12（42kDa）.tif

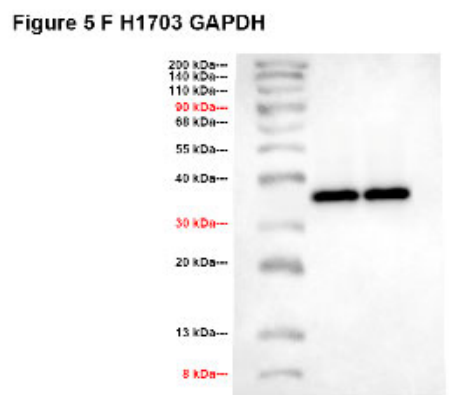

GAPDH（36kDa）.tif

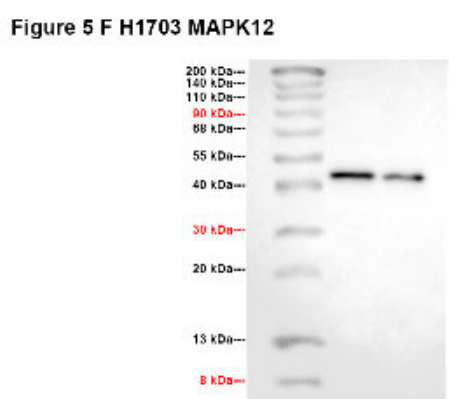

MAPK12（42kDa）.tif

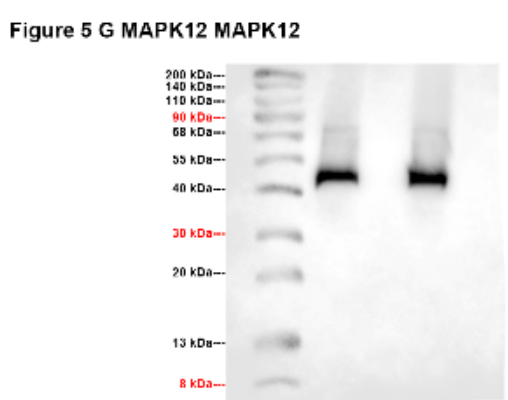

MAPK12（42kDa）.tif

Figure 5 G MAPK12 USP32

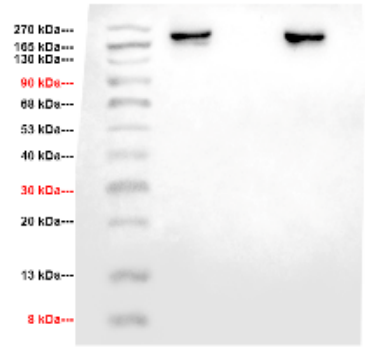

USP32（240kDa）.tif

Figure 5 G USP32 MAPK12

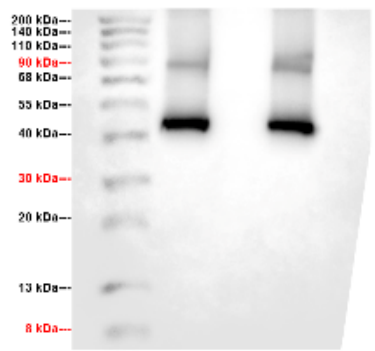

MAPK12（42kDa）.tif

Figure 5 G USP32 USP32

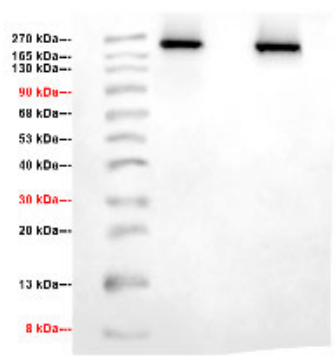

USP32（240kDa）.tif

Figure 5 H Anti-Flag

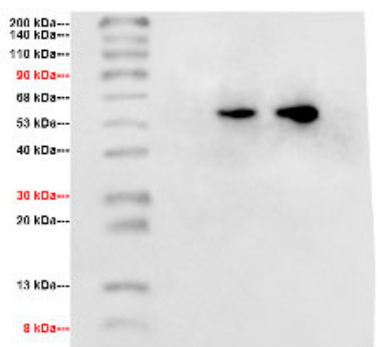

Anti-Flag（54kDa）.tif

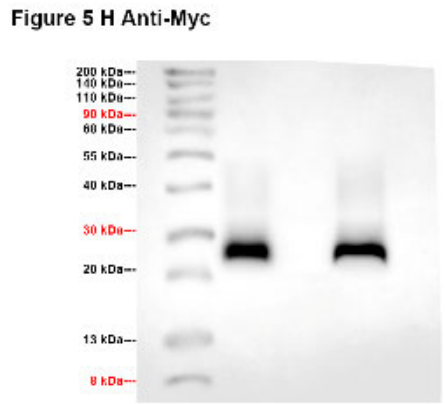

Anti-Myc（25kDa）.tif

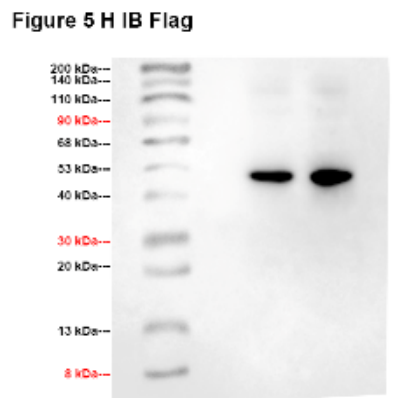

IB Flag（51kDa）.tif

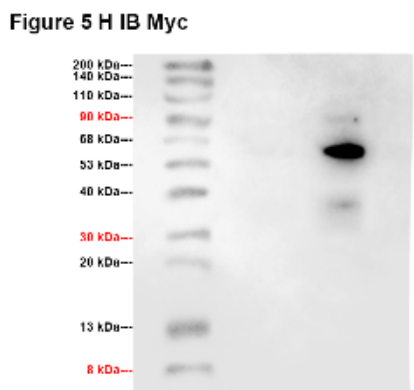

IB Myc（58kDa）.tif

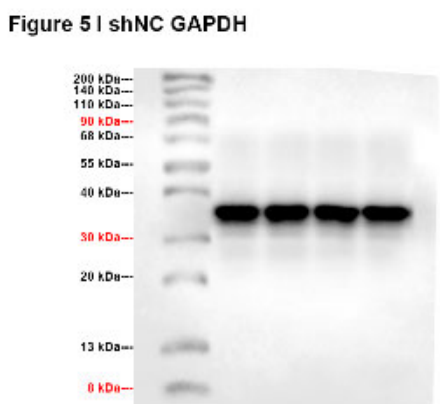

GAPDH（36kDa）.tif

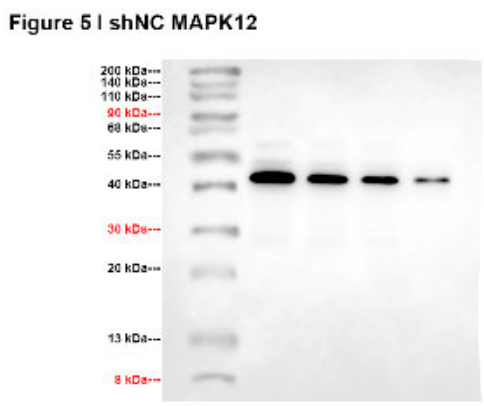

MAPK12（42kDa）.tif

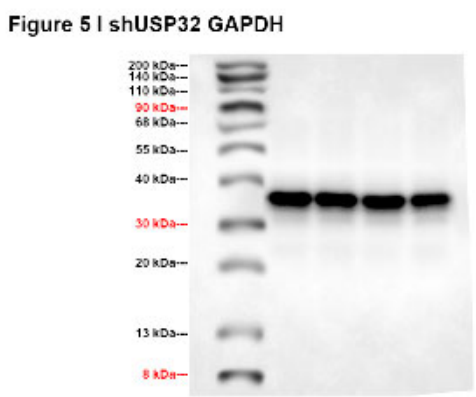

GAPDH（36kDa）.tif

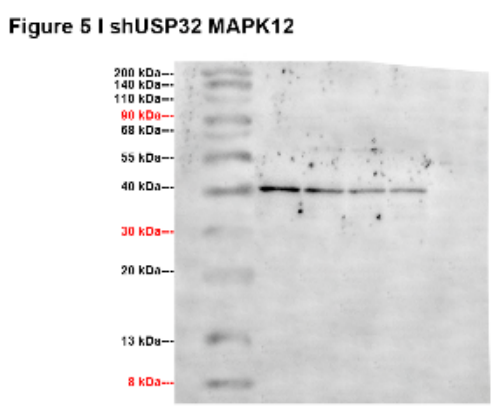

MAPK12（42kDa）.tif

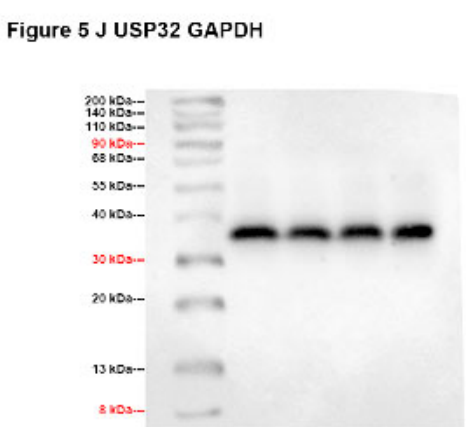

GAPDH（36kDa）.tif

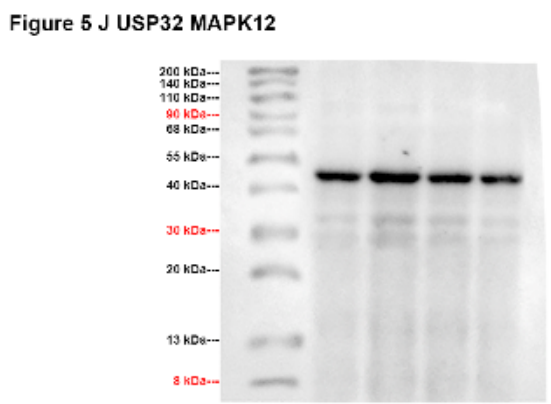

MAPK12（42kDa）.tif

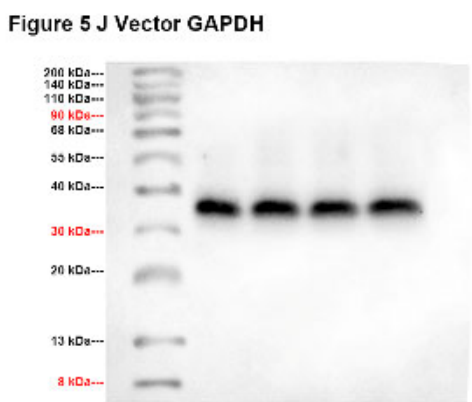

GAPDH（36kDa）.tif

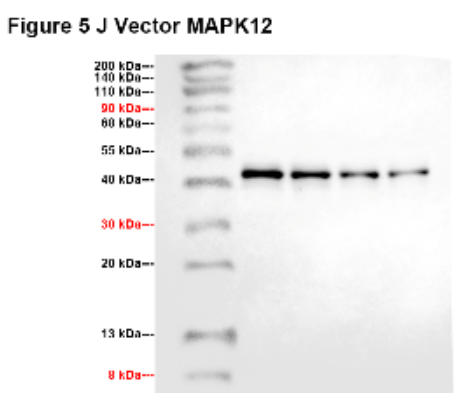

MAPK12（42kDa）.tif

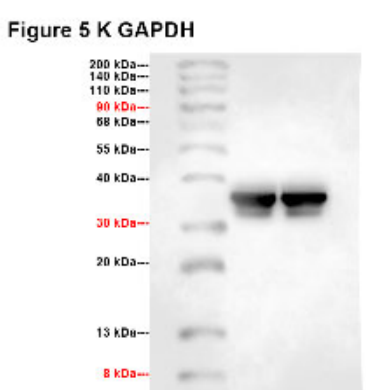

GAPDH（36kDa）.tif

Figure 5 K IB Ub

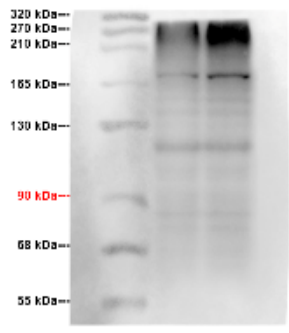

IB Ub（170kDa）.tif

Figure 5 K MAPK12

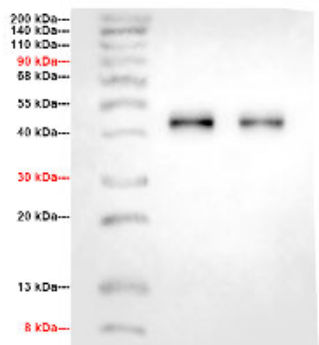

MAPK12（42kDa）.tif

Figure 5 K USP32

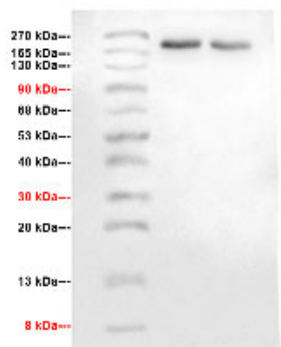

USP32（240kDa）.tif

Figure 5 L GAPDH

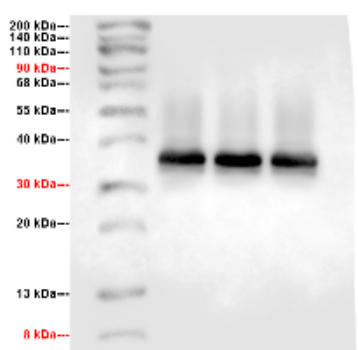

GAPDH（36kDa）.tif

Figure 5 L MAPK12

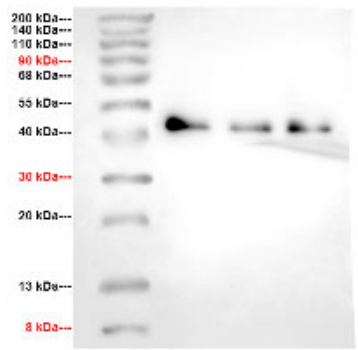

MAPK12（42kDa）.tif

Figure 5 L USP32

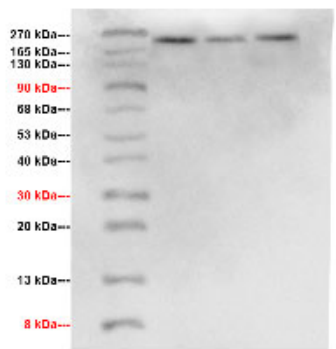

USP32（240kDa）.tif

Figure 5 M GAPDH

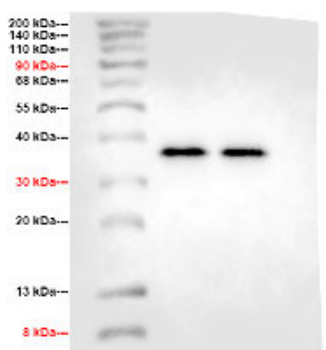

GAPDH（36kDa）.tif

Figure 5 M IB Ub

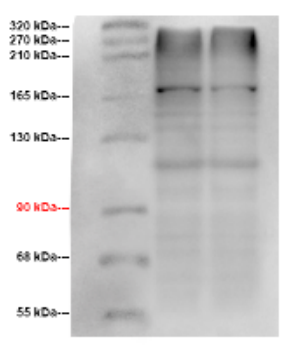

IB Ub（170kDa）.tif

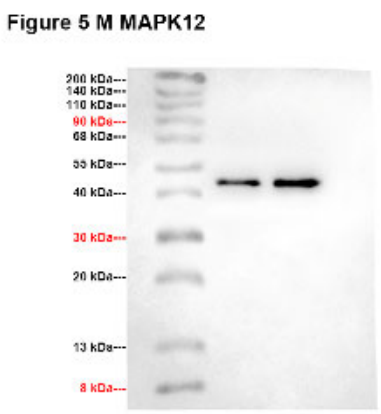

MAPK12（42kDa）.tif

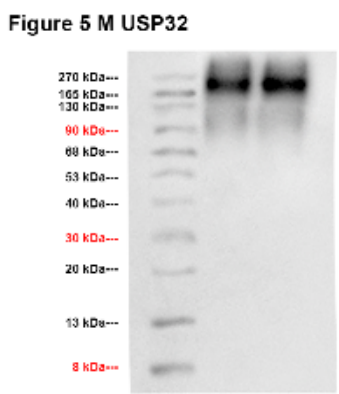

USP32（240kDa）.tif

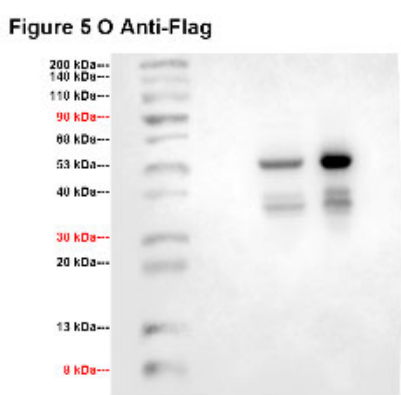

Anti-Flag（54kDa）.tif

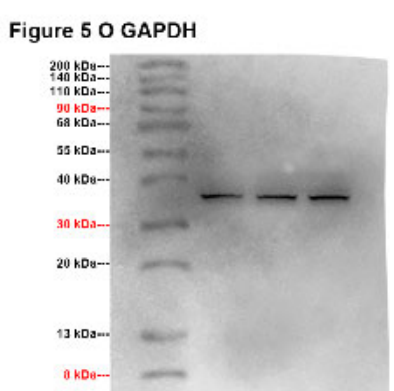

GAPDH（36kDa）.tif

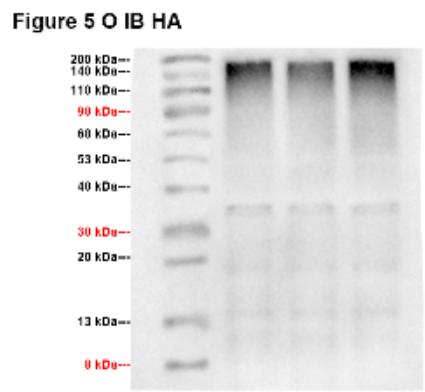

IB HA（175kDa）.tif

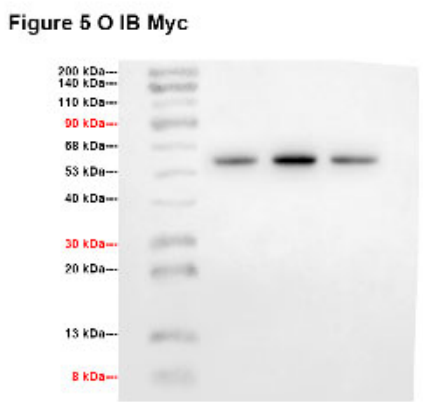

IB Myc（58kDa）.tif

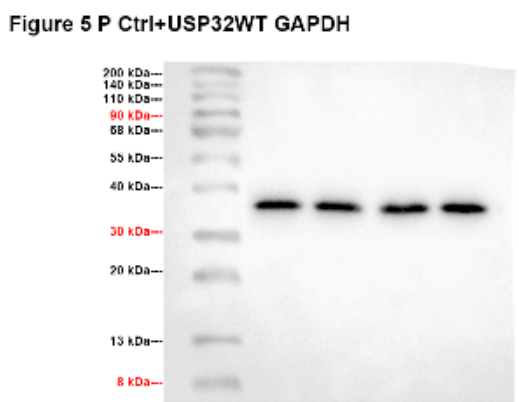

GAPDH（36kDa）.tif

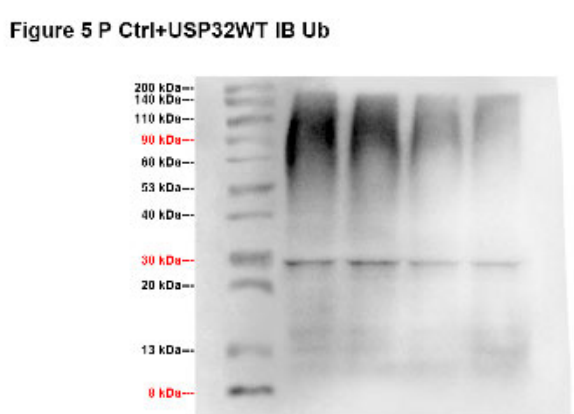

IB Ub（170kDa）.tif

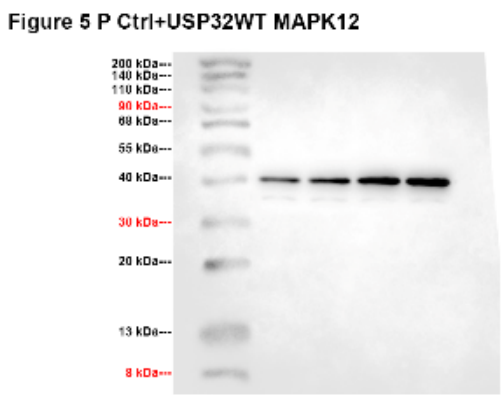

MAPK12（42kDa）.tif

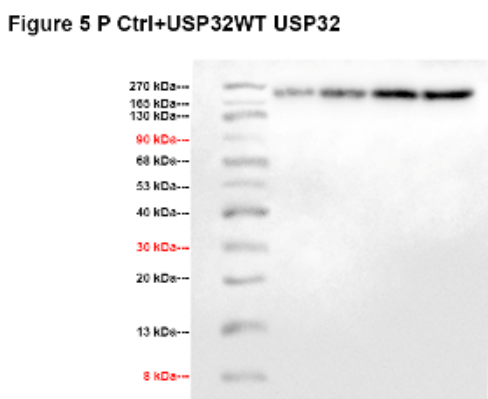

USP32（240kDa）.tif

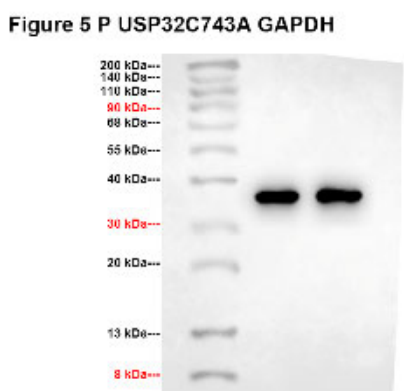

GAPDH（36kDa）.tif

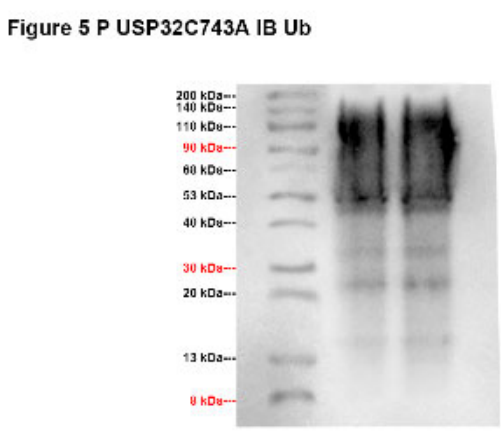

IB Ub（170kDa）.tif

Figure 5 P USP32C743A MAPK12

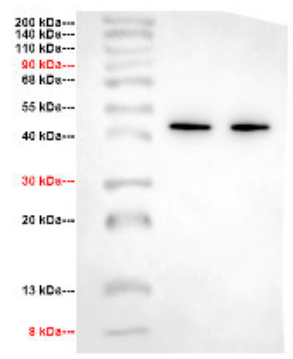

MAPK12（42kDa）.tif

Figure 5 P USP32C743A USP32

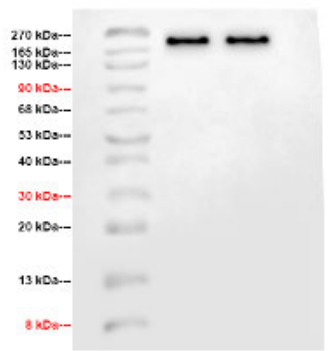

USP32（240kDa）.tif

Figure 5 Q USP32C742A GAPDH

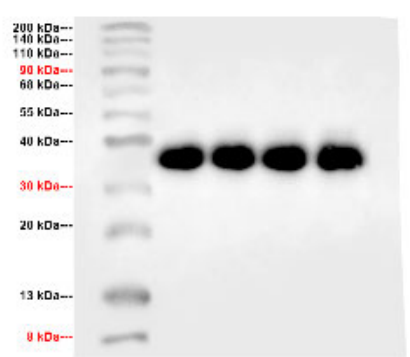

GAPDH（36kDa）.tif

Figure 5 Q USP32C742A MAPK12

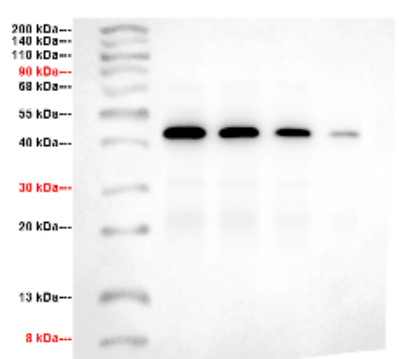

MAPK12（42kDa）.tif

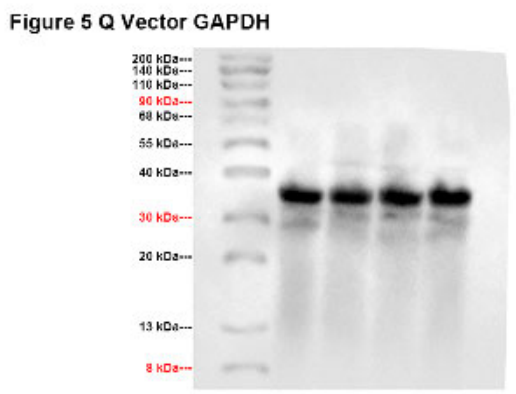

GAPDH（36kDa）.tif

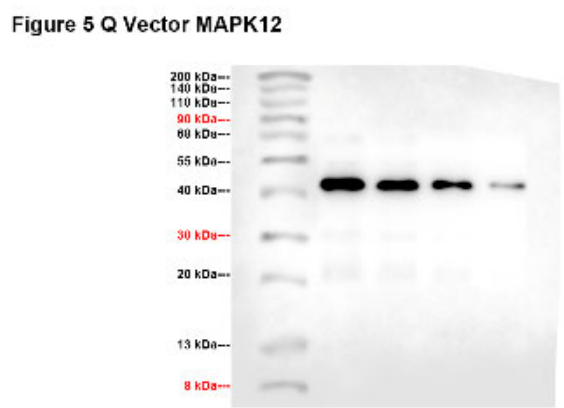

MAPK12（42kDa）.tif

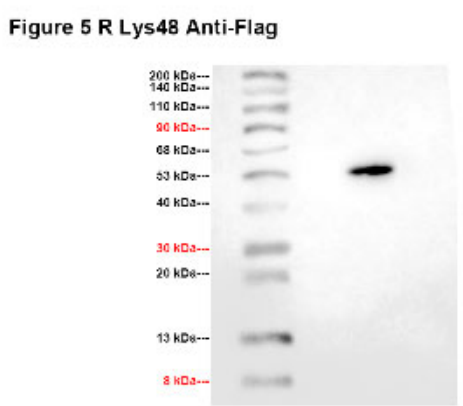

Anti-Flag（54kDa）.tif

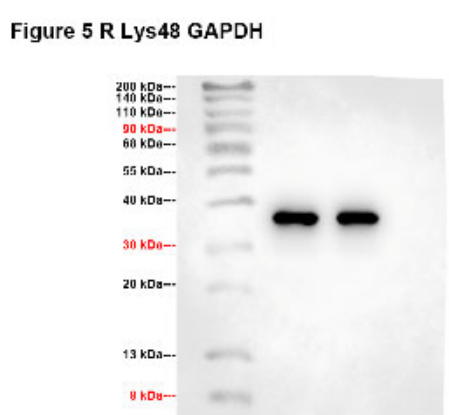

GAPDH（36kDa）.tif

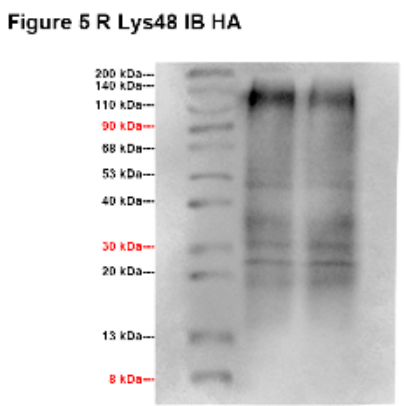

IB HA（175kDa）.tif

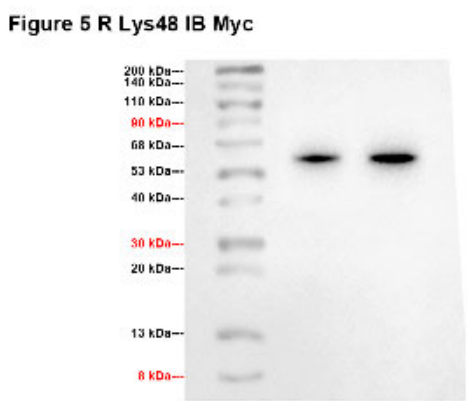

IB Myc（58kDa）.tif

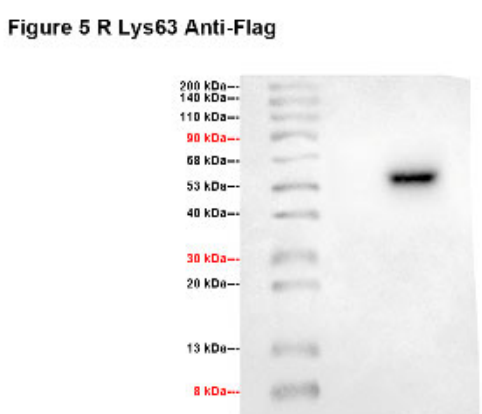

Anti-Flag（54kDa）.tif

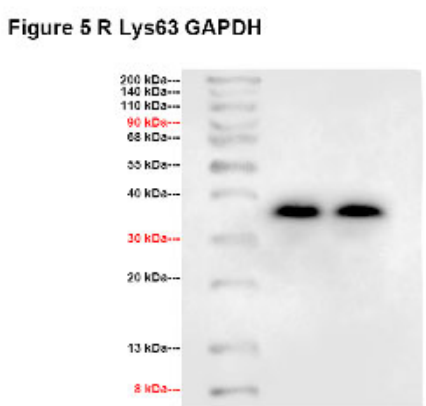

GAPDH（36kDa）.tif

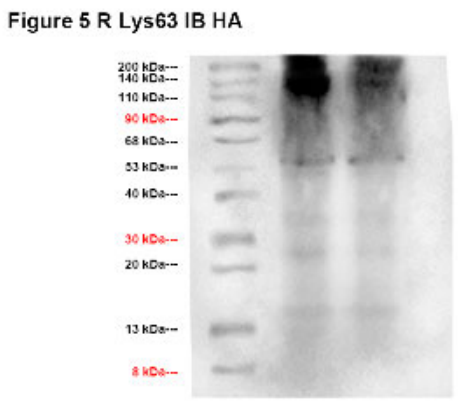

IB HA（175kDa）.tif

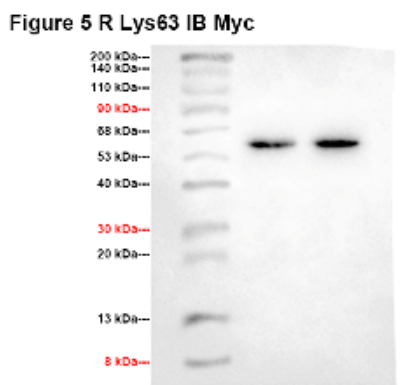

IB Myc（58kDa）.tif

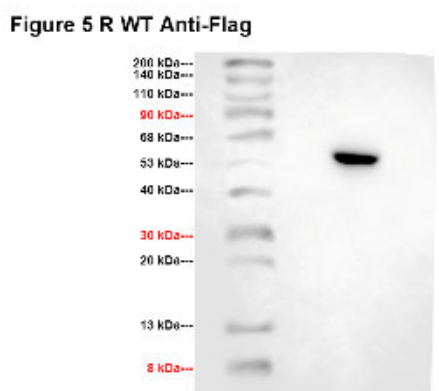

Anti-Flag（54kDa）.tif

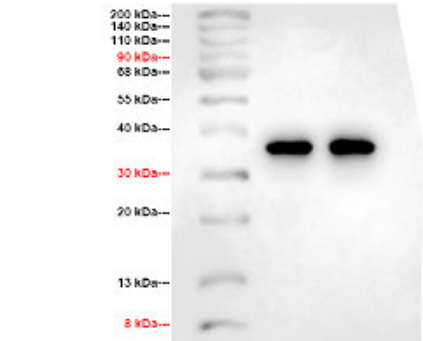

GAPDH（36kDa）.tif

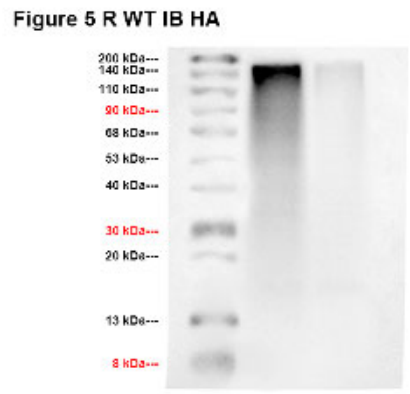

IB HA（175kDa）.tif

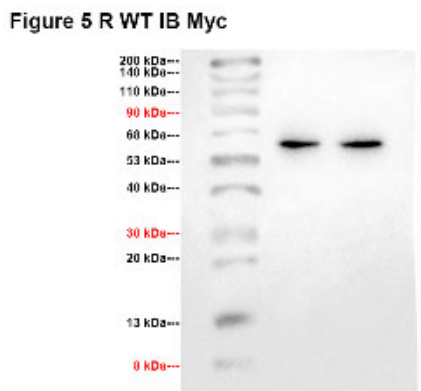

IB Myc（58kDa）.tif

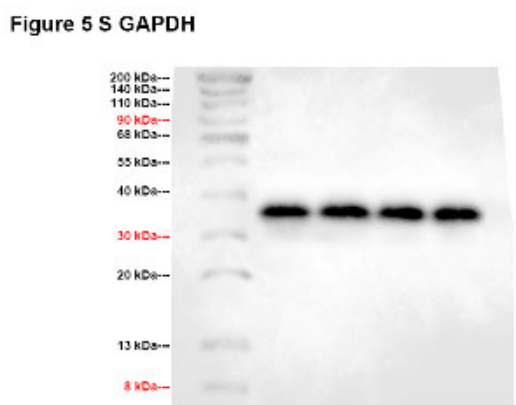

GAPDH（36kDa）.tif

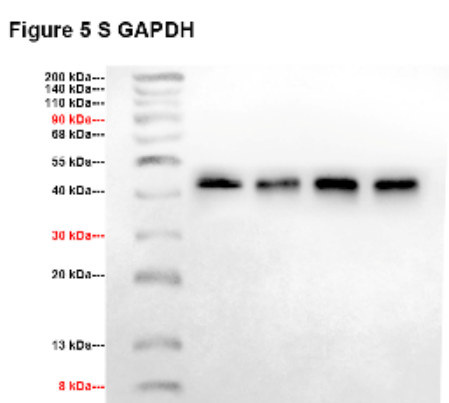

MAPK12（42kDa）.tif

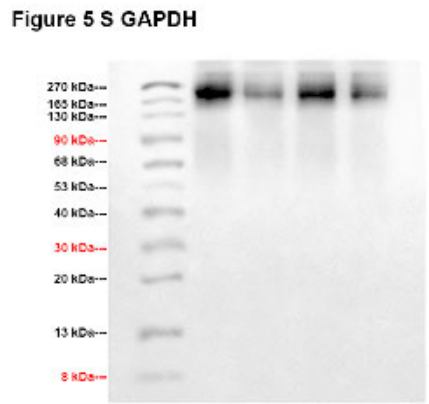

USP32（240kDa）.tif

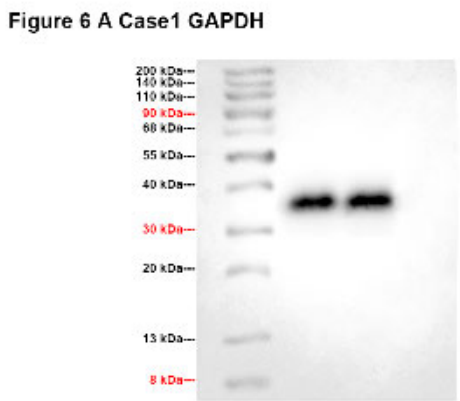

GAPDH（36kDa）.tif

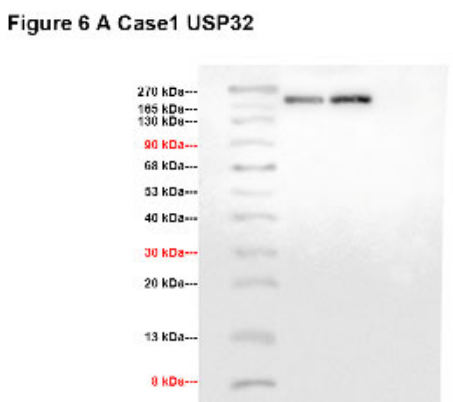

USP32（240kDa）.tif

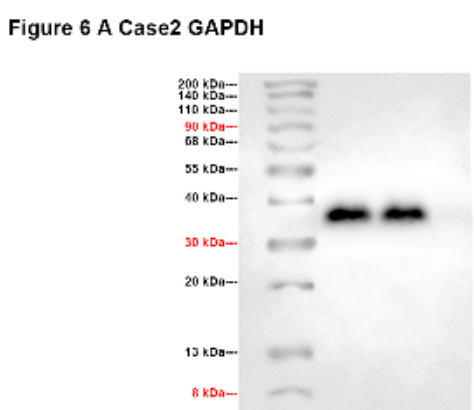

GAPDH（36kDa）.tif

Figure 6 A Case2 USP32

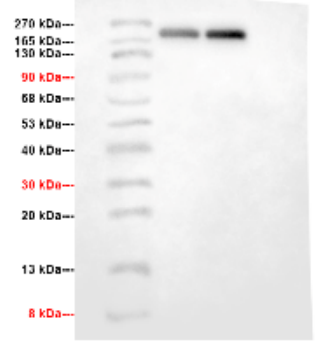

USP32（240kDa）.tif

Figure 6 A Case3 GAPDH

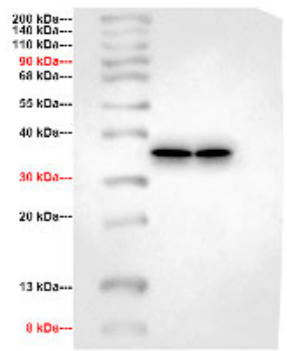

GAPDH（36kDa）.tif

Figure 6 A Case3 USP32

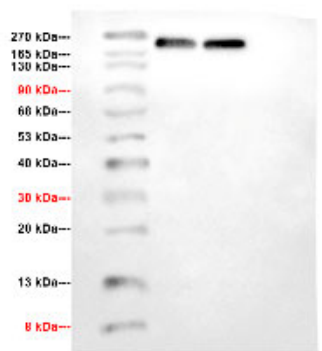

USP32（240kDa）.tif

Figure 6 A Case4 GAPDH

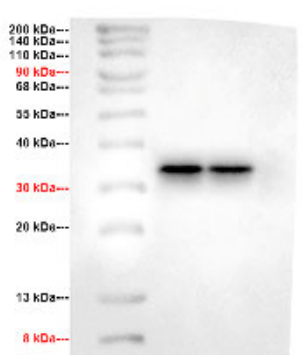

GAPDH（36kDa）.tif

Figure 6 A Case4 USP32

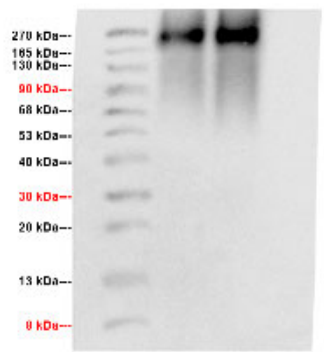

USP32（240kDa）.tif

Figure 6 B A549 GAPDH

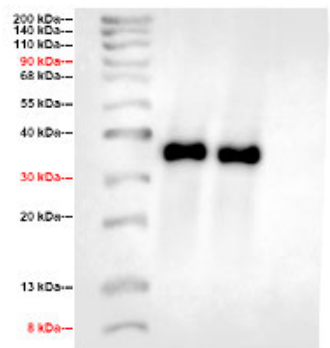

GAPDH（36kDa）.tif

Figure 6 B A549 USP32

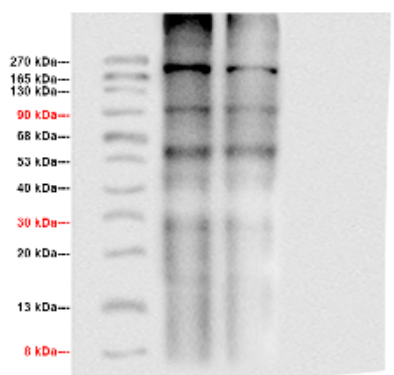

USP32（240kDa）.tif

Figure 6 B H1703 GAPDH

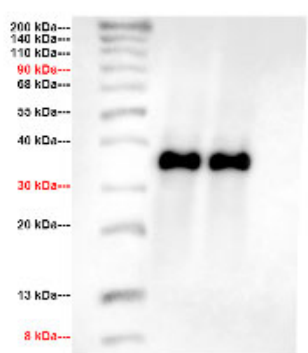

GAPDH（36kDa）.tif

Figure 6 B H1703 USP32

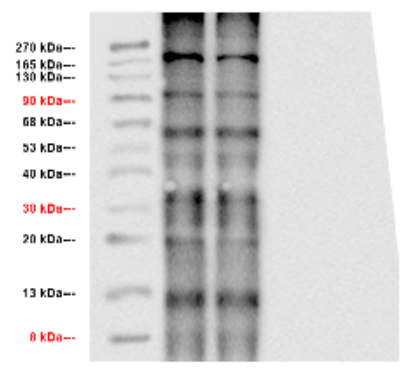

USP32（240kDa）.tif

Figure 6 H ACSL4

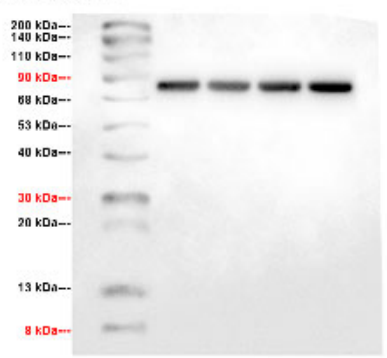

ACSL4（79kDa）.tif

Figure 6 H FTH1

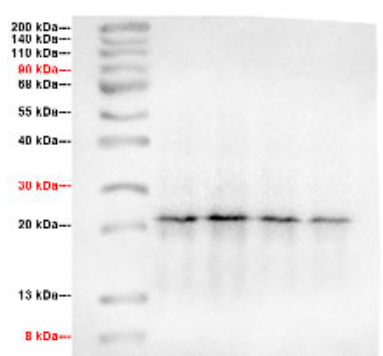

FTH1（21kDa）.tif

Figure 6 H GAPDH

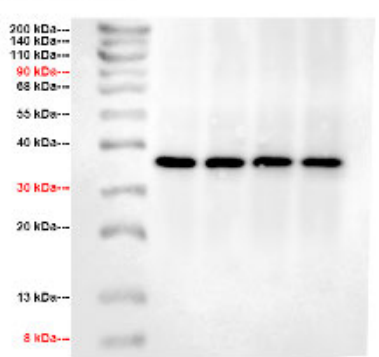

GAPDH（36kDa）.tif

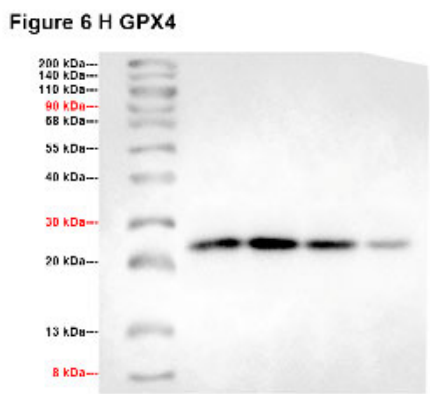

GPX4（22kDa）.tif

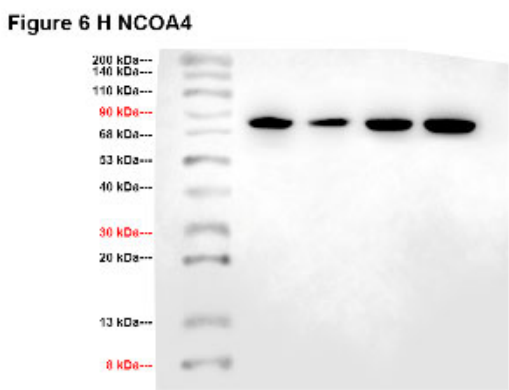

NCOA4（70kDa）.tif

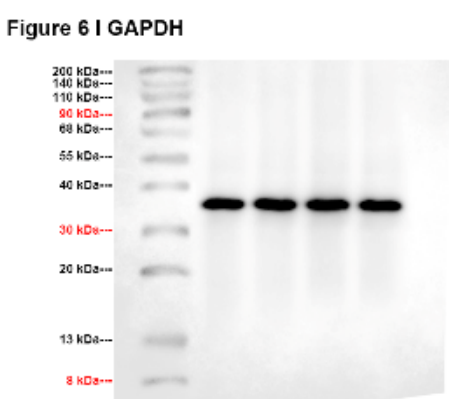

GAPDH（36kDa）.tif

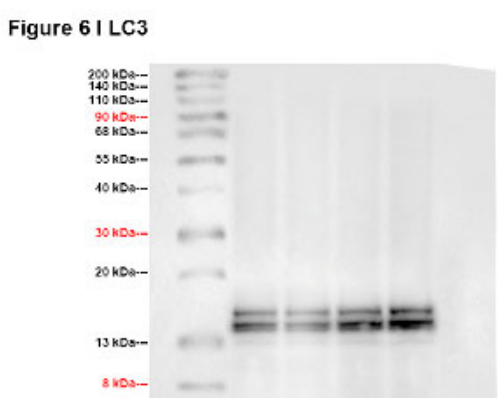

LC3（14,16kDa）.tif

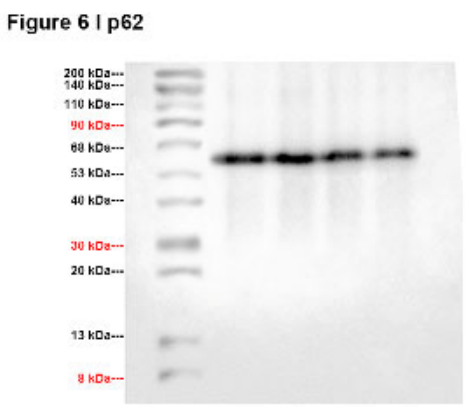

p62（62kDa）.tif

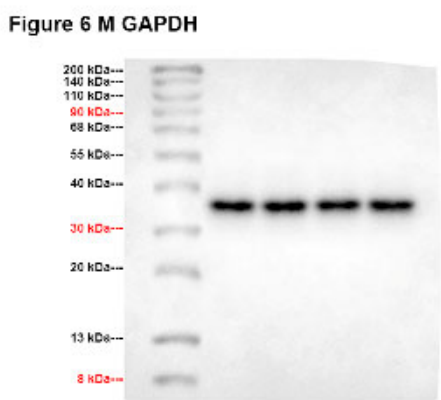

GAPDH（36kDa）.tif

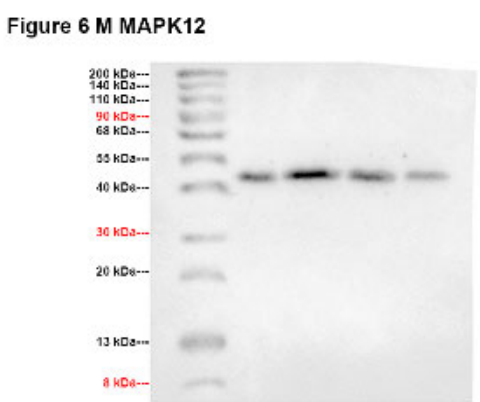

MAPK12（42kDa）.tif

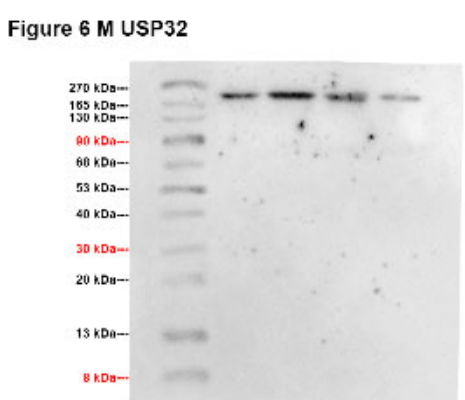

USP32（240kDa）.tif

Figure 6 N ACSL4

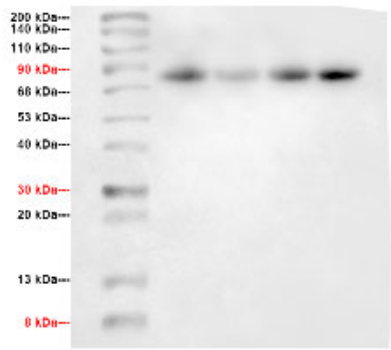

ACSL4（79kDa）.tif

Figure 6 N FTH1

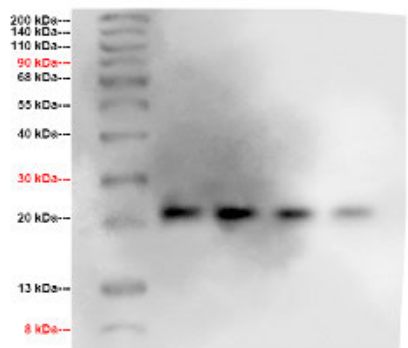

FTH1（21kDa）.tif

Figure 6 N GAPDH

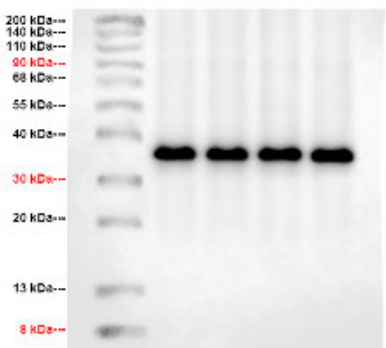

GAPDH（36kDa）.tif

Figure 6 N GPX4

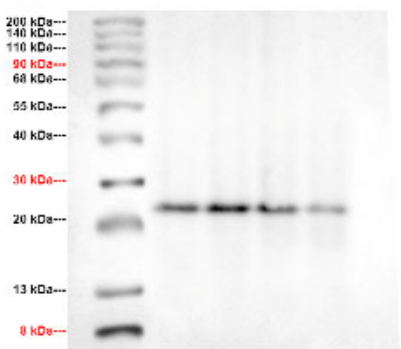

GPX4（22kDa）.tif

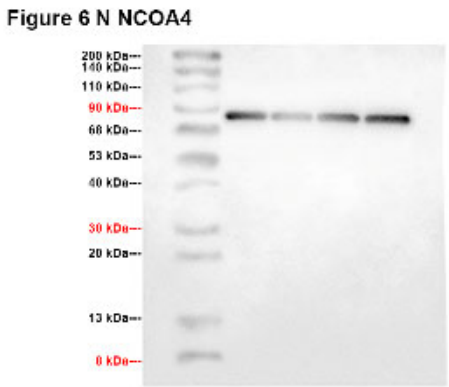

NCOA4（70kDa）.tif

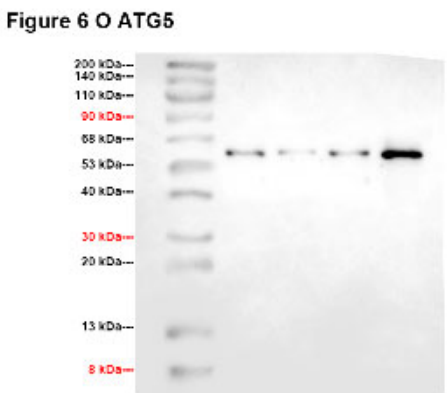

ATG5（55kDa）.tif

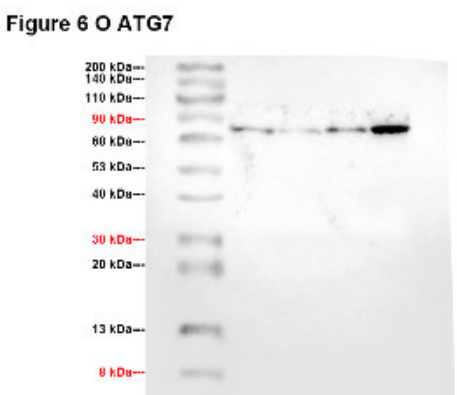

ATG7（78kDa）.tif

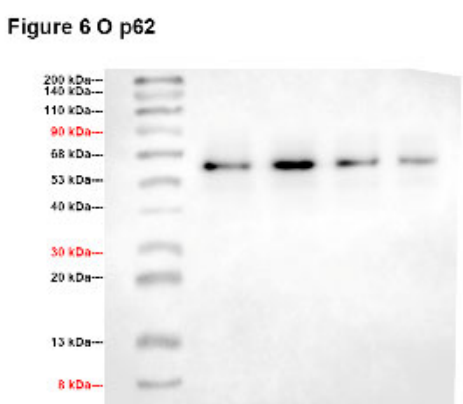

p62（62kDa）.tif

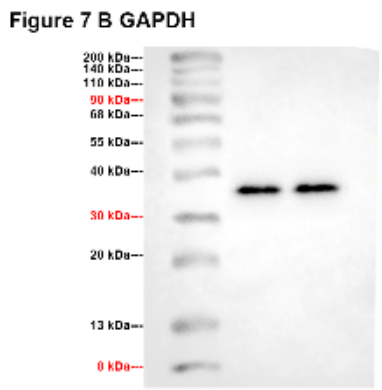

GAPDH（36kDa）.tif

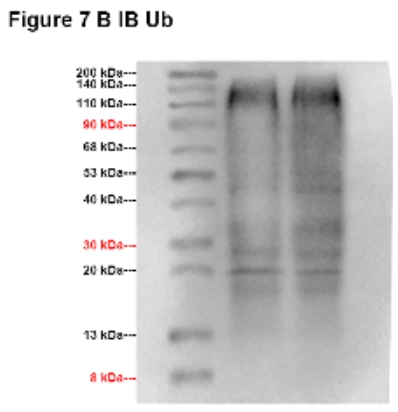

IB Ub（170kDa）.tif

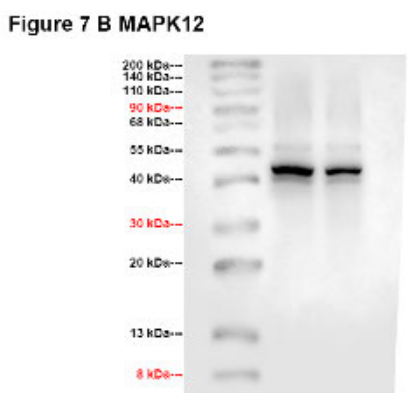

MAPK12（42kDa）.tif

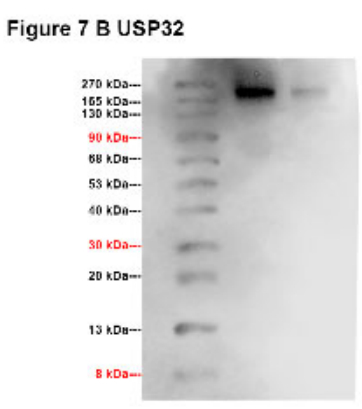

USP32（240kDa）.tif

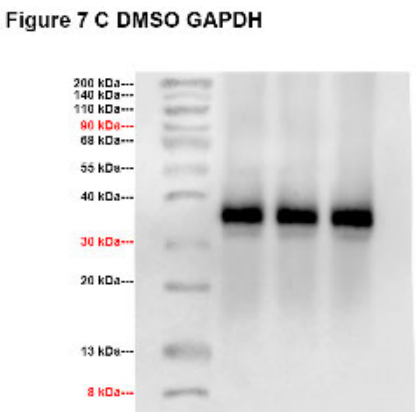

GAPDH（36kDa）.tif

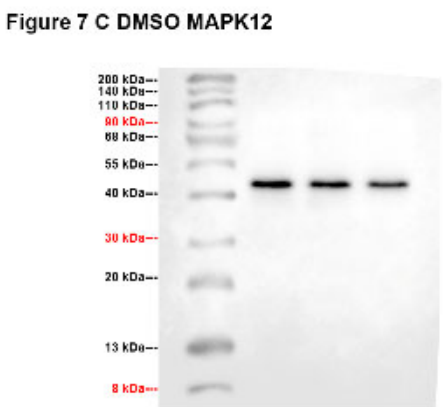

MAPK12（42kDa）.tif

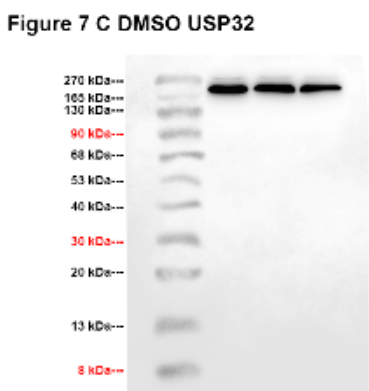

USP32（240kDa）.tif

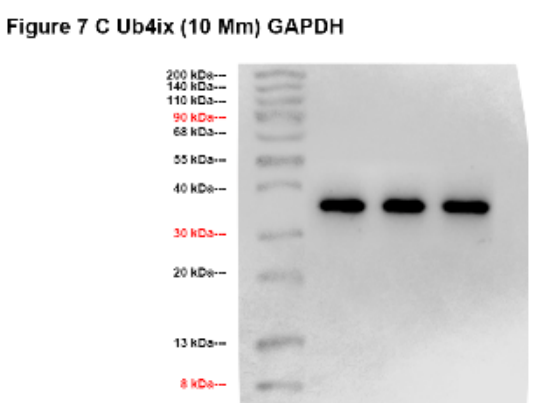

GAPDH（36kDa）.tif

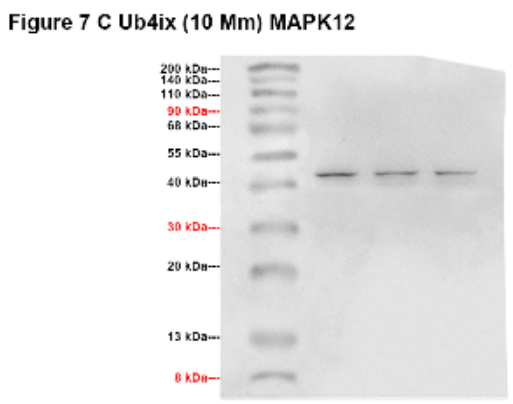

MAPK12（42kDa）.tif

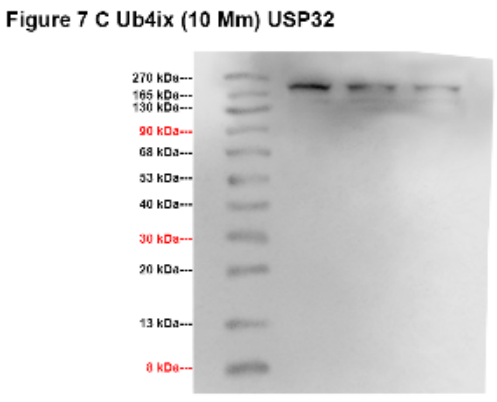

USP32（240kDa）.tif

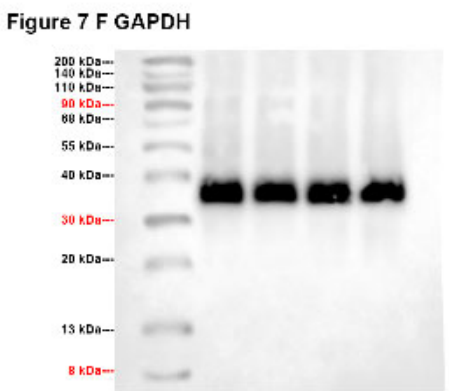

GAPDH（36kDa）.tif

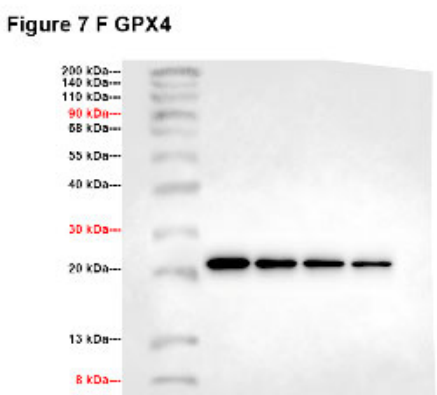

GPX4（22kDa）.tif

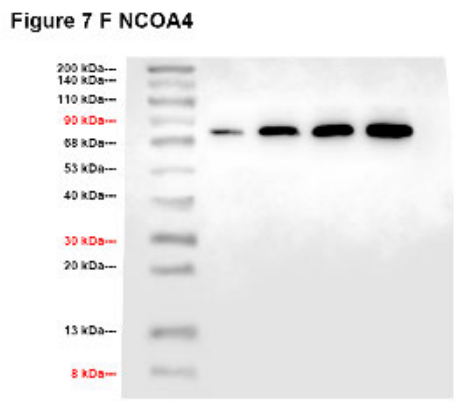

NCOA4（70kDa）.tif

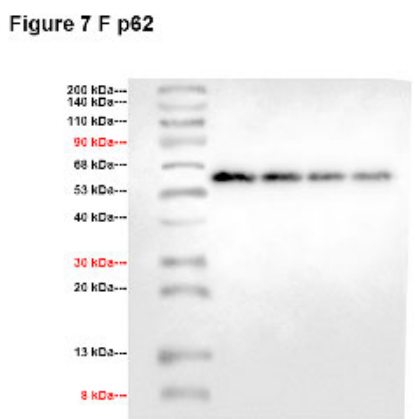

p62（62kDa）.tif

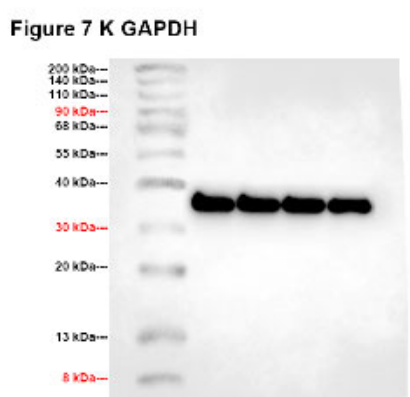

GAPDH（36kDa）.tif

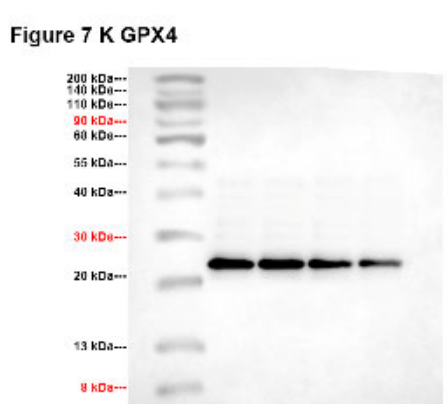

GPX4（22kDa）.tif

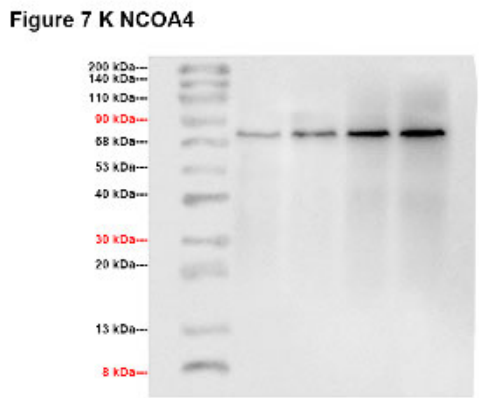

NCOA4（70kDa）.tif

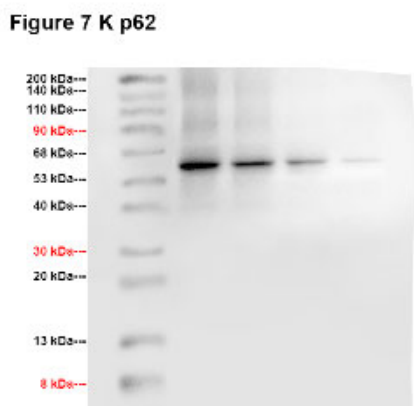

p62（62kDa）.tif

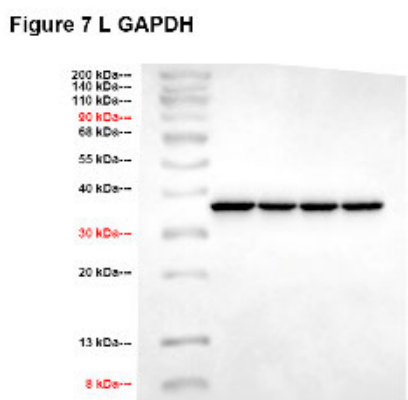

GAPDH（36kDa）.tif

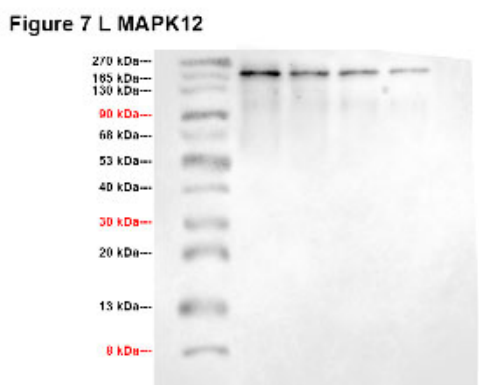

MAPK12（240kDa）.tif

Figure 7 L USP32

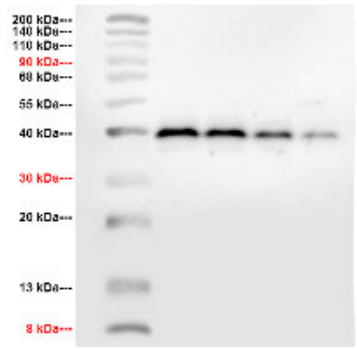

USP32（42kDa）.tif

Figure S1 A A549 GAPDH

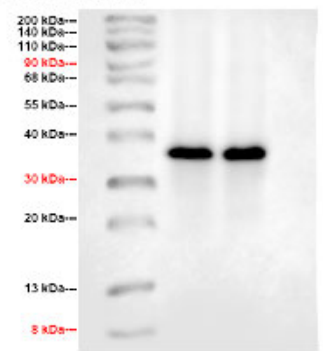

GAPDH（36kDa）.tif

Figure S1 A A549 MAPK12

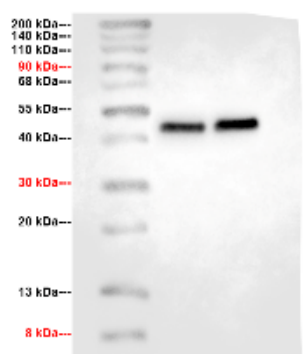

MAPK12（42kDa）.tif

Figure S1 A PC-9 GAPDH

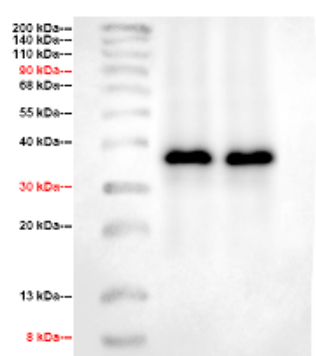

GAPDH（36kDa）.tif

Figure S1 A PC-9 MAPK12

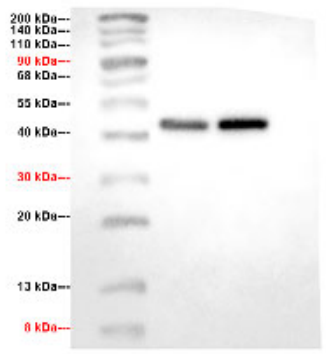

MAPK12（42kDa）.tif

Figure S1 C H1299 GAPDH

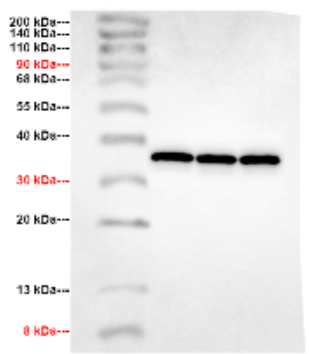

GAPDH（36kDa）.tif

Figure S1 C H1299 MAPK12

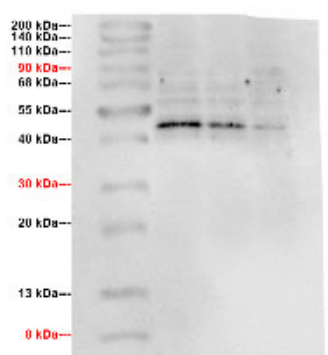

MAPK12（42kDa）.tif

Figure S1 C H1703 GAPDH

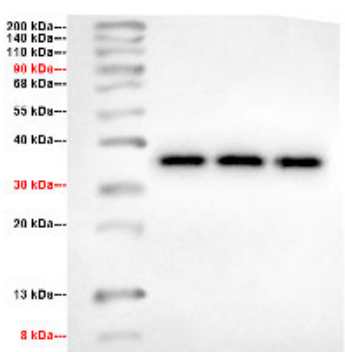

GAPDH（36kDa）.tif

Figure S1 C H1703 MAPK12

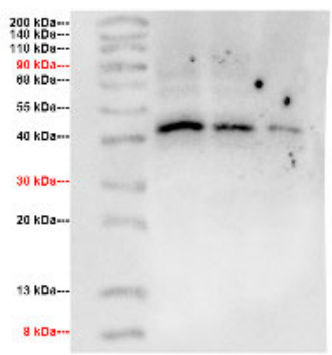

MAPK12（42kDa）.tif

Figure S1 D H1299 GAPDH

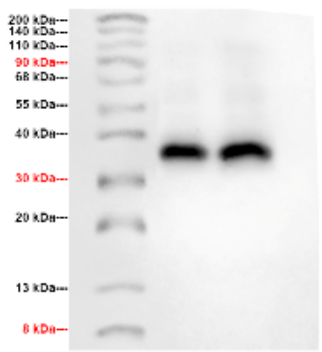

GAPDH（36kDa）.tif

Figure S1 D H1299 MAPK11

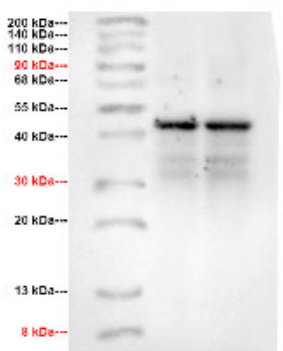

MAPK11（42kDa）.tif

Figure S1 D H1299 MAPK14

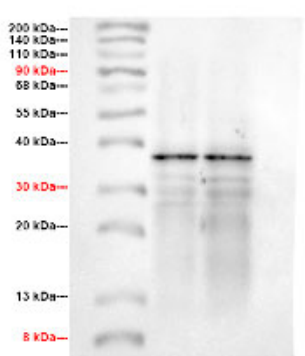

MAPK14（38kDa）.tif

Figure S1 D H1703 GAPDH

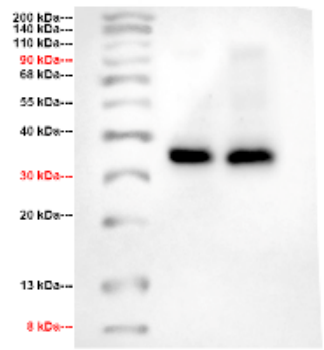

GAPDH（36kDa）.tif

Figure S1 D H1703 MAPK11

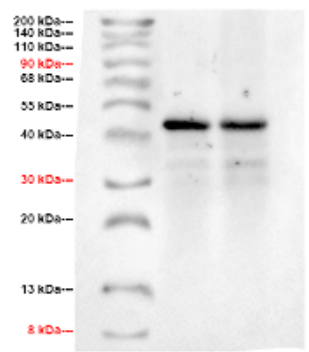

MAPK11（42kDa）.tif

Figure S1 D H1703 MAPK14

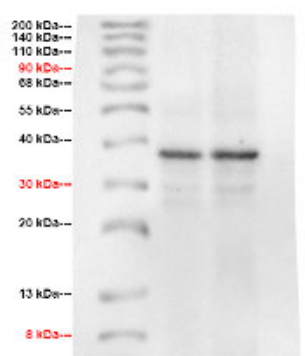

MAPK14（38kDa）.tif

Figure S1 E A549 GAPDH

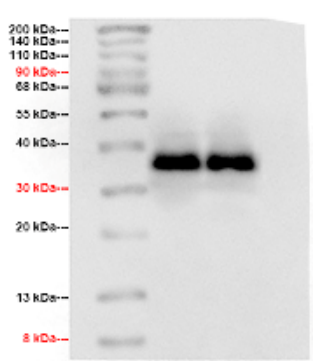

GAPDH（36kDa）.tif

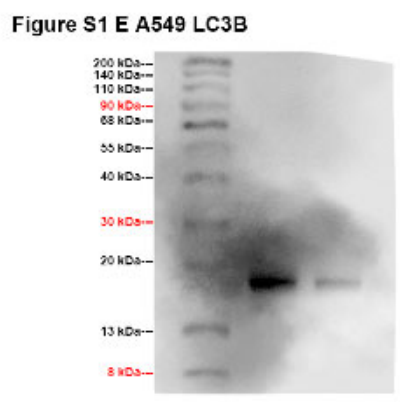

LC3B（17kDa）.tif

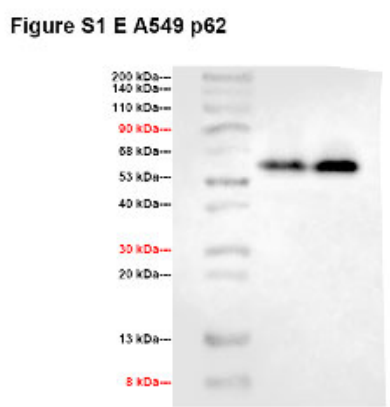

p62（62kDa）.tif

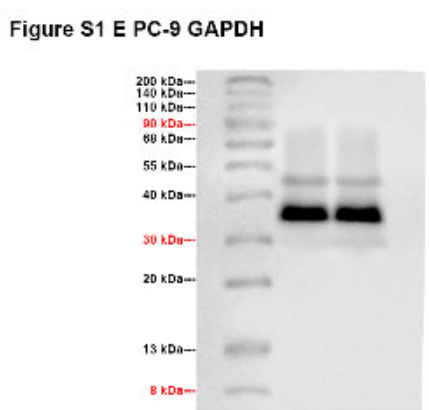

GAPDH（36kDa）.tif

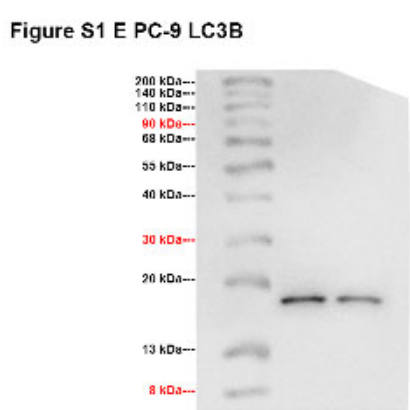

LC3B（17kDa）.tif

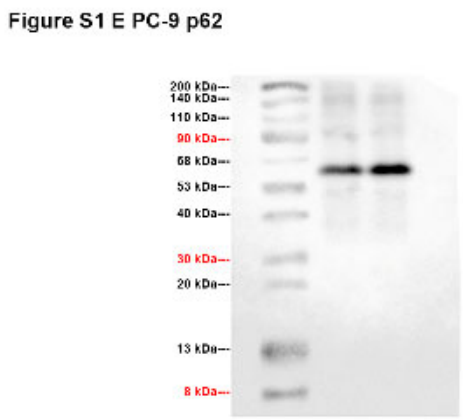

p62（62kDa）.tif

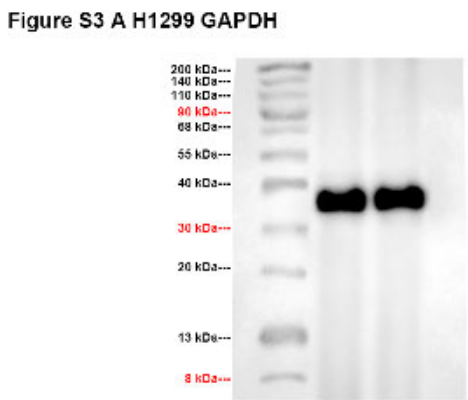

GAPDH(36kDa).tif

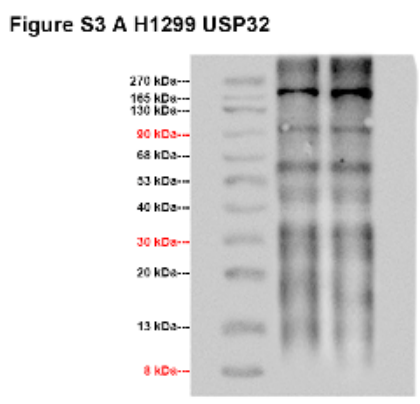

USP32（240kDa）.tif

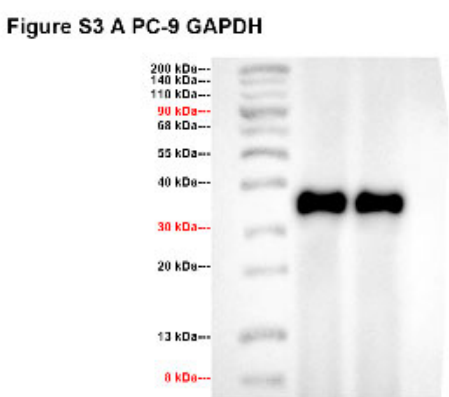

GAPDH (36kDa).tif

Figure S3 A PC-9 USP32

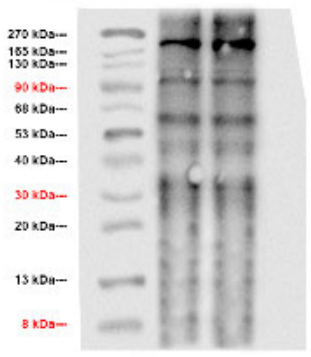

USP32（240kDa）.tif
