# Supplementary material for: USP32-mediated stabilization of MAPK12 promotes lung adenocarcinoma progression by inhibiting autophagy-ferroptosis
Source: Commun Biol. 2026 Jul 19;9:1505. doi: 10.1038/s42003-026-10429-z (PMC13751611; doi:10.1038/s42003-026-10429-z)
Supplement: Supplementary file 2 [file 42003_2026_10429_MOESM2_ESM.pdf]

Description of Additional Supplementary Files

File name: Supplementary Data

Description: Source data for the figures
