# Supplementary material for: USP32-mediated stabilization of MAPK12 promotes lung adenocarcinoma progression by inhibiting autophagy-ferroptosis
Source: Commun Biol. 2026 Jul 19;9:1505. doi: 10.1038/s42003-026-10429-z (PMC13751611; doi:10.1038/s42003-026-10429-z)
Supplement: Supplementary file 3 [file 42003_2026_10429_MOESM4_ESM.pdf]

nature portfolio

Corresponding author(s): Jixiang Gao

Last updated by author(s): 2025/10/13

Reporting Summary

Nature Portfolio wishes to improve the reproducibility of the work that we publish. This form provides structure for consistency and transparency in reporting. For further information on Nature Portfolio policies, see our Editorial Policies and the Editorial Policy Checklist.

Statistics

For all statistical analyses, confirm that the following items are present in the figure legend, table legend, main text, or Methods section.

n/a	Confirmed	
☐	☒	The exact sample size (n) for each experimental group/condition, given as a discrete number and unit of measurement
☐	☒	A statement on whether measurements were taken from distinct samples or whether the same sample was measured repeatedly
☐	☒	The statistical test(s) used AND whether they are one- or two-sided<br>*Only common tests should be described solely by name; describe more complex techniques in the Methods section.*
☐	☒	A description of all covariates tested
☐	☒	A description of any assumptions or corrections, such as tests of normality and adjustment for multiple comparisons
☒	☐	A full description of the statistical parameters including central tendency (e.g. means) or other basic estimates (e.g. regression coefficient) AND variation (e.g. standard deviation) or associated estimates of uncertainty (e.g. confidence intervals)
☒	☐	For null hypothesis testing, the test statistic (e.g. F, t, r) with confidence intervals, effect sizes, degrees of freedom and P value noted<br>*Give P values as exact values whenever suitable.*
☒	☐	For Bayesian analysis, information on the choice of priors and Markov chain Monte Carlo settings
☒	☐	For hierarchical and complex designs, identification of the appropriate level for tests and full reporting of outcomes
☒	☐	Estimates of effect sizes (e.g. Cohen's d, Pearson's r), indicating how they were calculated

Our web collection on statistics for biologists contains articles on many of the points above.

Software and code

Policy information about availability of computer code

Data collection	Data collection was performed using commercial software including ImageJ (version 1.53t), FlowJo (version 10.8.1), and GraphPad Prism (version 9.5.0). No custom code was used.
Data analysis	Data analysis was conducted using GraphPad Prism (version 9.5.0) for statistical testing and data visualization. No custom code was developed.

For manuscripts utilizing custom algorithms or software that are central to the research but not yet described in published literature, software must be made available to editors and reviewers. We strongly encourage code deposition in a community repository (e.g. GitHub). See the Nature Portfolio guidelines for submitting code & software for further information.

Data

Policy information about availability of data

All manuscripts must include a data availability statement. This statement should provide the following information, where applicable:

- Accession codes, unique identifiers, or web links for publicly available datasets
- A description of any restrictions on data availability
- For clinical datasets or third party data, please ensure that the statement adheres to our policy

Provide your data availability statement here.

Research involving human participants, their data, or biological material

Policy information about studies with human participants or human data. See also policy information about sex, gender (identity/presentation), and sexual orientation and race, ethnicity and racism.

Reporting on sex and gender	Sex and gender data were not collected in this study.
Reporting on race, ethnicity, or other socially relevant groupings	Race or ethnicity information was not collected and was not considered in the analysis.
Population characteristics	Human lung adenocarcinoma tissue samples were obtained from patients diagnosed at Sir Run Run Shaw Hospital. Inclusion criteria included pathologically confirmed LUAD, no prior radiotherapy or chemotherapy before surgery.
Recruitment	Patients were consecutively recruited from Sir Run Run Shaw Hospital at the time of surgical resection. All participants provided written informed consent for the use of their samples in research. No financial incentives were offered.
Ethics oversight	The study protocol was approved by the Ethics Committee of Sir Run Run Shaw Hospital (Ethics number: 2025-2550-01).

Note that full information on the approval of the study protocol must also be provided in the manuscript.

Field-specific reporting

Please select the one below that is the best fit for your research. If you are not sure, read the appropriate sections before making your selection.

☒ Life sciences ☐ Behavioural & social sciences ☐ Ecological, evolutionary & environmental sciences

For a reference copy of the document with all sections, see nature.com/documents/nr-reporting-summary-flat.pdf

Life sciences study design

All studies must disclose on these points even when the disclosure is negative.

Sample size	No statistical methods were used to predetermine sample size. Sample sizes were chosen based on previous studies and are sufficient to detect biologically meaningful differences. Each group included at least three independent biological replicates.
Data exclusions	No data were excluded from the analysis unless there were technical failures (e.g., poor RNA quality or outlier values due to technical errors), which were identified prior to unblinded analysis based on pre-established criteria.
Replication	All key experiments were independently repeated at least three times with consistent results. Details of biological replicates and technical replicates are provided in figure legends or Methods.
Randomization	For in vivo experiments, animals were randomly assigned to experimental groups using a random number generator. For in vitro assays, cells were seeded and treated in a randomized manner across culture plates. For clinical samples, allocation was based on availability and diagnosis, so randomization was not applicable.
Blinding	Blinding was not performed during sample allocation or data collection. However, data analysis was conducted in an unbiased manner using pre-established quantitative criteria. Investigators were not aware of group identities during statistical analysis where applicable.

Reporting for specific materials, systems and methods

We require information from authors about some types of materials, experimental systems and methods used in many studies. Here, indicate whether each material, system or method listed is relevant to your study. If you are not sure if a list item applies to your research, read the appropriate section before selecting a response.

Materials & experimental systems

n/a	Involved in the study	
☐	☒	Antibodies
☐	☒	Eukaryotic cell lines
☒	☐	Palaeontology and archaeology
☐	☒	Animals and other organisms
☒	☐	Clinical data
☒	☐	Dual use research of concern
☒	☐	Plants

Methods

n/a	Involved in the study	
☒	☐	ChIP-seq
☐	☒	Flow cytometry
☒	☐	MRI-based neuroimaging

Antibodies

Antibodies used	USP32 CellSignalingTechnology, #47247), MAPK12 (Invitrogen, PA5-114761), p62 (Abcam, ab155686), LC3B (AAbcam, ab192890), GPX4 (Abcam, ab125066), ACSL4 (CellSignalingTechnology, #38493), FTH1 (CellSignalingTechnology, #4393), and β-actin (CellSignalingTechnology, #4967)
Validation	All antibodies were obtained from commercial suppliers and validated by the manufacturers for use in Western blotting and immunofluorescence in human and mouse samples. Validation information is available on the manufacturers' websites.

Eukaryotic cell lines

Policy information about cell lines and Sex and Gender in Research

Cell line source(s)	LUAD cell lines A459, PC-9, HCI-H1299, HCI-H1703, and the normal human bronchial epithelial cell line 16HBE were obtained from the National Collection of Authenticated Cell Cultures (Shanghai, China). All cell lines were authenticated by the suppliers and used within 10 passages after thawing.
Authentication	Cell line authentication was performed by the supplier using STR profiling, and the cells were used without further authentication in our laboratory.
Mycoplasma contamination	The cell lines were not independently tested for mycoplasma contamination in this study, but were obtained from certified sources that provide mycoplasma-free guarantees.
Commonly misidentified lines (See ICLAC register)	No commonly misidentified or cross-contaminated cell lines listed in the ICLAC database were used in this study.

Animals and other research organisms

Policy information about studies involving animals; ARRIVE guidelines recommended for reporting animal research, and Sex and Gender in Research

Laboratory animals	Male BALB/c nude mice (6–8 weeks old, n = 3 per group) were used for xenograft studies. Stable HCI-H1703 cells with USP32 knockdown or MAPK12 overexpression, and stable A459 cells with MAPK12 knockdown, were subcutaneously injected into the flanks of the mice (5×10^6 cells/mouse).
Wild animals	This study did not involve wild animals.
Reporting on sex	Only male mice were used to avoid potential hormonal variations. Sex was not considered a biological variable in this study.
Field-collected samples	This study did not involve field-collected samples.
Ethics oversight	All animal experiments were approved by the Animal Ethics Committee of Sir Run Run Shaw Hospital and conducted in accordance with institutional guidelines for animal care and use.

Note that full information on the approval of the study protocol must also be provided in the manuscript.

Plants

Seed stocks	This study did not involve any plant materials or seed stocks.
Novel plant genotypes	Not applicable. No transgenic or gene-edited plant lines were used in this study.
Authentication	Not applicable.

Flow Cytometry

Plots

Confirm that:

☒ The axis labels state the marker and fluorochrome used (e.g. CD4-FITC).

☒ The axis scales are clearly visible. Include numbers along axes only for bottom left plot of group (a 'group' is an analysis of identical markers).

☒ All plots are contour plots with outliers or pseudocolor plots.

☒ A numerical value for number of cells or percentage (with statistics) is provided.

Methodology

Sample preparation	Cells were harvested and washed twice with cold PBS. Apoptosis was assessed using the Annexin V–FITC/PI Apoptosis Detection Kit (Beyotime, China) according to the manufacturer's instructions. Briefly, 1×10^5 cells were resuspended in 195 µL binding buffer and stained with 5 µL Annexin V–FITC and 10 µL PI for 15 min at room temperature in the dark. Samples were immediately analyzed by flow cytometry.
Instrument	Flow cytometry was performed on a BD FACSCanto II (BD Biosciences, USA).
Software	Data were acquired using BD FACSDiva software (v9.0) and analyzed with FlowJo (v10.8.1, Tree Star, USA). The same gating parameters were applied to all samples.
Cell population abundance	The percentages of viable (Annexin V^- PI^-), early apoptotic (Annexin V^+ PI^-), late apoptotic (Annexin V^+ PI^+), and necrotic (Annexin V^- PI^+) cells were calculated from the total gated population. Data are presented as mean ± SD from at least three independent experiments.
Gating strategy	Cells were first gated on FSC-A/SSC-A to exclude debris and on FSC-H/FSC-A to remove doublets. Live and dead cell populations were defined based on FITC and PI fluorescence using unstained controls to set quadrant boundaries.

☒ Tick this box to confirm that a figure exemplifying the gating strategy is provided in the Supplementary Information.
