# Supplementary material for: USP32-mediated stabilization of MAPK12 promotes lung adenocarcinoma progression by inhibiting autophagy-ferroptosis
Source: Commun Biol. 2026 Jul 19;9:1505. doi: 10.1038/s42003-026-10429-z (PMC13751611; doi:10.1038/s42003-026-10429-z)
Supplement: Supplementary file 4 [file 42003_2026_10429_MOESM5_ESM.pdf]

nature portfolio

Peer Review File

Stem cells resume asymmetric division upon niche re-entry through reactivating the centrosome orientation checkpoint

Corresponding Author: Dr Mayu Inaba

This file contains all reviewer reports in order by version, followed by all author rebuttals in order by version.

Version 0:

Reviewer comments:

Reviewer #1

(Remarks to the Author)
Bener and colleagues are probing the question of how the pool of spermatogonial stem cells (called germline stem cells or GSCs) in the adult fly testis is maintained. Currently, it is accepted that the attached daughter cell of the GSC (called the gonialblast or Gb) can “swivel” back into the niche and adopt “stemness”. This type of outcome to GSC division is called symmetric renewal, and it was first seen in live imaging by Erica Matunis’ lab (Sheng, 2011). The Matunis lab reported that symmetric renewal happens at a low frequency (like less than 10%) in wild-type testes in steady-state. The Matunis lab developed a method to drive all GSCs into the differentiation program. However, when this stimulus was halted, some differentiating spermatogonia could revert their fate and become GSCs. This process is termed regeneration, and during regeneration, the frequency of symmetric increases substantially (Sheng, 2011). The low baseline rate of symmetric renewal in homeostasis and its increase during stress was recently reported by Kari Lenhart’s lab (Roach, 2024). With this background in mind, Bener and colleagues want to probe the possible contribution of dedifferentiation of early spermatogonia (1- or 2-cell spermatogonia) to the pool of GSCs during normal conditions. The Roach paper stated that they do not observe reversion of these early spermatogonia in steady state but do see dedifferentiation if they stress the flies.

While I think that this is an interesting topic (possible reversion of early spermatogonia), the imaging approaches do not allow Bener and colleagues make strong conclusions. The authors unfortunately do not employ fluorescent markers that allow them to see the ring canals or midbody structures that allow unequivocal identification of clonally-related germ cells (i.e., GSC to its Gb and germ cells with a cyst). Because the authors do not use such tools, they cannot distinguish between a Gb and a 1-cell spermatogonia. Throughout the paper, the cells that are purported to be reverting could actually be Gbs undergoing symmetric renewal. If this is the case (that the authors are actually capturing symmetric renewal), then this work does not advance the field. If the authors want to test their hypothesis, they should repeat these experiments using markers that the Dinardo and Lenhart labs have used in the testis or that Lynn Cooley or Jean-Rene Huyhn use in the ovary, marking the germ cell cortex with one fluorescent probe and marking the midbody with a different fluor. I don’t know if the authors have access to a spinning disc confocal, but this type of microscope really is the best in terms of resolution and length of imaging (up to 24 hours as reported by the Lenhart lab (Roach, 2024)).

The bam-gal4 experiments are not logical and do not support the author's conclusions. Bam-Gal4 labels spermatogonia older (4- and 8-cell spermatogonia) than the ones they are tracking (1- and 2-cell gonia).

The authors state the dedifferentiating cells are already in G2 when they can access to the niche. If they are actually tracking Gbs that are symmetrically renewing (my alternate conclusion), then it is not surprising that the reverting cells are in G2, because G2 is when abscission between the GSC and its Gb occurs (Sheng, 2011; Lenhart, 2015).

Because of the weakness in the approach through this manuscript, alternate conclusions are probable. If the authors want to challenge the current state of the field, then they need to perform live imaging with the right tools.

Reviewer #2

(Remarks to the Author)
The article titled, “Stem cells resume asymmetric division upon niche re-entry”, by Bener et al. describes the behavior of gonialblast (GB) dedifferentiation and links it to the preferential niche attachment of GBs during the G2 phase. The authors

utilized long-term time-lapse imaging ex vivo with a high-sensitivity confocal microscopy technique to monitor the cell division cycles of germline stem cells (GSCs) in Drosophila testes. They expressed GFP-tagged α-tubulin along with a membrane marker, and used cell cycle phase-marking techniques such as Fly-FUCCI to correlate the cell cycle stages of GBs undergoing dedifferentiation. During the study, the authors also described the rates of different types of GSC divisions and their loss from the niche. The data presented in the manuscript is comprehensive and rigorously analyzed using standard statistical methods. The authors further validated their observations using alternative techniques to support the conclusions. The study makes a significant technical advancement by achieving, for the first time, a uniform time-lapse interval of 10 minutes over 16 hours, which enabled effective comparative analysis across different genetic backgrounds. The manuscript is well-written, and the data is effectively organized into six figure panels with adequate illustrations for non-expert readers.

GBs are known to replenish the stem cell pool in both ovaries and testes upon reattachment to the niche, and several previous articles have elaborated on the rates of their recruitment to the niche. Although the cellular behavior of GB reattachment is well-documented, the molecular mechanisms underlying the process remain poorly understood. It is known that Jak-STAT signaling from the niche maintains the stem cell-like properties of GSCs, and their detachment from the niche due to asymmetric divisions is a major cause of GB differentiation. Therefore, controlling spindle orientation through the attachment of centrioles/astral microtubules to the plasma membrane at the appropriate location is critical to this process. The authors found that centrioles in GB cells that reattach to the niche remain unattached for a relatively longer period until the GB contacts the niche. They propose that a centriole checkpoint (COC) mechanism could arrest GBs in the G2 stage to facilitate this process. The authors also hypothesize that GB cells retain the same factors as GSCs, enabling them to revert to stem cell fate. Thus, attachment to the niche emerges as the key driver of this process. The manuscript presents a compelling case for an intriguing new hypothesis.

However, much of the manuscript is written in a descriptive manner, with limited emphasis on the significance of the observations or the mechanistic insights. Major revisions and additions are necessary to highlight key findings and establish their connection to underlying mechanisms, thus qualifying the work as a significant new contribution to the field.

Major Comments:
1. The introduction needs to be rewritten with a clearly focussed motivation and substantive argument justifying the hypothesis. In the current form it lacks the guiding hypothesis and a statement of the problem being pursued.
2. The result sections need to be organized using clearly delineated three step principle: background, observation and conclusion/explanation.
3. Example: The rates of GSC divisions 4.66/16 hours per testis as reported in this manuscript appears to be low considering the estimated 12-14-hour lifespan of GSCs. The authors need to explain the observation.
4. The authors argue that their study establishes dedifferentiation of GB as a regular process. Using nos-GFPtub reporter, they showed that the event occurs at 0.66/16 hour per testis in a steady state condition. In comparison, the replenishment through different types of symmetric division occurs at a rate of (0.13+0.1)/16 hour. The rate of dedifferentiation appeared to increase to 0.85/16 hours using nos-myrGFP and to 1/16 hours in regenerating testes after a transient bam overexpression in GSCs and GBs prior to the observation window. Similarly, the proportions shown in Fig 1F and Fig S2B are different. These data may suggest that the rate at which the phenomenon occurs could be linked to the genetic overexpression backgrounds. The author needs to use an endogenous marker such as Jupiter-GFP protein trap to rule out this possibility.
5. The data also suggest that the asymmetric GSC divisions are most dominant event even after a severe depletion of the GSCs from the niche. In such a scenario, what is the significance of dedifferentiation? A through discussion of the data is needed.
6. The authors imply that GBs that preferentially attach to the niche at G2 stage have a propensity to dedifferentiate. What happens to the other GBs? A cell tracking analysis of the Fly-FUCCI data might throw some useful insight on this process.
7. What are the G2 durations in GBs that attach and those that does not? The data shown in Fig 4A and B is not comprehensible. What does each dot signify and what is the Y-axis? A comparison chart of different cell cycle phases between the GBs that dedifferentiate and the rest that do not would be useful.
8. As per the previous reports the GBs are estimated to have a longer inter-division life-span than GSCs and a comparatively larger proportion of G1 and G2-stage GBs were reported at steady state. The M-phase durations of GSCs and GBs are comparable as per the previous reports. Therefore, comparative analysis of the G1, S and G2 phases amongst the GSCs and GBs would be useful to understand the origin of the dedifferentiation phenomenon. This could also give credence to the COC hypothesis.
9. The authors claim that the COC is activated in GBs due to GSC loss in the ectopic bam expression background is not adequately supported by the data shown in Fig 5. Also, if this is the case, then what happens to the GBs that dedifferentiate at steady state. The observed rates are not much different between these two scenarios.
10. The logic of using bam-Gal4 to knockdown Baz is unclear. Shouldn’t it be done using nos-Gal4 in the GBs to test the requirement?
11. The discussion is disorganized. It merely repeats the observations without mechanistic explanations or implications. Authors need to explain how the observations helped to advance the current understanding and what will be its impact in related filed.
Minor comments:
1. Fig 1F legend is incomprehensible. It should be elaborated to explain the how these two pie charts are obtained. Also, the proportions of dedifferentiation appear to be different in Fig S2B. It should be discussed in the result section.

Reviewer #3

(Remarks to the Author)
In this work, the AUs present a clear description of cellular phenomena taking place in the male germline stem cell (GSC) niche using a long-duration live imaging approach. The AUs report that, as previously published, GSCs can be lost and replenished by symmetrical divisions in adult testes. In addition, the AUs identify a prevalent number of de-differentiation occurrences responsible for nearly 75% of the replenishment events. This novel finding is of intertest to the field and makes the publication of the work worthwhile. However, there are serious issues with the presentation of the results, the writing of the text and the discussion of some of the results. To follow are my comments, some of which are critical for the validity of the conclusions put forward by the AUs.
1- My first criticism is the quality of the written text. I might be wrong, but my feeling is that the MS has been rushed to submission. This is a pity, as the results are interesting and well documented (the quality of some of the movies is outstanding). Reading throughout the MS has taken me a great deal of effort and not much of pleasure. I would suggest the AUs the following:
- To add subheadings in the Results section. This should help conveying the message and, for instance, to discriminate whether the AUs are talking about wild type testes, testes subjected to Bam overexpression or whether they are presenting data related to de-differentiated GB.
- Some sections of the Results and Discussion look like bullet points, lacking contextualization and connection with the rest of the text. See for instance lines 247-270. Most likely, a new reorganization of the Results and Discussion with subheadings will help sorting out this.
- Lines 44-45 and 51-52 are repeated.
- In the text, Table S4 is cited after Table S1, with no mention to Tables S2 or S3 in between. This is confusing.
- The last paragraph of the Results section may belong to the Discussion?
- I find the first paragraph of the Discussion difficult to follow. It starts by talking about GSCs and their predominant asymmetric division. Next, the AUs mention the generality of stem cell loss in tissues as a consequence of different insults or cell senescence and conclude that there must be “additional” mechanisms to ensure stem cell pool maintenance. Additional to what? To asymmetric stem cell divisions? But there are plenty of stem cells that do not divide asymmetrically. I would recommend the AUs to refer only to the case of the GSCs and invoke the need for additional (here you do necessitate other mechanisms to account for the recovery of lost GSCs) mechanisms. Next, they could mention the importance for other stem cell niches known to undergo cell replacement.
2- I am missing the discussion of an important discovery reported in the Results section. First, if one adds up the division events that give rise to new GSCs, they are more frequent than those responsible for the loss of GSCs (Fig. 1F). This would imply that the number of GSCs within the niche increases with time. But this is contrary to what happens in living animals. Thus, is this an artefact of the culturing protocol? This is potentially worrying, as such a conclusion would question the main finding of the work, i.e., the existence of de-differentiation events responsible for a large proportion of GSC replenishment.
3- In the baz RNAi experiments. The AU should show the degree of Baz downregulation. It is difficult to interpret their results without knowing how much baz or Baz is being removed.
4- I would not call 2.29 ± 1.88 hours or 2.1 hours ± 1.72 “immediate” or “promptly” entry into mitosis. There are reintegrated GSCs that require nearly 6-7 hours to divide. This is nearly 50% of the time the AUs claim it takes a GSC to divide.
5- Line 124: In legend to Table 3 there is reference to the de-differentiation of a 4-cell SG.
6- Legend to Fig. 1F). How is the “average distribution of divisions” calculated?
7- Table S2: What does “no division” mean in the Symmetric renewal category?
8- The legend of Figure 4 and the diagram 4C indicates “Note that Drosophila GSCs do not have a detectable G1 phase". This is at odds with other literature that has detected GSCs at G1 phase (“green” and “black” cells). Moreover, the example provided by the authors shows two non-mitotic green GSCs for the whole duration of the recording (on top of the hub). In addition, the live imaging strategy used by the authors does not allow to detect “black” cells (end of G1) due to the lack of a counterstaining for all germline cells – this limitation should be acknowledged and the “lack of G1 phase” statements reviewed.
9- Movies S6 and S7 depict cases of symmetric divisions with a GSC loss result. S7, however, just shows the loss of a GSC without its previous division. A better example should be provided, including its previous division.
10- Figure 2 depicts a dedifferentiation case both as raw images and a 3D reconstruction. The number of frames of both depictions are different and the tiles for the same timing do not seem to match between them.
11- Figure S5 shows quantifications of GSCs, GB and SGs numbers. These results are not mentioned or discussed in the text.
12- bam-Gal4 is used to drive Baz-RNAi (Fig. 5). This Gal4 is only expressed in 8- and 16-cell cyst stages. As the manuscript focuses on dedifferentiation events of earlier spermatogonia stages, this should be properly discussed, as its modifies the interpretation of the results.

Version 1:

Reviewer comments:

Reviewer #1

(Remarks to the Author)
REVIEWER 1 ORIGINAL COMMENTS TO AUTHORS: Bener and colleagues are probing the question of how the pool of spermatogonial stem cells (called germline stem cells or GSCs) in the adult fly testis is maintained. Currently, it is accepted that the attached daughter cell of the GSC (called the gonialblast or Gb) can “swivel” back into the niche and adopt “stemness”. This type of outcome to GSC division is called symmetric renewal, and it was first seen in live imaging by Erica

Matunis' lab (Sheng, 2011). The Matunis lab reported that symmetric renewal happens at a low frequency (like less than 10%) in wild-type testes in steady-state. The Matunis lab developed a method to drive all GSCs into the differentiation program. However, when this stimulus was halted, some differentiating spermatogonia could revert their fate and become GSCs. This process is termed regeneration, and during regeneration, the frequency of symmetric increases substantially (Sheng, 2011). The low baseline rate of symmetric renewal in homeostasis and its increase during stress was recently reported by Kari Lenhart's lab (Roach, 2024). With this background in mind, Bener and colleagues want to probe the possible contribution of dedifferentiation of early spermatogonia (1- or 2-cell spermatogonia) to the pool of GSCs during normal conditions. The Roach paper stated that they do not observe reversion of these early spermatogonia in steady state but do see dedifferentiation if they stress the flies.
While I think that this is an interesting topic (possible reversion of early spermatogonia), the imaging approaches do not allow Bener and colleagues make strong conclusions. The authors unfortunately do not employ fluorescent markers that allow them to see the ring canals or midbody structures that allow unequivocal identification of clonally-related germ cells (i.e., GSC to its Gb and germ cells with a cyst). Because the authors do not use such tools, they cannot distinguish between a Gb and a 1-cell spermatogonia. Throughout the paper, the cells that are purported to be reverting could actually be Gbs undergoing symmetric renewal. If this is the case (that the authors are actually capturing symmetric renewal), then this work does not advance the field. If the authors want to test their hypothesis, they should repeat these experiments using markers that the Dinardo and Lenhart labs have used in the testis or that Lynn Cooley or Jean-Rene Huyhn use in the ovary, marking the germ cell cortex with one fluorescent probe and marking the midbody with a different fluor. I don't know if the authors have access to a spinning disc confocal, but this type of microscope really is the best in terms of resolution and length of imaging (up to 24 hours as reported by the Lenhart lab (Roach, 2024).
We thank the reviewer for bringing this critical point to our attention. We fully agree that it is essential to align our results with those of previous studies. The main point is whether dedifferentiation occurs from single GBs (post-cytokinetic) rather than from pre-cytokinetic GBs in steady-state conditions.

AUTHOR RESPONSE: We have revisited the papers noted by the reviewer and their methodologies and further conducted experiments using the genotypes used in these studies. Importantly, we consistently observed the dedifferentiation event from post-cytokinetic GBs in all genotypes. As noted by this reviewer, this type of event has never been reported in the past. We realized that there are differences in definitions or techniques among studies. We first want to clarify regarding the definitions and markers used in past studies:

- Roach, 2024 used the genotype: P{nos::eGFP-Moe::nos 3'UTR} with sqh-mcherry that can visualize the cell cortex and the midbody, respectively. They used a different definition than we did in our study for the events, as they do not distinguish between post-cytokinetic GB (which we think is the main contributor for dedifferentiation) and pre-cytokinetic GB, still interconnected with GSCs.
- In contrast to Roach et al., an older study Sheng, 2011 separately defined events occurring in post-cytokinetic GBs vs. pre-cytokinetic GBs. They used the genotype: nosGal4>UAS-GMA (the actin-binding domain of moesin tagged with GFP) or P{nos::eGFP-Moe::nos 3'UTR}. However, this study only used 2D imaging.

dedifferentiation events are almost only detectable after 3D monitoring of the image, which we always do with short-interval z-stacks(~1.5µm).

We found that the frequency of "dedifferentiation" events ranges from 5% to 17% (~10% on average). The sample number is still low for some genotypes, which led to high variation in the rates of events. However, dedifferentiation events originating from post-cytokinetic GBs are consistently observed in all genotypes. We also want to emphasize that we conducted a total of 1372 hours of recording of 3D niches as summarized in the table below. In contrast, older studies conducted less imaging, which may also explain the difference (Roach et al paper analyzed a total of ~792 hours; Sheng et al analyzed a total of 451 hours). We now discuss the potential reasons for discrepancies and clearly state the definition and frequencies of different genotypes in the revised result section associated with Figure 1A.

REVIEWER 1 RESPONSE TO REBUTTAL: There is a SINGLE movie with spaghetti-squash mCherry in the revised paper (Movie S14). The midbody is not apparent in the purported Gb gaining access to the niche. Even in the stills of this movie (Fig. S3B), I cannot see the midbody between the purported GSC-Gb pair. I am not convinced that these two germ cells are clonally related. As such, I am not confident in GSC-division outcomes listed in Figure 1A for nos-myrGFP plus Sqh-mCherry. As a result, I am not convinced of the author's conclusions and other conclusions are possible/probable.

The fundamental problem with their imaging approach still exists – most of the imaging is performed with stocks that do not mark the midbody and as I have written above, their live imaging with spaghetti-squash mCherry is not presented in a convincing manner. If the authors want an example of how GSC-Gb living imaging with a midbody marker should be presented, I refer them to Lenhart (PMID: 26143993) "Movie S1. Mitosis through Abscission in a Wild-Type GSC Reveals Two Phases of Abscission Delay" and the stills for this movie in Fig. 1E1-E6. I also have strong reservations about emphasising the greater number of hours of their imaging than the Lenhart paper when the most of Bener et al live imaging did not have a midbody marker. Unless the live imaging methodology includes a midbody, it is impossible to prove that a GSC is attached to a Gb. Imaging midbodies has been instrumental in determining clonal relationships between female germline stem cells (GSCs) (Mathieu PMID: 35587967), male GSCs (Lenhart PMID: 26143993), male somatic cells in the testis (Price PMID: 36898376), and feminized somatic cells of the testis (Roach PMID: 40600835) to name a few pertinent examples. Without labeling the midbody, Inabu and colleagues cannot distinguish between a Gb and a 1-cell spermatogonia, despite the new analyses presented in the rebuttal. As such, the new analyses written in this rebuttal are not scientifically robust and other interpretations are possible or probable.

I also take issue with the new nomenclature "pre-cytokinetic Gb and post-cytokinetic Gb". The field does not use these terms.

A Gb is only a Gb when it is attached to a GSC. After abscission, it is a 1-cell spermatogonia.

REVIEWER 1 ORIGINAL COMMENT: The bam-gal4 experiments are not logical and do not support the author's conclusions. Bam-Gal4 labels spermatogonia older (4- and 8-cell spermatogonia) than the ones they are tracking (1- and 2-cell gonia).

AUTHOR RESPONSE: All reviewers raised the same concern, and we agree with this point. We have conducted the same experiment now with the germline nosGal4 driver, which highly expressed at in the 1-2 cell stage. Using nosGal4, we observed the same dedifferentiation defect, now shown in Figure 4F.

REVIEWER 1 ORIGINAL COMMENT: The authors state the dedifferentiating cells are already in G2 when they can access to the niche. If they are actually tracking Gbs that are symmetrically renewing (my alternate conclusion), then it is not surprising that the reverting cells are in G2, because G2 is when abscission between the GSC and its Gb occurs (Sheng, 2011; Lenhart, 2015).

AUTHOR RESPONSE: We were also concerned that the biased cell cycle phase was merely a coincidence, given that post-cytokinetic GBs are all in the G2 phase, which we also agree would suggest that we are certainly detecting "post-cytokinetic" cells. We had carefully analyzed in the original submitted manuscript the time-to-mitosis, and compared it with control GSCs.

If we assume that dedifferentiation can randomly occur during any phase of the cell cycle, we would expect the time between reattachment and the 1st mitotic entry (see Methods for defining the reattachment point) to be randomly distributed across ~14 hours, the cycle length of male GSCs as estimated in previous works. We observed that the mitotic entry of native GSCs already present in the niche at the start of imaging showed random distribution throughout the time of imaging periods (the average time to mitosis was 7.61 ± 4.39 hours n=128 in the steady-state phase, 6.8 ± 4.16 hours, n=59, in the regeneration phase) (Figure 3A-B, Table S1-2). In contrast, the average time between hub reattachment and the 1st mitotic entry of dedifferentiated GSCs was drastically short in most of observed "dedifferentiation" cases (2.29 ± 1.88 hours, n = 16 in the steady-state, 2.1 hours ± 1.72, n = 21 in the regeneration) (Figure 3A-B, Table S1-2).
Based on these data, we were able to conclude that the cell cycle stage is in the "late-G2" phase, rather than being randomly distributed throughout the entire G2 phase (Figure 3A, B). This led us to hypothesize that the cells are possibly arrested in the centrosome orientation checkpoint and together with our imaging data, rule out the alternative interpretation that these are symmetrically renewing GBs.

REVIEWER 1 RESPONSE TO REBUTTAL: Again because of the flawed methodology in the live imaging, the authors cannot prove that that are studying symmetrically renewing Gbs or 1-cell spermatogonia and this additional analysis is not meaningful. Because of the weakness in the approach through this manuscript, alternate conclusions are probable. If the authors want to challenge the current state of the field, then they need to perform live imaging with the right tools.

We hope that our additional data using previous genotypes and carefully focused on the source of the cell origin have demonstrated the consistency in our overall observations. We hope this now suffices to address the reviewer's concern.

REVIEWER 1 RESPONSE TO REBUTTAL: I am still not satisfied with the methodology used by the authors. If they want to test their hypotheses in a robust manner, they will need to repeat all their live imaging with a midbody marker.

Reviewer #2

(Remarks to the Author)
The article titled, "Stem cells resume asymmetric division upon niche re-entry through reactivating the centrosome orientation checkpoint" was originally submitted with the title "Stem cells resume asymmetric division upon niche re-entry", by Bener et al. It describes the behavior of gonialblast (GB) dedifferentiation and links it to the preferential niche attachment of GBs during the G2 phase. The authors utilized long-term time-lapse imaging ex vivo with a high-sensitivity confocal microscopy technique to monitor the cell division cycles of germline stem cells (GSCs) in Drosophila testes. They expressed GFP-tagged α-tubulin along with a membrane marker and used cell cycle phase marking techniques such as Fly-FUCCI to correlate the cell cycle stages of GBs undergoing dedifferentiation. During the study, the authors also described the rates of different types of GSC divisions and their loss from the niche.
In the revised version, the authors included a thorough reanalysis of the data, which help them to amend some of the conclusions. They have also included a new experimental data using nosGal4 driver as per the suggestions of the previous review. The data presented in the manuscript is comprehensive and rigorously analyzed using standard statistical methods. The authors further validated their observations using alternative techniques to support the conclusions. The study makes a significant technical advancement by achieving, for the first time, a uniform time-lapse interval of 10 minutes over 16 hours, which enabled effective comparative analysis across different genetic backgrounds. The manuscript is now rewritten with a better flow and several portions are now modified to conform with the earlier review comments. The data is effectively organized into six figure panels with adequate illustrations for non-expert readers.
I have earlier opined that GB dedifferentiation is critical for stem cell maintenance in both ovaries and testes and it happens through the reattachment of some of the GBs. However, it is still unclear what determined this reattachment. The authors found that a delayed centriole dynamics and membrane association could trigger a COC checkpoint and arrest the GBs in the G2 phase, facilitating the back transition. They propose that a centriole checkpoint (COC) mechanism could arrest GBs in the G2 stage to facilitate this process. The authors also hypothesize that GB cells retain the same factors as GSCs, enabling them to revert to stem cell fate. Thus, attachment to the niche emerges as the key driver of this process. The revised

manuscript now presents a compelling case for an intriguing new hypothesis.
I find the manuscript is now well composed with clearly constructed hypothesis and the results are discussed with a correlated hypothesis. The conclusions are justified and well organized. Therefore, I have no major comment against the results section of the manuscript.
However, the discussion section is still weak. It merely summarizes the observations without clear interpretations. It will useful if the authors link the key observations of the study to other systems and discuss how their observation may open a new proposal and how they are distinct from the previously published data. For example, the data about the proposed role of COC needs to be discussed with the insipient merits and demerits of the data. In some sense the authors need to provide a balanced critique of their experimental observations. Also, the discussion needs to be structured according to a theme. For instance, the key issue examined in the study is how the apparently differentiated GBs regain their stem-cell like property. One possibility demonstrated by the study could suggest an invocation of COC due to delayed attachment of the centriole. How strong or weak is the evidence? Also, how is this attachment could be delayed? Author can discuss these issues in a structured manner to provide a future roadmap based on their observations.

Reviewer #3

(Remarks to the Author)
The authors have answered satisfactorily all of my concerns.

Reviewer #4

(Remarks to the Author)
Bener et al.'s paper demonstrated that dedifferentiation occurs from “single” gonialblast cells (Gbs) under both physiological and regenerative conditions. Furthermore, their research provides a mechanistic perspective on the dedifferentiation process; it explicitly clarifies that this form of dedifferentiation is different from that of GBSs, which remain connected to GSCs after GSC division—a phenomenon previously documented and called symmetric renewal. As a result, I consider Bener et al.'s manuscript suitable for publication in Communications Biology as a novel discovery.

Although this is a minor point, I agree with a criticism from reviewer #1, who claims that conducting live imaging with specific cellular bridge markers between germline stem cells (GSCs) and gonialblasts (GBs), such as midbody ring (Pavarotti) and/or adducin, would improve the clarity of the findings. Presenting live imaging results would better illustrate the authors’ assertion that dedifferentiation occurs from “single” GBs, if possible. Even if it is technically difficult, I believe the paper’s conclusions would remain unaffected, since Bener et al. clearly showed that “single” GBs undergo dedifferentiation in their Figures 1D and 3C.

Version 2:

Reviewer comments:

Reviewer #2

(Remarks to the Author)
The manuscript titled, "Stem cells resume asymmetric division upon niche re-entry through reactivating the centrosome orientation checkpoint," by Baner et al., described a new phenomenon of germ cell dedifferentiation form the G2 arrested GBs. This constituted description of a novel phenomenon and warrants publication on Communication biology. The authors have revised the manuscript with new data addressing the concerns of Reviewers 1 and 4. They have also rewritten the discussion section as per the suggestion of Reviewer 2. Reviewer 3 was satisfied with the previous revision of the manuscript. Therefore, based on the current revision, I am of the opinion that the manuscript is now ready for acceptance.

Reviewer #4

(Remarks to the Author)
With regard to the points I proposed during the previous review cycle, the author has revised the images and integrated them into Fig. S3B-D. Accordingly, I have no additional comments concerning those points.

Nonetheless, I believe the discussion section requires further restructuring. Firstly, the initial and second paragraphs are superfluous, as they reiterate information from the introduction. Secondly, the authors should commence each paragraph with their findings from this study to facilitate a clearer discussion of the results. This approach would enhance the clarity of the flow and the overall presentation of the discussion points.

Open Access This Peer Review File is licensed under a Creative Commons Attribution 4.0 International License, which permits use, sharing, adaptation, distribution and reproduction in any medium or format, as long as you give appropriate credit to the original author(s) and the source, provide a link to the Creative Commons license, and indicate if changes were made.
In cases where reviewers are anonymous, credit should be given to 'Anonymous Referee' and the source.
The images or other third party material in this Peer Review File are included in the article's Creative Commons license, unless indicated otherwise in a credit line to the material. If material is not included in the article's Creative Commons license and your intended use is not permitted by statutory regulation or exceeds the permitted use, you will need to obtain permission directly from the copyright holder.
To view a copy of this license, visit https://creativecommons.org/licenses/by/4.0/

First of all, we thank editor and the reviewers for giving us critical suggestions on our original manuscripts. After several months of extensive work, we have addressed essentially all concerns and suggestions raised by reviewers. Therefore, we would like to request transferring this version to "Cell Death and Disease".

Below is a response to reviewers' comments.

Reviewer #1 (Remarks to the Author):

Bener and colleagues are probing the question of how the pool of spermatogonial stem cells (called germline stem cells or GSCs) in the adult fly testis is maintained. Currently, it is accepted that the attached daughter cell of the GSC (called the gonialblast or Gb) can "swivel" back into the niche and adopt "stemness". This type of outcome to GSC division is called symmetric renewal, and it was first seen in live imaging by Erica Matunis' lab (Sheng, 2011). The Matunis lab reported that symmetric renewal happens at a low frequency (like less than 10%) in wild-type testes in steady-state. The Matunis lab developed a method to drive all GSCs into the differentiation program. However, when this stimulus was halted, some differentiating spermatogonia could revert their fate and become GSCs. This process is termed **regeneration**, and during regeneration, the frequency of symmetric increases substantially (Sheng, 2011). The low baseline rate of symmetric renewal in homeostasis and its increase during stress was recently reported by Kari Lenhart's lab (Roach, 2024). With this background in mind, Bener and colleagues want to probe the possible contribution of dedifferentiation of early spermatogonia (1- or 2-cell spermatogonia) to the pool of GSCs during normal conditions. The Roach paper stated that they do not observe **reversion** of these early spermatogonia in steady state but do see dedifferentiation if they stress the flies.

While I think that this is an interesting topic (possible reversion of early spermatogonia), the imaging approaches do not allow Bener and colleagues make strong conclusions. The authors unfortunately do not employ fluorescent markers that allow them to see the ring canals or midbody structures that allow unequivocal identification of clonally-related germ cells (i.e., GSC to its Gb and germ cells with a cyst). Because the authors do not use such tools, they cannot distinguish between a Gb and a 1-cell spermatogonia. Throughout the paper, the cells that are purported to be reverting could actually be Gbs undergoing symmetric renewal. If this is the case (that the authors are actually capturing symmetric renewal), then this work does not advance the field. If the authors want to test their hypothesis, they should repeat these experiments using markers that the Dinardo and Lenhart labs have used in the testis or that Lynn Cooley or Jean-Rene Huyhn use in the ovary, marking the germ cell cortex with one fluorescent probe and marking the midbody

with a different fluor. I don't know if the authors have access to a spinning disc confocal, but this type of microscope really is the best in terms of resolution and length of imaging (up to 24 hours as reported by the Lenhart lab (Roach, 2024).

We thank the reviewer for bringing this critical point to our attention. We fully agree that it is essential to align our results with those of previous studies. The main point is whether dedifferentiation occurs from single GBs (post-cytokinetic) rather than from pre-cytokinetic GBs in steady-state conditions.

We have revisited the papers noted by the reviewer and their methodologies and further conducted experiments using the genotypes used in these studies. Importantly, we consistently observed the dedifferentiation event from post-cytokinetic GBs in all genotypes. As noted by this reviewer, this type of event has never been reported in the past. We realized that there are differences in definitions or techniques among studies. We first want to clarify regarding the definitions and markers used in past studies:

- Roach, 2024 used the genotype: P{nos::eGFP-Moe::nos 3'UTR} with sqh-mcherry that can visualize the cell cortex and the midbody, respectively. They used a different definition than we did in our study for the events, as they do not distinguish between post-cytokinetic GB (which we think is the main contributor for dedifferentiation) and pre-cytokinetic GB, still interconnected with GSCs.
- In contrast to Roach et al., an older study Sheng, 2011 separately defined events occurring in post-cytokinetic GBs vs. pre-cytokinetic GBs. They used the genotype: nosGal4>UAS-GMA (the actin-binding domain of moesin tagged with GFP) or P{nos::eGFP-Moe::nos 3'UTR}. However, this study only used 2D imaging. dedifferentiation events are almost only detectable after 3D monitoring of the image, which we always do with short-interval z-stacks(~1.5μm).

We found that the frequency of "dedifferentiation" events ranges from 5% to 17% (~10% on average). The sample number is still low for some genotypes, which led to high variation in the rates of events. However, dedifferentiation events originating from post-cytokinetic GBs are consistently observed in all genotypes. We also want to emphasize that we conducted a total of 1372 hours of recording of 3D niches as summarized in the table below. In contrast, older studies conducted less imaging, which may also explain the difference (Roach et al paper analyzed a total of ~792 hours; Sheng et al analyzed a total of 451 hours). We now discuss the potential reasons for discrepancies and clearly state the definition and frequencies of different genotypes in the revised result section associated with Figure 1A.

Genotypes	nosGal4> GFP-αTub	nosGal4> UAS-myrGFP	nos-myrGFP	nos-myrGFP plus Sqh-mCherry	nosGal4 > UAS-GMA	Sum	Frequency
Asymmetric division	140	110	64	43	52	409	71.3%
Symmetric renewal	7	5	7	5	4	28	4.9%
Dedifferentiation	20	9	18	3	7	57	9.9%
Symmetric differentiation	12	11	8	3	9	43	7.5%
Loss	8	12	6	6	5	37	6.4%
Sum	187	147	103	60	77	574	
# of scored testis	30	26	21	11	10	98	
**Dedifferentiation from single GB**	**10.7%**	**6.1%**	**17.5%**	**5.0%**	**9.1%**		

This table shows the number of events observed in all genotypes used in this study. Same table has been inserted in New Figure 1A

The bam-gal4 experiments are not logical and do not support the author's conclusions. Bam-Gal4 labels spermatogonia older (4- and 8-cell spermatogonia) than the ones they are tracking (1- and 2-cell gonia).

All reviewers raised the same concern, and we agree with this point. We have conducted the same experiment now with the germline nosGal4 driver, which highly expressed at in the 1-2 cell stage. Using nosGal4, we observed the same dedifferentiation defect, now shown in Figure 4F.

The authors state the dedifferentiating cells are already in G2 when they can access to the niche. If they are actually tracking Gbs that are symmetrically renewing (my alternate conclusion), then it is not surprising that the reverting cells are in G2, because G2 is when abscission between the GSC and its Gb occurs (Sheng, 2011; Lenhart, 2015).

We were also concerned that the biased cell cycle phase was merely a coincidence, given that post-cytokinetic GBs are all in the G2 phase, which we also agree would suggest that we are certainly detecting “post-cytokinetic” cells. We had carefully analyzed in the original submitted manuscript the time-to-mitosis, and compared it with control GSCs.

If we assume that dedifferentiation can randomly occur during any phase of the cell cycle, we would expect the time between reattachment and the 1st mitotic entry (see Methods for defining the reattachment point) to be randomly distributed across ~14 hours, the cycle length of male GSCs as estimated in previous works. We observed that the mitotic entry of native GSCs already present in the niche at the start of imaging showed random distribution throughout the time of imaging periods (the average time to mitosis was 7.61 ± 4.39 hours n=128 in the steady-state phase, 6.8 ± 4.16 hours, n=59, in the regeneration phase) (Figure 3A-B, Table S1-2). In contrast, the average time between hub reattachment and the 1st mitotic entry of dedifferentiated GSCs was drastically short in most of observed “dedifferentiation” cases (2.29 ± 1.88 hours, n = 16 in the steady-state, 2.1 hours ± 1.72, n = 21 in the regeneration) (Figure 3A-B, Table S1-2).

Based on these data, we were able to conclude that the cell cycle stage is in the "late-G2" phase, rather than being randomly distributed throughout the entire G2 phase (Figure 3A, B). This led us to hypothesize that the cells are possibly arrested in the centrosome orientation checkpoint and together with our imaging data, rule out the alternative interpretation that these are symmetrically renewing GBs.

Because of the weakness in the approach through this manuscript, alternate conclusions are probable. If the authors want to challenge the current state of the field, then they need to perform live imaging with the right tools.

We hope that our additional data using previous genotypes and carefully focused on the source of the cell origin have demonstrated the consistency in our overall observations. We hope this now suffices to address the reviewer's concern.

Reviewer #2 (Remarks to the Author):

The article titled, "Stem cells resume asymmetric division upon niche re-entry", by Bener et al. describes the behavior of gonialblast (GB) dedifferentiation and links it to the preferential niche attachment of GBs during the G2 phase. The authors utilized long-term time-lapse imaging ex vivo with a high-sensitivity confocal microscopy technique to monitor the cell division cycles of germline stem cells (GSCs) in Drosophila testes. They expressed GFP-tagged α-tubulin along with a membrane marker, and used cell cycle phase-marking techniques such as Fly-FUCCI to correlate the cell cycle stages of GBs undergoing dedifferentiation. During the study, the authors also described the rates of different types of GSC divisions and their loss from the niche. The data presented in the manuscript is comprehensive and rigorously analyzed using standard statistical methods. The authors further validated their observations using alternative techniques to support the conclusions. The study makes a significant technical advancement by achieving, for the first time, a uniform time-lapse interval of 10 minutes over 16 hours, which enabled effective comparative analysis across different genetic backgrounds. The manuscript is well-written, and the data is effectively organized into six figure panels with adequate illustrations for non-expert readers.

GBs are known to replenish the stem cell pool in both ovaries and testes upon reattachment to the niche, and several previous articles have elaborated on the rates of their recruitment to the niche. Although the cellular behavior of GB reattachment is well-documented, the molecular mechanisms underlying the process remain poorly understood. It is known that Jak-STAT signaling from the niche maintains the stem cell-like properties of

GSCs, and their detachment from the niche due to asymmetric divisions is a major cause of GB differentiation. Therefore, controlling spindle orientation through the attachment of centrioles/astral microtubules to the plasma membrane at the appropriate location is critical to this process. The authors found that centrioles in GB cells that reattach to the niche remain unattached for a relatively longer period until the GB contacts the niche. They propose that a centriole checkpoint (COC) mechanism could arrest GBs in the G2 stage to facilitate this process. The authors also hypothesize that GB cells retain the same factors as GSCs, enabling them to revert to stem cell fate. Thus, attachment to the niche emerges as the key driver of this process. The manuscript presents a compelling case for an intriguing new hypothesis.

However, much of the manuscript is written in a descriptive manner, with limited emphasis on the significance of the observations or the mechanistic insights. Major revisions and additions are necessary to highlight key findings and establish their connection to underlying mechanisms, thus qualifying the work as a significant new contribution to the field.

Major Comments:

1. The introduction needs to be rewritten with a clearly focussed motivation and substantive argument justifying the hypothesis. In the current form it lacks the guiding hypothesis and a statement of the problem being pursued.

2. The result sections need to be organized using clearly delineated three step principle: background, observation and conclusion/explanation.

3. Example: The rates of GSC divisions 4.66/16 hours per testis as reported in this manuscript appears to be low considering the estimated 12-14-hour lifespan of GSCs. The authors need to explain the observation.

We appreciate the broader point raised by the reviewer regarding the organization and clarity of the paper. We have revised the paper with an eye towards better explaining the questions and the basis for conclusions throughout. For the specific example raised by Reviewer 2 regarding the observation of rate of GSC divisions, in revisiting our imaging data analysis, we realized that we had made an error in our statement. The approximate number of the GSCs covered in our entire z-stacks was 5 in average, meaning that we were consistently missing about 3~4 GSCs from all imaging. Therefore, the estimated cell cycle duration from our data is actually 14.29 hours (1372 total hours recorded for 5 GSCs, and total mitosis observed was 480), which is consistent with previously reported hours. We have added this description to the revised manuscript.

4. The authors argue that their study establishes dedifferentiation of GB as a regular process. Using nos-GFPtub reporter, they showed that the event occurs at 0.66/16 hour per testis in a steady state condition. In comparison, the replenishment through different types of symmetric division occurs at a rate of (0.13+0.1)/16 hour. The rate of dedifferentiation appeared to increase to 0.85/16 hours using nos-myrGFP and to 1/16 hours in regenerating testes after a transient bam overexpression in GSCs and GBs prior to the observation window. Similarly, the proportions shown in Fig 1F and Fig S2B are different. These data may suggest that the rate at which the phenomenon occurs could be linked to the genetic overexpression backgrounds. The author needs to use an endogenous marker such as Jupiter-GFP protein trap to rule out this possibility.

We appreciate this important point raised by the reviewer. There is variation across different genotypes, which we have now updated to show in a table (New Figure 1A, inserted below). However, we were unable to find an endogenous marker that can distinguish between GSC and somatic cells, both of which are located around the hub. We generated myr-GFP directly driven by the nanos promoter (instead of using the Gal4-UAS system), which can be the closest option to physiological conditions. We now provide a comparison of the multiple markers used in the field and discuss the limitations of this method in the Results section and in the Discussion.

Genotypes	nosGal4> GFP-αTub	nosGal4> UAS-myrGFP	nos-myrGFP	nos-myrGFP plus Sqh-mCherry	nosGal4 > UAS-GMA	Sum	Frequency
Asymmetric division	140	110	64	43	52	409	71.3%
Symmetric renewal	7	5	7	5	4	28	4.9%
Dedifferentiation	20	9	18	3	7	57	9.9%
Symmetric differentiation	12	11	8	3	9	43	7.5%
Loss	8	12	6	6	5	37	6.4%
Sum	187	147	103	60	77	574	
# of scored testis	30	26	21	11	10	98	
**Dedifferentiation from single GB**	**10.7%**	**6.1%**	**17.5%**	**5.0%**	**9.1%**		

This table shows the number of events observed in all genotypes used in this study. Same table has been inserted in New Figure 1A

5. The data also suggest that the asymmetric GSC divisions are most dominant event even after a severe depletion of the GSCs from the niche. In such a scenario, what is the significance of dedifferentiation? A through discussion of the data is needed.

Thank you for raising this important point. Because asymmetric division is not capable of increasing or decreasing the stem cell number, either symmetric divisions or dedifferentiation are indispensable for replenishing the lost stem cells. However, whether dedifferentiation is specifically required for maintaining stem cells remains unknown. Interestingly, our previous study demonstrated that enhancing dedifferentiation significantly increases the number of GSCs in aged flies (Ridwan et al., 2024). This suggests that the age-related decline of the stem cell number is dependent on the suppression of

the dedifferentiation mechanism. Future studies to determine genes specifically required for this process will contribute to understanding the biological significance of dedifferentiation.

We have added this point to our discussion section.

6. The authors imply that GBs that preferentially attach to the niche at G2 stage have a propensity to dedifferentiate. What happens to the other GBs? A cell tracking analysis of the Fly-FUCCI data might throw some useful insight on this process.

Thank you for this important suggestion. Due to the difficulty of using live imaging to trace GB populations (due to its highly mobile feature), we took an alternative approach. In Figure4 C-E, we have shown that COC activated GBs are accumulating near the niche in the regenerative condition compared to steady-state condition. It is a fascinating idea but still unclear whether these GBs are selected and predetermined for dedifferentiation or randomly chosen from the population. We have added this point to the discussion section.

7. What are the G2 durations in GBs that attach and those that does not? The data shown in Fig 4A and B is not comprehensible. What does each dot signify and what is the Y-axis?

Each dot represents one traced cell in long-term live imaging, either the cell undergone dedifferentiation event (dedifferentiated GSCs) or stayed in a niche as a GSC throughout the imaging session (native GSCs). They are plotted to the graph based on the time taken between niche-reattachment and following mitotic entry. For the native GSCs (control), times toward mitotic entry from start of the imaging were plotted, which showed random distribution. Y-axis does not represent any value. Upper half shows native GSCs, lower half shows dedifferentiated GSCs. We have revised the figure legend and result section to better clarify these figures.

A comparison chart of different cell cycle phases between the GBs that dedifferentiate and the rest that do not would be useful.

8. As per the previous reports the GBs are estimated to have a longer inter-division life-span than GSCs and a comparatively larger proportion of G1 and G2-stage GBs were reported at steady state. The M-phase durations of GSCs and GBs are comparable as per the previous reports. Therefore, comparative analysis of the G1, S and G2 phases amongst the GSCs and GBs would be useful to understand the origin of the dedifferentiation phenomenon. This could also give credence to the COC hypothesis.

Thank you for raising these important points. We completely agree that Figure 4A, B (current Figure 3A, B) is more legitimate to trace the rest of the GBs as the control if

majority of dedifferentiation occurs from GB as we suggest. However, it was technically very difficult to follow GBs throughout the imaging periods because they are not tethered to the hub. Based on the previous study, GBs likely have similar rate of cell cycle to GSCs' (Gadre et al, 2021, cited in the revised text). Therefore, we used the native GSCs as the control instead of GBs. We added this point in the revised result section.

9. The authors claim that the COC is activated in GBs due to GSC loss in the ectopic bam expression background is not adequately supported by the data shown in Fig 5. Also, if this is the case, then what happens to the GBs that dedifferentiate at steady state. The observed rates are not much different between these two scenarios.

We realized that we did not interpret the data sufficiently in the original manuscript. As pointed out by this reviewer, the Rate of dedifferentiation was increased only about 1.5~2 times in the regenerative condition (Figure 2B). Therefore, COC-active GB populations are likely present in steady-state testes. However, because the Colcemid treatment method cannot directly label the COC-arrested cells, and we can only label part of the non-COC cells, it is difficult to assess exact number of COC active cells in both conditions. In Figure 4E, we show ~40% PH3 positive GBs (partially labeled nonCOC cells) are becoming ~20%. This suggests the increase of the population size of GBs that are activated for COC and competent to dedifferentiation. We have explained details of this interpretation in the revised result section, which reads as follows:

"We scored approximately ~40% PH3 positive GBs are reducing to ~20%. This suggests the emergence of the population of GBs that are activated for COC and competent to dedifferentiation. Although it is difficult to estimate exact frequency of the COC-arrested cells due to the ambiguity of the labeling method, the data indicate that the COC is likely reactivated in these cell populations located outside of the niche"

10. The logic of using bam-Gal4 to knockdown Baz is unclear. Shouldn't it be done using nos-Gal4 in the GBs to test the requirement?

Same concern was raised by all reviewer and we completely agree with this point. We have conducted the same experiment now with the germline nosGal4 driver, which highly expressed at in the 1-2 cell stage. Using nosGal4, we observed the same dedifferentiation defect, now shown in Figure 4F.

11. The discussion is disorganized. It merely repeats the observations without mechanistic explanations or implications. Authors need to explain how the observations helped to advance the current understanding and what will be its impact in related filed.

Thank you for the suggestion. We revised the discussion section to provide a comprehensive description of the potential impact of this study.

Minor comments:

1. Fig 1F legend is incomprehensible. It should be elaborated to explain the how these two pie charts are obtained. Also, the proportions of dedifferentiation appear to be different in Fig S2B. It should be discussed in the result section.

We apologize the pie charts shown in old Fig 1F are confusing.

We have changed this data presentation to new Figure1A table to avoid complication.

Reviewer #3 (Remarks to the Author):

In this work, the AUs present a clear description of cellular phenomena taking place in the male germline stem cell (GSC) niche using a long-duration live imaging approach. The AUs report that, as previously published, GSCs can be lost and replenished by symmetrical divisions in adult testes. In addition, the AUs identify a prevalent number of de-differentiation occurrences responsible for nearly 75% of the replenishment events. This novel finding is of intertest to the field and makes the publication of the work worthwhile. However, there are serious issues with the presentation of the results, the writing of the text and the discussion of some of the results. To follow are my comments, some of which are critical for the validity of the conclusions put forward by the AUs.

1- My first criticism is the quality of the written text. I might be wrong, but my feeling is that the MS has been rushed to submission. This is a pity, as the results are interesting and well documented (the quality of some of the movies is outstanding). Reading throughout the MS has taken me a great deal of effort and not much of pleasure. I would suggest the AUs the following:

- To add subheadings in the Results section. This should help conveying the message and, for instance, to discriminate whether the AUs are talking about wild type testes, testes subjected to Bam overexpression or whether they are presenting data related to de-differentiated GB.

Thank you, and we appreciate this reviewer for taking time digging our previous version and making helpful suggestions. We have added subheadings in the Result section for clarity.

- Some sections of the Results and Discussion look like bullet points, lacking contextualization and connection with the rest of the text. See for instance lines 247-270. Most likely, a new reorganization of the Results and Discussion with subheadings will help sorting out this.

- Lines 44-45 and 51-52 are repeated.

- In the text, Table S4 is cited after Table S1, with no mention to Tables S2 or S3 in between. This is confusing.

- The last paragraph of the Results section may belong to the Discussion?

Thank you. We recognized these portions were confusing and unstructured. We now have revised the text.

- I find the first paragraph of the Discussion difficult to follow. It starts by talking about GSCs and their predominant asymmetric division. Next, the AUs mention the generality of stem cell loss in tissues as a consequence of different insults or cell senescence and conclude that there must be "additional" mechanisms to ensure stem cell pool maintenance. Additional to what? To asymmetric stem cell divisions? But there are plenty of stem cells that do not divide asymmetrically. I would recommend the AUs to refer only to the case of the GSCs and invoke the need for additional (here you do necessitate other mechanisms to account for the recovery of lost GSCs) mechanisms. Next, they could mention the importance for other stem cell niches known to undergo cell replacement.

We have rewritten and restructured the entire discussion for clarity.

2- I am missing the discussion of an important discovery reported in the Results section. First, if one adds up the division events that give rise to new GSCs, they are more frequent than those responsible for the loss of GSCs (Fig. 1F). This would imply that the number of GSCs within the niche increases with time. But this is contrary to what happens in living animals. Thus, is this an artefact of the culturing protocol? This is potentially worrying, as such a conclusion would question the main finding of the work, i.e., the existence of de-differentiation events responsible for a large proportion of GSC replenishment.

Thank you for raising this point. We have now assessed the frequency of "loss" and "gain" across all of the genotypes as we have performed additional imaging analysis in other genetic backgrounds and lines as requested by Reviewer 1 (see response to Reviewer 1,

point 1 and Figure 1A). The overall frequencies calculated across all genotypes is 13.8% “loss” vs 13.9% “gain” (Figure 1A), consistent with relatively stable GSC numbers across an animal’s lifetime. We believe that there is generally technical difficulty in detecting all of the “loss” events, which contributed to the original undercounting of “loss” events.

3- In the baz RNAi experiments. The AU should show the degree of Baz downregulation. It is difficult to interpret their results without knowing how much baz or Baz is being removed.

We used a Baz RNAi line has been functionally validated in our previous study and we cited the paper in the revised manuscript.

4- I would not call 2.29 ± 1.88 hours or 2.1 hours ± 1.72 “immediate” or “promptly” entry into mitosis. There are reintegrated GSCs that require nearly 6-7 hours to divide. This is nearly 50% of the time the AUs claim it takes a GSC to divide.

We agree on this point and have revised these descriptors accordingly.

5- Line 124: In legend to Table 3 there is reference to the de-differentiation of a 4-cell SG..

Thank you. We revised the citation.

6- Legend to Fig. 1F). How is the “average distribution of divisions” calculated?

We apologize the pie charts shown in old Fig 1F are confusing.

We have changed this data presentation to new Figure1A table to avoid complication.

7- Table S2: What does “no division” mean in the Symmetric renewal category?

No division means that we did not detect division after niche reentrance by the cell. This definition has now been added to the Table legend.

8- The legend of Figure 4 and the diagram 4C indicates “Note that Drosophila GSCs do not have a detectable G1 phase". This is at odds with other literature that has detected GSCs at G1 phase (“green” and “black” cells). Moreover, the example provided by the authors shows two non-mitotic green GSCs for the whole duration of the recording (on top of the hub). In addition, the live imaging strategy used by the authors does not allow to detect “black” cells (end of G1) due to the lack of a counterstaining for all germline cells – this limitation should be acknowledged and the “lack of G1 phase” statements reviewed.

Thank you for raising this point - we have now carefully revisited the legend and previous studies. We defined the “green” cells to be S-phase as typically DNA synthesis immediately starts even prior to cytokinesis in GSCs. However, we agree that we cannot exclude the possibility that a minor fraction of GSCs is arrested in G1. We removed the lack of G1 phase statement.

9- Movies S6 and S7 depict cases of symmetric divisions with a GSC loss result. S7, however, just shows the loss of a GSC without its previous division. A better example should be provided, including its previous division.

Thank you for raising this point. Interestingly, for all 37' loss' events without associated divisions, we failed to detect previous mitosis. This may indicate that the “loss” occurs in senescent cells or in cells at a certain cell cycle stage (such as G2), which is why we could not capture the last mitosis within 16 hours of the live session. We have now added a brief description of this phenomenon in the results section which reads as follows :

“Interestingly, for all observed loss events without associated divisions (n=37 total), we failed to detect previous mitosis. This may indicate that the “loss” occurs in senescent cells or in cells at a certain cell cycle stage (such as G2), which is why we could not capture the last mitosis within 16-hour of the live session.”

10- Figure 2 depicts a dedifferentiation case both as raw images and a 3D reconstruction. The number of frames of both depictions are different and the tiles for the same timing do not seem to match between them.

We have now matched time frames, thank you.

11- Figure S5 shows quantifications of GSCs, GB and SGs numbers. These results are not mentioned or discussed in the text.

We now discuss this figure in the text, thank you.

12- bam-Gal4 is used to drive Baz-RNAi (Fig. 5). This Gal4 is only expressed in 8- and 16-cell cyst stages. As the manuscript focuses on dedifferentiation events of earlier spermatogonia stages, this should be properly discussed, as its modifies the interpretation of the results.

All reviewers raised the same concern, and we completely agree with this point. We have conducted the same experiment now with the germline nosGal4 driver, which is highly

expressed at the 1-2 cell stage. Using nosGal4, we observed the same dedifferentiation defect, now shown in Figure 4F

Below is the response for reviewers' comments. Reviewer comments are blue, our responses are black.

Reviewer #1 (Remarks to the Author):

REVIEWER 1 RESPONSE TO REBUTTAL: There is a SINGLE movie with spaghetti-squash mCherry in the revised paper (Movie S14). The midbody is not apparent in the purported Gb gaining access to the niche. Even in the stills of this movie (Fig. S3B), I cannot see the midbody between the purported GSC-Gb pair. I am not convinced that these two germ cells are clonally related. As such, I am not confident in GSC-division outcomes listed in Figure 1A for nos-myrGFP plus Sqh-mCherry. As a result, I am not convinced of the author's conclusions and other conclusions are possible/probable.

The fundamental problem with their imaging approach still exists – most of the imaging is performed with stocks that do not mark the midbody and as I have written above, their live imaging with spaghetti-squash mCherry is not presented in a convincing manner. If the authors want an example of how GSC-Gb living imaging with a midbody marker should be presented, I refer them to Lenhart (PMID: 26143993) "Movie S1. Mitosis through Abscission in a Wild-Type GSC Reveals Two Phases of Abscission Delay" and the stills for this movie in Fig. 1E1-E6. I also have strong reservations about emphasising the greater number of hours of their imaging than the Lenhart paper when the most of Bener et al live imaging did not have a midbody marker. Unless the live imaging methodology includes a midbody, it is impossible to prove that a GSC is attached to a Gb. Imaging midbodies has been instrumental in determining clonal relationships between female germline stem cells (GSCs) (Mathieu PMID: 35587967), male GSCs (Lenhart PMID: 26143993), male somatic cells in the testis (Price PMID: 36898376), and feminized somatic cells of the testis (Roach PMID: 40600835) to name a few pertinent examples. Without labeling the midbody, Inabu and colleagues cannot distinguish between a Gb and a 1-cell spermatogonia, despite the new analyses presented in the rebuttal. As such, the new analyses written in this rebuttal are not scientifically robust and other interpretations are possible or probable.

REVIEWER 1 RESPONSE TO REBUTTAL: Again because of the flawed methodology in the live imaging, the authors cannot prove that that are studying symmetrically renewing Gbs or 1-cell spermatogonia and this additional analysis is not meaningful. Because of the weakness in the approach through this manuscript, alternate conclusions are probable. If the authors want to challenge the current state of the field, then they need to perform live imaging with the right tools.

REVIEWER 1 RESPONSE TO REBUTTAL: I am still not satisfied with the methodology used by the authors. If they want to test their hypotheses in a robust manner, they will need to repeat all their live imaging with a midbody marker.

We thank for additional recommendation for this reviewer. We agree that the movie we used for previous revision was not clearly showing midbody. Since most of the differentiation cases are detected from post-cytokinetic GBs, we did not detect midbody in these cells possibly because it is already degraded at the time of migrating back to the hub (https://pubmed.ncbi.nlm.nih.gov/24227883/). We have conducted more live imaging sessions and found several examples showing abscission events prior to migration (now shown in Figure S3B). Moreover, we replaced the typical dedifferentiation movie with clearer example (new Figure S3C). We also added symmetric self-renewal example with interconnecting midbody is present (new Figure S3D), for comparison. This genotype was helpful to strengthen our conclusions.

The relevant portion is as follows:

"Notably, the different results observed among studies are unlikely to be caused by the usage of different genotypes. We tested a range of fluorescent markers, including genotypes used in previous studies, such as nosGal4 > UAS-GMA used in Sheng et al [18], Figure S3A, Movie S10), and Sqh-mCherry (used in Roach et al [22], Figure S3B, Movie S11). We continued to observe the dedifferentiation events from post-cytokinetic GB stage in which midbody remnant is no longer present (Figure S3C, Movie S12). This stage was clearly distinguishable from symmetric self-renewal cases (Figure S3D, Movie S13)."

Moreover, we included 10 selected images in the data repository. Overall results of this genotype are now shown in Figure 1A table. Replaced Figure 1A and Figure S3B-D are shown below.

Figure 1A

A

Genotypes	nosGal4> GFP-αTub	nosGal4> UAS-myrGFP	nos-myrGFP	nos-myrGFP plus Sqh-mCherry	nosGal4 > UAS-GMA	Sum	Frequency
Asymmetric division	140	110	64	77	52	443	70.2%
Symmetric renewal	7	5	7	11	4	34	5.4%
Dedifferentiation	20	9	18	12	7	66	10.5%
Symmetric differentiation	12	11	8	9	9	49	7.8%
Loss	8	12	6	8	5	39	6.2%
Sum	187	147	103	117	77	631	
# of scored testis	30	26	21	21	10	108	
**Dedifferentiation**	**10.7%**	**6.1%**	**17.5%**	**10.3%**	**9.1%**	**10.5%**	

Figure S3B-D

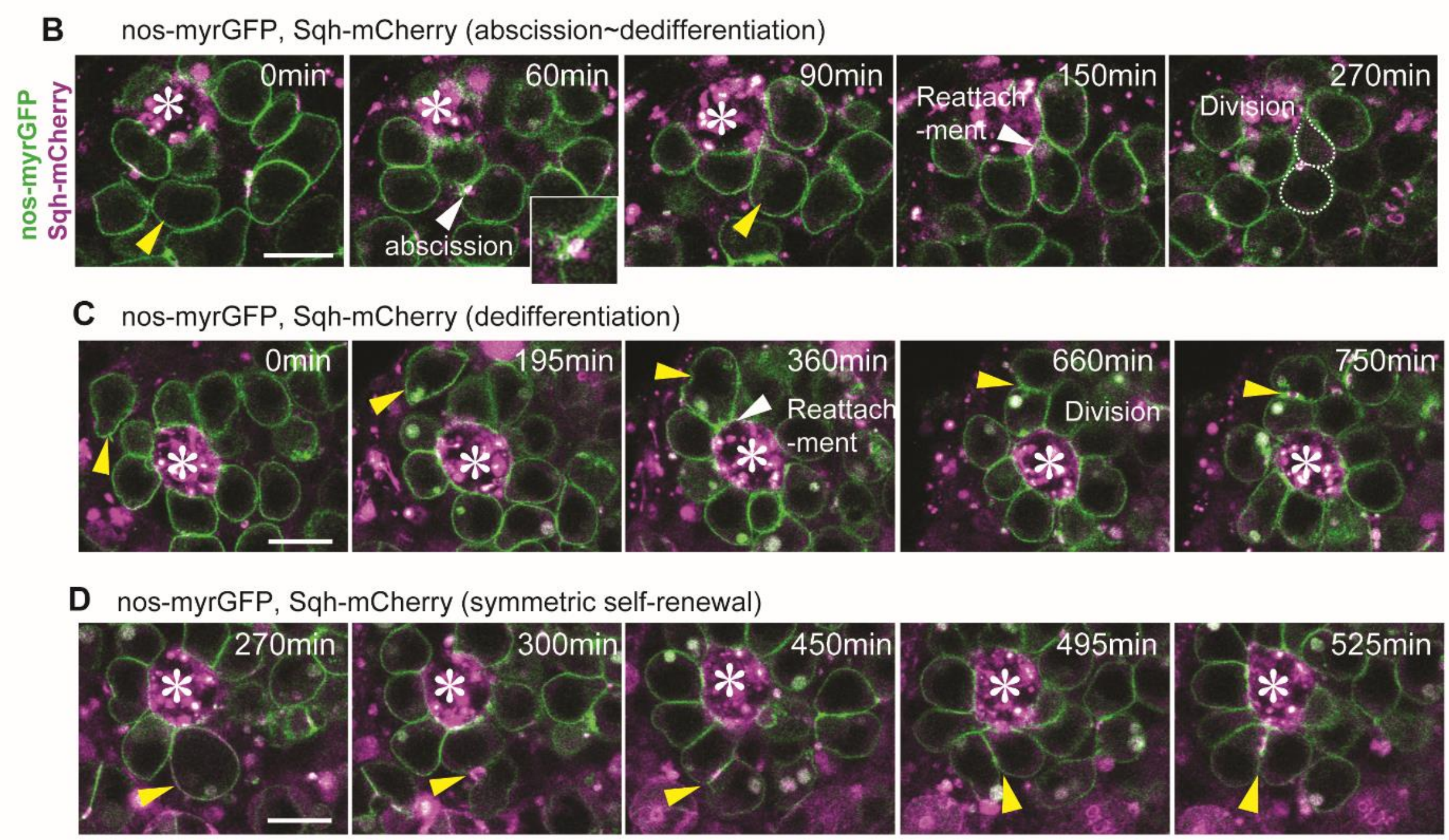

I also take issue with the new nomenclature "pre-cytokinetic Gb and post-cytokinetic Gb". The field does not use these terms. A Gb is only a Gb when it is attached to a GSC. After abscission, it is a 1-cell spermatogonia.

We could not find the definition "1-cell spermatogonia" suggested by this reviewer in published articles. Since we consider that using two different names for same GB (before and after cytokinesis) will be confusing, we remained the definition as shown in our original manuscript. However, we can revise this if editor recommends.

Reviewer #2 (Remarks to the Author):

The article titled, “Stem cells resume asymmetric division upon niche re-entry through reactivating the centrosome orientation checkpoint” was originally submitted with the title “Stem cells resume asymmetric division upon niche re-entry”, by Bener et al. It describes the behavior of gonialblast (GB) dedifferentiation and links it to the preferential niche attachment of GBs during the G2 phase. The authors utilized long-term time-lapse imaging ex vivo with a high-sensitivity confocal microscopy technique to monitor the cell division cycles of germline stem cells (GSCs) in Drosophila testes. They expressed GFP-tagged α-tubulin along with a membrane marker and used cell cycle phase marking techniques such as Fly-FUCCI to correlate the cell cycle stages of GBs undergoing dedifferentiation. During the study, the authors also described the rates of different types of GSC divisions and their loss from the niche.
In the revised version, the authors included a thorough reanalysis of the data, which help them to amend some of the conclusions. They have also included a new experimental data using nosGal4 driver as per the suggestions of the previous review. The data presented in the manuscript is comprehensive and rigorously analyzed using standard statistical methods. The authors further validated their observations using alternative techniques to support the conclusions. The study makes a significant technical advancement by achieving, for the first time, a uniform time-lapse interval of 10 minutes over 16 hours, which enabled effective comparative analysis across different genetic backgrounds. The manuscript is now rewritten with a better flow and several portions are now modified to conform with the earlier review comments. The data is effectively organized into six figure panels with adequate illustrations for non-expert readers.

I have earlier opined that GB dedifferentiation is critical for stem cell maintenance in both ovaries and testes and it happens through the reattachment of some of the GBs. However, it is still unclear what determined this reattachment. The authors found that a delayed centriole dynamics and membrane association could trigger a COC checkpoint and arrest the GBs in the G2 phase, facilitating the back transition. They propose that a centriole checkpoint (COC) mechanism could arrest GBs in the G2 stage to facilitate this process. The authors also hypothesize that GB cells retain the same factors as GSCs, enabling them to revert to stem cell fate. Thus, attachment to the niche emerges as the key driver of this process. The revised manuscript now presents a compelling case for an intriguing new hypothesis.
I find the manuscript is now well composed with clearly constructed hypothesis and the results are discussed with a correlated hypothesis. The conclusions are justified and well organized. Therefore, I have no major comment against the results section of the manuscript.

However, the discussion section is still weak. It merely summarizes the observations without clear interpretations. It will useful if the authors link the key observations of the study to other systems and discuss how their observation may open a new proposal and how they are distinct from the previously published data. For example, the data about the proposed role of COC needs to be discussed with the insipient merits and demerits of the data. In some sense the authors need to provide a balanced critique of their experimental observations.

Also, the discussion needs to be structured according to a theme. For instance, the key issue examined in the study is how the apparently differentiated GBs regain their stem-cell like property. One possibility demonstrated by the study could suggest an invocation of COC due to delayed attachment of the centriole. How strong or weak is the evidence? Also, how is this attachment could be delayed? Author can discuss these issues in a structured manner to provide a future roadmap based on their observations.

We deeply thank this reviewer for giving us encouraging comments and critical recommendations for constructive discussion section. We completely agree that our previous discussion section was not very meaningful. We rewrote discussion covering recommended points, which read as follows:

"**Discussion**

Asymmetric division is the predominant mode of division in *Drosophila* male germline stem cells (GSCs), ensuring the production of one stem cell and one differentiating daughter cell. However, stem cells can be lost from tissues during normal cellular turnover or under adverse conditions such as irradiation, chemotherapy or other drug treatment, injury and metabolic effects[21,26-28]. Therefore, maintenance of the stem cell pool and tissue homeostasis requires additional mechanisms beyond asymmetric division. In several stem cell systems, lost stem cells can be replenished through dedifferentiation[29]. However, molecular and cellular mechanism of these processes, and the behavior of newly replenished cells after replacing original stem cells have remained unclear.

In the *Drosophila* testis stem cell fate is tightly coupled to physical position relative to the niche. Cells inside the hub-adjacent niche adopt stem cell identity, whereas cells displaced outside the niche initiate differentiation. Consequently, dedifferentiation (the fate change) in this system is coupled with physical relocation of the cells migrating back into the niche[17]. Therefore, we can assess the events by observing the niche for long term. Such relocation may transduce

distinct environmental signal, resulting in the relocated cells to acquire stem cell fate again. Similarly, fate plasticity in diverse systems is nevertheless governed by local microenvironmental cues. In regenerative contexts such as heart or limb regeneration, injury-induced signals act over short distances to promote dedifferentiation and proliferation in situ (reviewed in [29,30]). The signaling mediated dedifferentiation in these systems resembles the niche-relocation mediated dedifferentiation in the *Drosophila* testis, suggesting that conserved principles may govern stem cell replenishment across tissues and organisms.

Using long-term live imaging of the testicular niche with multiple fluorescent markers, we show that dedifferentiation is the predominant mechanism for stem cell replenishment even under steady-state conditions. Moreover, we show that dedifferentiating GSCs exhibit a strong bias toward late G2 phase and typically undergo cell division shortly after reattachment to the hub. This cell-cycle bias is likely imposed by reactivation of the centrosome orientation checkpoint (COC), which arrests cells in G2 during the dedifferentiation process. Upon niche reentry, centrosomes rapidly reorient toward the hub, allowing cells to enter mitosis with correctly aligned spindles and to resume asymmetric division.

In steady state conditions, COC prevents mitotic entry when centrosomes are not correctly positioned near stem cell-niche interface, thereby ensuring correct spindle orientation and asymmetric division [9,16]. In normal conditions, this COC is exclusively active in GSCs and it is immediately deactivated in GBs [16], suggesting that specific signal may be activating COC in GBs to induce dedifferentiation. Although the signal has not been identified, it would be intriguing if such signal is commonly used for inducing dedifferentiation in different systems.

Coupling dedifferentiation to COC activation can be an effective strategy to ensure the spindle orientation of newly replenished stem cells. We could not specify if this COC reactivation is required for dedifferentiation machinery or it is a consequence of reactivating stem-like feature in dedifferentiating cells prior to niche reentry. We show that the polarity protein Baz, a key component of centrosome positioning in the COC pathway[11], is required for efficient dedifferentiation. However, because Baz has multiple cellular functions, it remains possible that Baz depletion affects centrosome orientation and dedifferentiation through distinct mechanisms. Thus, whether the observed G2 arrest is mechanistically coupled to migration or instead serves to prevent premature mitotic entry before niche reattachment remains unsolved.

Future experiments manipulating cell-cycle progression independently of migration will be critical to distinguish between these possibilities.

Our study suggests that the dedifferentiated GSCs promptly restart asymmetric division and we detected no significant differences in proliferative capacity between dedifferentiated and native GSCs. Previous studies have shown that dedifferentiated GSCs behave differently from native GSCs, although they have reported conflicting results [9,19]. For example, Cheng et al. showed that dedifferentiated cells frequently exhibit centrosome misorientation and reduced mitotic activity [9], whereas Herrera et al. reported that dedifferentiated GSCs induced by repeated feeding–starvation cycles display increased mitotic activity [19]. The discrepancies among studies likely arise from differences in the cellular origin or experimental conditions used to induce dedifferentiation. Notably, earlier studies primarily examined dedifferentiated cells derived from 4- to 8-cell spermatogonial cysts, whereas our study focused on earlier-stage germline blast cells (GBs), which we identified as the major source of dedifferentiation. Using a previously established lineage-tracing method employed in earlier studies, we similarly observed a high frequency of centrosome misorientation (Figure 5D), indicating that dedifferentiated cells exhibit distinct properties depending on their stage of origin.

In summary, our study demonstrates that dedifferentiated stem cells fully regain the capacity for asymmetric division and normal proliferation, despite potential disturbances in the inheritance of intrinsic factors. Our findings highlight that asymmetric division is subject to exceptionally stringent regulation. Further studies will be necessary to identify the signal that induces this process and how the signal interconnects cell cycle checkpoint and migration. Given the high frequency of dedifferentiation under physiological conditions, identifying the mechanisms that specifically regulate this process will be essential for understanding how tissues are maintained over the long term."

Reviewer #3 (Remarks to the Author):

The authors have answered satisfactorily all of my concerns.

Thank you.

Reviewer #4 (Remarks to the Author):

Bener et al.'s paper demonstrated that dedifferentiation occurs from “single” gonialblast cells (Gbs) under both physiological and regenerative conditions. Furthermore, their research provides a mechanistic perspective on the dedifferentiation process; it explicitly clarifies that this form of dedifferentiation is different from that of GBSs, which remain connected to GSCs after GSC division—a phenomenon previously documented and called symmetric renewal. As a result, I consider Bener et al.'s manuscript suitable for publication in Communications Biology as a novel discovery.

Although this is a minor point, I agree with a criticism from reviewer #1, who claims that conducting live imaging with specific cellular bridge markers between germline stem cells (GSCs) and gonialblasts (GBs), such as midbody ring (Pavarotti) and/or adducin, would improve the clarity of the findings. Presenting live imaging results would better illustrate the authors’ assertion that dedifferentiation occurs from “single” GBs, if possible. Even if it is technically difficult, I believe the paper’s conclusions would remain unaffected, since Bener et al. clearly showed that “single” GBs undergo dedifferentiation in their Figures 1D and 3C.

We thank the reviewer for understanding our main conclusions. As noted in our response to Reviewer #1, we agree that the representative image using the Sqh-mCherry marker in the previous version was not sufficiently clear. We have replaced it with clearer examples, which further support and clarify our conclusions.

This figure shows “post-abscission” GB moves to the niche to become GSC (new Figure S3B, below).

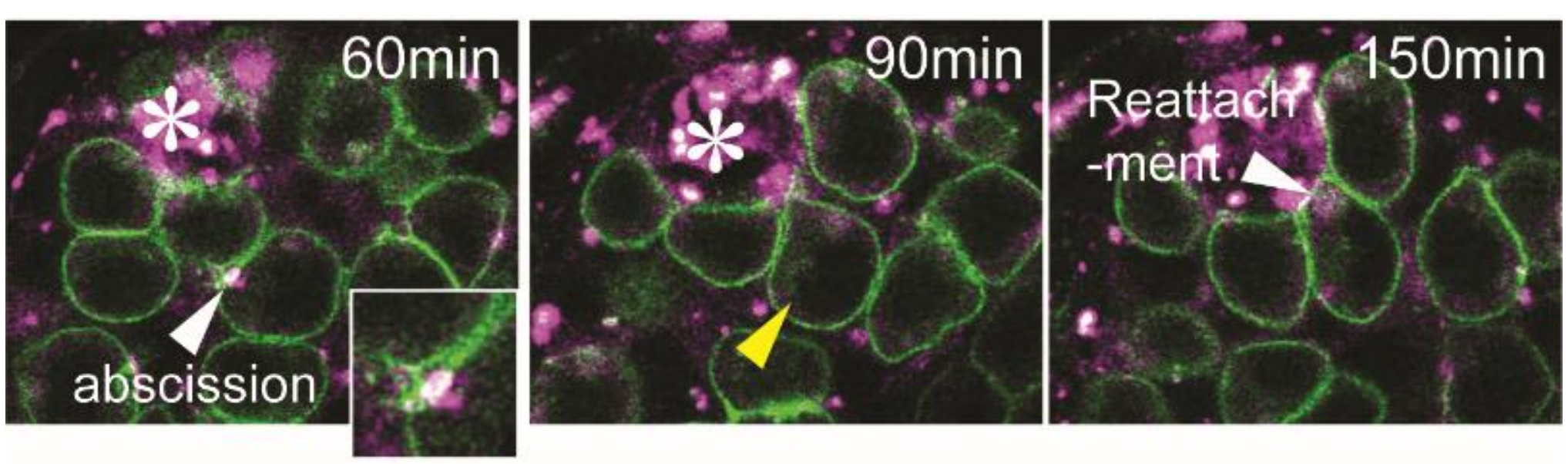

Most cases of dedifferentiation are initiated by wandering single GB (new Figure S3C below). We did not detect midbody in the dedifferentiating cells in such cases.

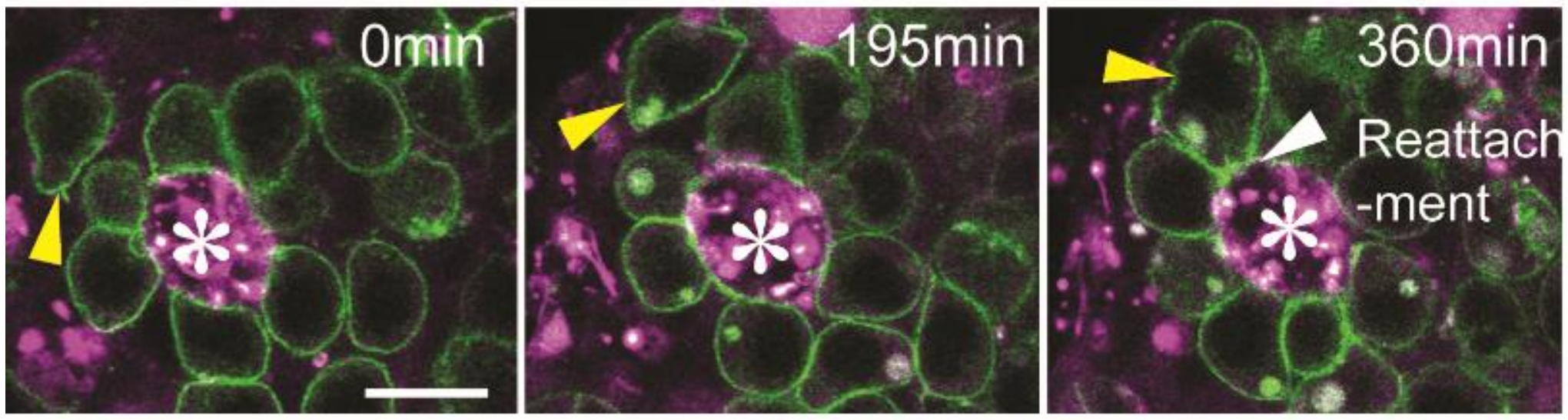

We also added a symmetric self-renewal case in which an interconnecting midbody remains between two daughter cells after reattachment to the hub (new Figure S3D, below).

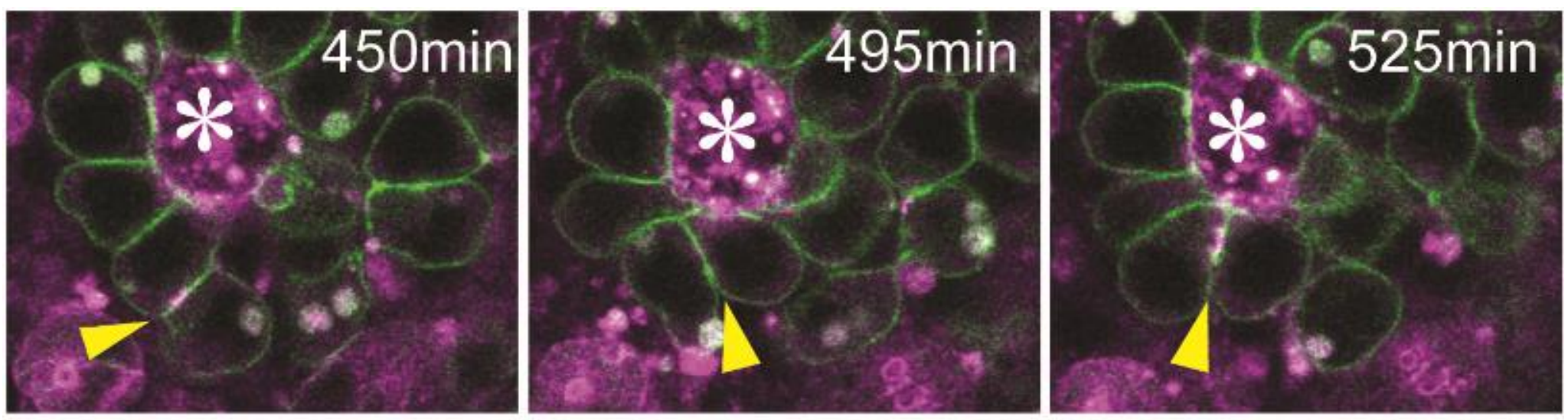

We hope now these images are clear enough to support our conclusion. Respective movies are provided as new Movie S11, S12, S13.

Below is the response for reviewers' comments. Reviewer comments are blue our responses are black.

Nonetheless, I believe the discussion section requires further restructuring. Firstly, the initial and second paragraphs are superfluous, as they reiterate information from the introduction. Secondly, the authors should commence each paragraph with their findings from this study to facilitate a clearer discussion of the results. This approach would enhance the clarity of the flow and the overall presentation of the discussion points.

Thank you very much for this suggestion. We rewrote the discussion entirely. We summarized the point in the first paragraph and discussed each finding in following paragraphs as suggested. We hope this version is more meaningful (inserted below).

"Discussion

In several stem cell systems, lost stem cells can be replenished through dedifferentiation[26]. However, it remains unclear whether a common mechanism underlies this process, specifically, how the stem cell niche senses vacancy and transmits signals to differentiating cells to switch their fate back toward stemness. In addition, the functional properties of dedifferentiated stem cells relative to native stem cells remain poorly understood.

In this study, using long-term live imaging of the *Drosophila* testicular niche, we show that dedifferentiating GSCs exhibit a strong bias toward late G2 phase and typically undergo cell division shortly after reattachment to the hub. This cell-cycle bias is imposed by reactivation of the centrosome orientation checkpoint (COC), which is a polarity checkpoint active specifically in stem cells, indicating that the dedifferentiating cells regain at least one stem cell-specific characteristic, COC, prior to niche reentry.

In steady state conditions, COC prevents mitotic entry when centrosomes are not correctly positioned, thereby ensuring correct spindle orientation and asymmetric division [9,16]. Our live monitoring revealed that majority of dedifferentiated GSCs also divide with correctly oriented spindles, similarly to normal GSCs, indicating that the reactivation of COC prior to niche reentry prevents mitotic entry of dedifferentiated cells until correct establishment of apical polarity and centrosome positioning. Supporting this idea, we observed prompt centrosome repositioning in these dedifferentiating cells upon niche reentry.

Our findings suggest the existence of at least two unidentified signals. First, under normal conditions, the majority of GBs do not possess COC[16]. Because GSC depletion induces

widespread COC activation in GBs located outside the niche, there must be a signal that senses stem cell loss and activates COC in these cells. The identity of this signal remains unknown. Notably, COC reactivation also occurs in dedifferentiating GBs under steady-state conditions, suggesting that this signal may act locally in response to niche vacancy.

Second, we propose an additional signal that is activated in dedifferentiated GSCs upon niche reattachment to promote rapid centrosome reorientation, and release cells from COC-mediated arrest, enabling immediate mitotic entry. Future studies will be required to define the nature of these signals and how they coordinate dedifferentiation and asymmetric division.

Coupling dedifferentiation to COC activation can be an effective strategy to ensure the spindle orientation of newly replenished stem cells. Although, we could not determine if this COC reactivation is required for dedifferentiation machinery or it is a consequence of reactivating stem-like feature in dedifferentiating cells prior to niche reentry, we show that the polarity protein Baz, a key component of centrosome positioning in the COC pathway[11], is required for efficient dedifferentiation. Because Baz has multiple cellular functions other than COC, it remains unclear whether Baz depletion affects centrosome orientation and dedifferentiation through distinct mechanisms. Thus, whether the observed G2 arrest is mechanistically coupled to migration or instead serves to prevent premature mitotic entry before niche reattachment remains unsolved. Future experiments manipulating cell-cycle progression independently of migration will be critical to distinguish between these possibilities.

Lastly, our work suggests that there are no significant differences in proliferative capacity between dedifferentiated and native GSCs. Previous studies have shown that dedifferentiated GSCs behave differently from native GSCs, although they have reported conflicting results [9,19]. For example, Cheng et al. showed that dedifferentiated cells frequently exhibit centrosome misorientation and reduced mitotic activity [9], whereas Herrera et al. reported that dedifferentiated GSCs induced by repeated feeding–starvation cycles display increased mitotic activity [19]. The discrepancies among studies likely arise from differences in the cellular origin or experimental conditions used to induce dedifferentiation. Notably, earlier studies primarily examined dedifferentiated cells derived from 4- to 8-cell spermatogonial cysts, whereas our study focused on earlier-stage germline blast cells (GBs), which we identified as the major source of dedifferentiation. Using a previously established lineage-tracing method employed in earlier

studies, we similarly observed a high frequency of centrosome misorientation (Figure 5D), indicating that dedifferentiated cells exhibit distinct properties depending on their stage of origin.

In summary, our results highlight that asymmetric division is subject to exceptionally stringent regulation via activating polarity checkpoint prior to the niche reentry. Furthermore, we demonstrate that dedifferentiated stem cells fully regain the capacity for asymmetric division and normal proliferation rate, despite potential disturbances in the inheritance of intrinsic factors. Given the high frequency of dedifferentiation under physiological conditions, identifying the mechanisms that specifically regulate this process will be essential for understanding how tissues are maintained over the long term."
